# Towards observing interstitial hydrogen in solution in metal lattices

**DOI:** 10.1038/s41586-026-11238-4

**Published:** 2026-11-04

**Authors:** Chao Huang, Saiyu Liu, Wenting Lv, Chun Chen, Shangkun Shen, Ranming Niu, Zhao Xu, Yanqi Tu, Siwen Shen, Cheng-Yun Wu, Pang-Yu Liu, Kewei Gao, Yu Yang, Hung-Wei Yen, Xinping Mao, Engang Fu, Julie Cairney, Xiaolu Pang, Rongjian Shi, Eason Yi-Sheng Chen

**Affiliations:** 1 School of Materials Science and Engineering, State Key Laboratory of Nuclear Power Safety Technology and Equipment, University of Science and Technology Beijing, Beijing, China; 2 Australian Centre for Microscopy and Microanalysis, The University of Sydney, Sydney, New South Wales Australia; 3 School of Aerospace, Mechanical and Mechatronic Engineering, The University of Sydney, Sydney, New South Wales Australia; 4 National Key Laboratory of Computational Physics, Institute of Applied Physics and Computational Mathematics, Beijing, China; 5 School of Materials Science and Engineering, Nanyang Technological University, Singapore, Singapore; 6 Institute for Carbon Neutrality, University of Science and Technology Beijing, Beijing, China; 7 Energy Research Institute @ NTU, Nanyang Technological University, Singapore, Singapore; 8 Department of Materials Science and Engineering, National Taiwan University, Taipei, Taiwan; 9 High Entropy Materials Center, National Tsing Hua University, Hsinchu, Taiwan; 10 State Key Laboratory of Nuclear Physics and Technology, School of Physics, Peking University, Beijing, China

**Keywords:** Metals and alloys, Characterization and analytical techniques

## Abstract

Hydrogen-bearing materials are central to low-carbon energy technologies^1^, but hydrogen itself is difficult to detect and localize at the nanoscale^2–6^. This hinders the development of advanced materials with better efficiency. Atom probe tomography (APT) can map hydrogen in three dimensions and at high resolution, yet characterization of dilute, mobile, interstitial hydrogen in metals has remained challenging because of low solubility, the ambiguity owing to hydrogen trapping by structural defects, as well as the artefacts introduced during hydrogen introduction^4–6^. Here we address whether interstitial hydrogen in solution in materials can be distinguished from defect-trapped hydrogen in APT. Using deuterium as a proxy for hydrogen, together with face-centred cubic (FCC) multi-principal-element alloys (MPEAs) containing coherent FCC precipitates, we show that deuterium atoms reproducibly concentrate in hydrogen-affinitive phases in a manner consistent with theoretical predictions. We further resolve differences in deuterium content between phases with distinct local chemistries and crystal structures, consistent with their different calculated hydrogen solution energies. These findings demonstrate that APT can move beyond identifying hydrides and solute-decorated defects to examine hydrogen in solid solution, providing new insights into hydrogen partitioning in structural alloys and informing materials design for hydrogen-related applications.

## Abstract (web summary)

Using deuterium as a proxy for hydrogen, atom probe tomography provides experimental evidence for interstitial hydrogen in solid solution across different FCC phases, consistent with theoretical predictions of hydrogen affinity, revealing hydrogen partitioning in structural alloys.

## Main

Hydrogen (H) and hydrogen-containing compounds such as ammonia offer promising routes for low-carbon energy storage and utilization, supporting global decarbonization efforts^1^. Developing hydrogen energy technologies requires a fundamental understanding of how hydrogen interacts with materials. Hydrogen embrittles high-strength alloys in transport and storage systems^2,7,8^; its behaviour in metals determines the capacity and reversibility of solid-state storage^9^; hydride formation in zirconium-alloy cladding threatens fission reactor integrity^10^; and isotope retention is critical to fusion viability^11^. Across all of these applications, knowing where hydrogen resides in materials is essential.

Hydrogen in crystalline materials exists in three principal states: dissolved as an interstitial solute in solid solution^8^, bonded to hydrogen-affinitive elements as thermodynamically stable hydrides (for example, titanium hydride)^12,13^ or trapped at crystal defects, including vacancies^14,15^, dislocations^3,16^ and grain boundaries^3,17^. Experimentally, these states can be analysed by APT^2,4^, which uses time-of-flight mass spectrometry to identify hydrogen with nanoscale spatial resolution. In practice, deuterium (^2^H or D) is often introduced instead of protium to distinguish genuine hydrogen signals from background hydrogen in the instrument, which is predominantly ^1^H (refs. ^4,5^).

Among the three states, hydrides and defect-trapped hydrogen have been investigated more extensively by APT. Hydrides are identifiable through their characteristic stoichiometries^12,13^ and defect-trapped hydrogen can often be co-localized with solute-decorated defects (for example, Cottrell atmospheres such as that of carbon)^3,16^. When defects are not decorated with identifiable elements, associating hydrogen with defect traps becomes challenging because such defects are ‘invisible’ in APT, unless correlative techniques such as transmission electron microscopy (TEM) are used^16,18^.

By contrast, characterizing hydrogen in solid solutions is particularly challenging for several reasons. First, the solubility of hydrogen is generally low in many alloys; for example, body-centred cubic (BCC) iron typically contains only a few tens of atomic parts per million (at. ppm) of hydrogen^2,7,8^, which approaches the APT detection limit^6,19^. Second, unlike hydrogen in stable hydrides, interstitial hydrogen is highly mobile and can diffuse to the surface and desorb before analysis^20,21^; cryogenic sample transfer (cryo-transfer) for APT mitigates, but does not eliminate, such signal loss^4,20,22^.

A further complication arises from the methods used to introduce hydrogen into the needle-shaped APT specimens. Electrochemical charging is commonly used^2,4^, but it can impose extremely high hydrogen fugacity on the specimen and generate large numbers of vacancies—so-called ‘superabundant vacancies’^14,23^. Further artefacts can be introduced during specimen preparation: electron-beam or ion-beam irradiation in scanning electron microscopy (SEM) or focused ion beam (FIB) can also create vacancies that subsequently trap hydrogen^15^. Careful interpretation of APT hydrogen maps is therefore required whenever SEM-FIB processing has been used.

In this study, we use a series of FCC alloys as model systems to address this challenge. Compared with BCC alloys, FCC lattices offer higher hydrogen solubility and lower diffusivity^7,24^, improving signal-to-noise ratio and reducing diffusion-related losses. Our results provide experimental evidence for interstitial hydrogen in FCC metallic solid solutions and establish a methodological basis for robust, artefact-aware APT characterization of hydrogen in materials.

## Deuterium in precipitate-free FCC MPEA lattice

Throughout this study, APT tip specimens were electrolytically charged with deuterium, which saturates specimens more efficiently than gaseous charging, while reducing corrosion concerns^3,4^. Voltage pulsing rather than laser pulsing was used for all APT analyses to minimize ambiguity in deuterium signal identification against instrumental background hydrogen^4,6^. Full experimental details are provided in Methods.

**Fig. 1:**
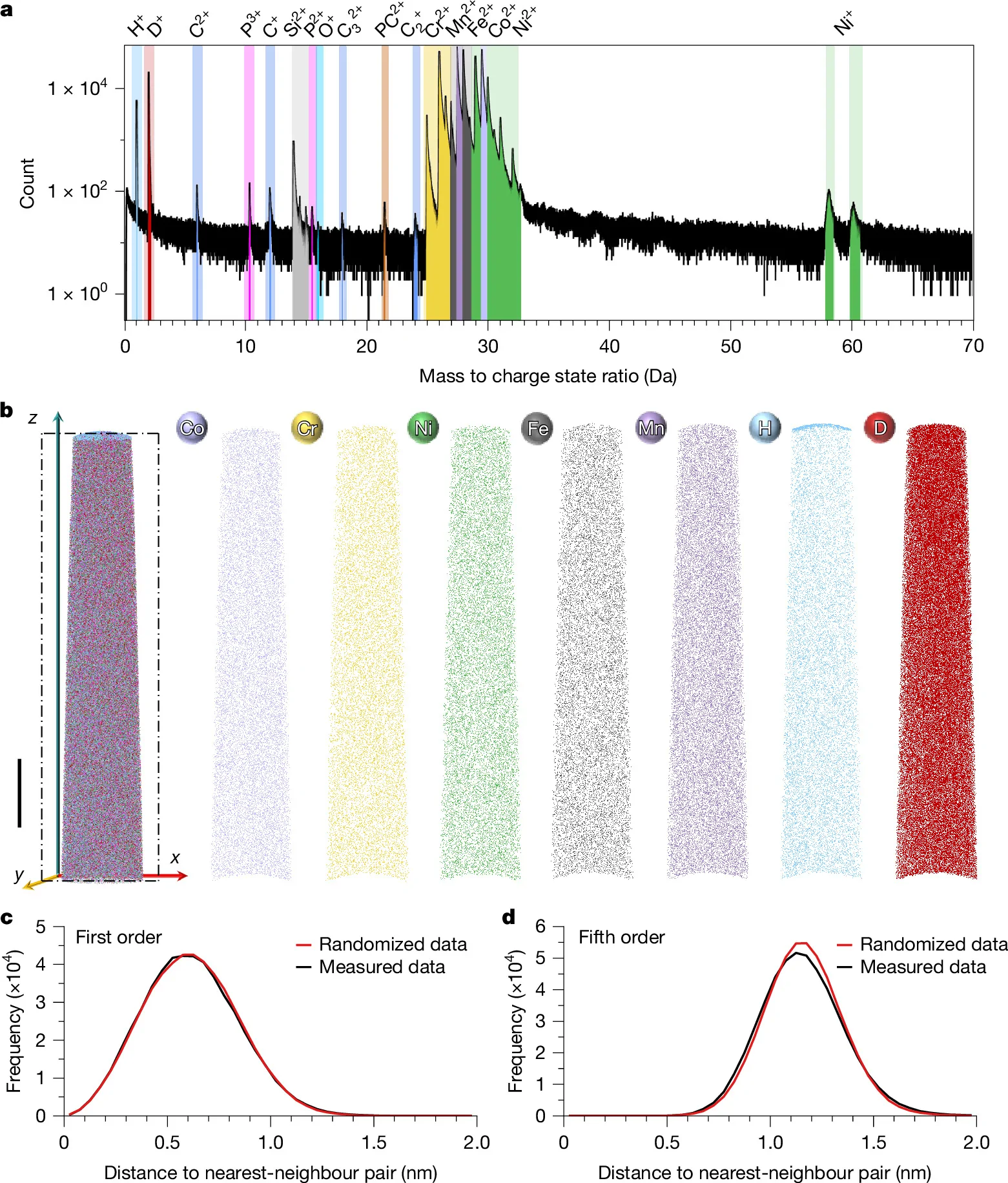
Voltage-pulsing APT results of deuterium-charged single-phase CoCrNiFeMn MPEA. **a**, Mass spectrum, showing a notable deuterium peak at 2 Da with respect to the peak of background hydrogen at 1 Da. **b**, Three-dimensional reconstruction of all elements (left) and the 30-nm slice views of the principal elements, hydrogen (H, representing background) and deuterium (D, representing signal). **c**, The statistics of the atomic distance of solute D with the first nearest D neighbour as well as the randomized counterpart with the same number of D atoms in the same volume. **d**, The repeated analysis of **c** except using fifth nearest D neighbour. Scale bar, 50 nm.

**Extended Data Fig. 1:**
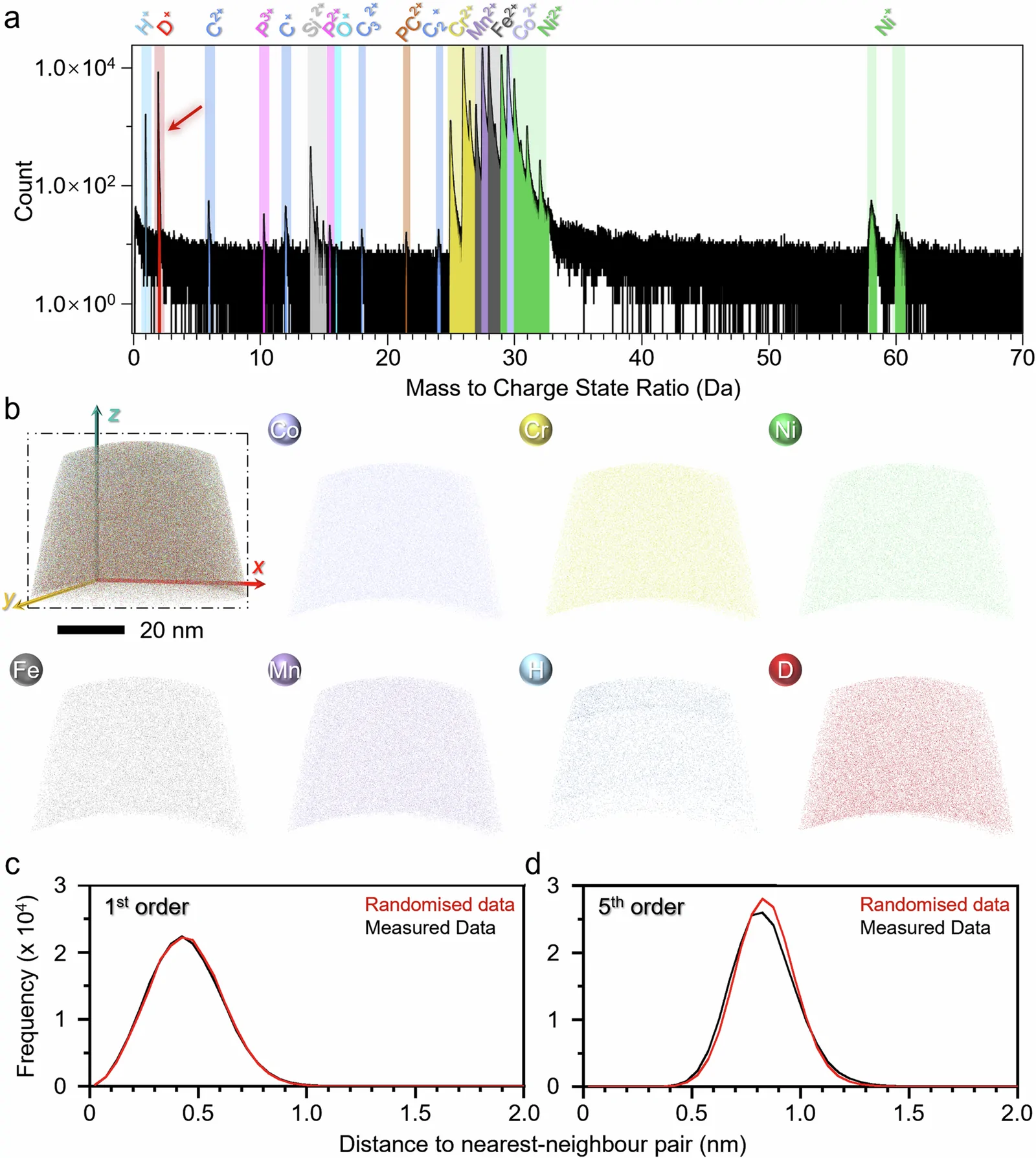
Reproduced voltage-pulsing APT data of D-charged single-phase CoCrNiFeMn specimen corresponding to Fig. 1. **a**, Mass spectrum with the presence of D peak (red). **b**, Three-dimensional reconstruction of all elements and the 20-nm slice views of the principal elements (Co, Cr, Ni, Fe, Mn), background hydrogen (blue) and deuterium (red). **c**, Distribution of D–D pair distances for the first nearest D neighbour, compared with a randomized dataset, both peaking at approximately 0.4 nm, indicating a random D distribution. **d**, Same analysis for the fifth nearest D neighbour, with a most probable distance of approximately 0.8 nm, consistent with random, solid-solution-like interstitial deuterium.

As shown in Fig. 1, we first present results from an equiatomic CoCrNiFeMn MPEA, first developed by Cantor et al.^25^. This alloy is known to have a locally distorted lattice, which can more effectively hinder hydrogen diffusion and thus reduce signal loss^26^. Immediately after D charging, we spent approximately 3 s to remove the D-charging electrolyte and then quenched the charged tips in liquid nitrogen (LN_2_) to ‘freeze’ the deuterium in the MPEA. Once LN_2_ evaporation stopped—indicating thermal equilibration between the tips and LN_2_—the specimens were transferred into the APT system using the cryo-transfer procedure described previously^3^. As shown in Fig. 1a, the APT mass spectrum of the D-charged CoCrNiFeMn specimen exhibits a clear D peak in red at mass-to-charge ratio of 2 Dalton (Da), distinct from the peak of ambient hydrogen (predominantly protium, ^1^H) at 1 Da, together with the peaks from the principal alloying elements. The overall D concentration reaches 1.3 atomic percent (at.%), equivalent to 473 parts per million by weight (wt. ppm). Figure 1b presents the three-dimensional reconstruction of the APT dataset and 30-nm-thick two-dimensional slices showing the spatial distributions of the major elements, together with H in light blue and D in red. The H map shows a hydrogen-rich cap at the specimen apex, which arises from the field-induced migration and evaporation of ambient hydrogen adsorbed on the tip surface during APT analysis^4^. By contrast, the D map shows a relatively homogeneous and random distribution, consistent with what is expected for interstitial hydrogen in solid solution. To confirm reproducibility, we repeated this experiment on a second tip and obtained a comparable result, as shown in Extended Data Fig. 1.

Because deuterium can segregate to defects that are not visible in APT (ref. ^16^), we carefully evaluated whether the observed deuterium distribution is truly random. We performed nearest-neighbour analyses of deuterium atoms, as shown in Fig. 1c,d, for the first and fifth nearest deuterium neighbours, respectively. This approach detects clustering by comparing interatomic distances between deuterium atoms in the experimental dataset with those in a randomized dataset of equivalent size^19,27^. A marked shift towards shorter distances indicates clustering, whereas agreement with the random distribution is consistent with deuterium occupying a large population of equivalent interstitial sites^27^.

**Extended Data Fig. 2:**
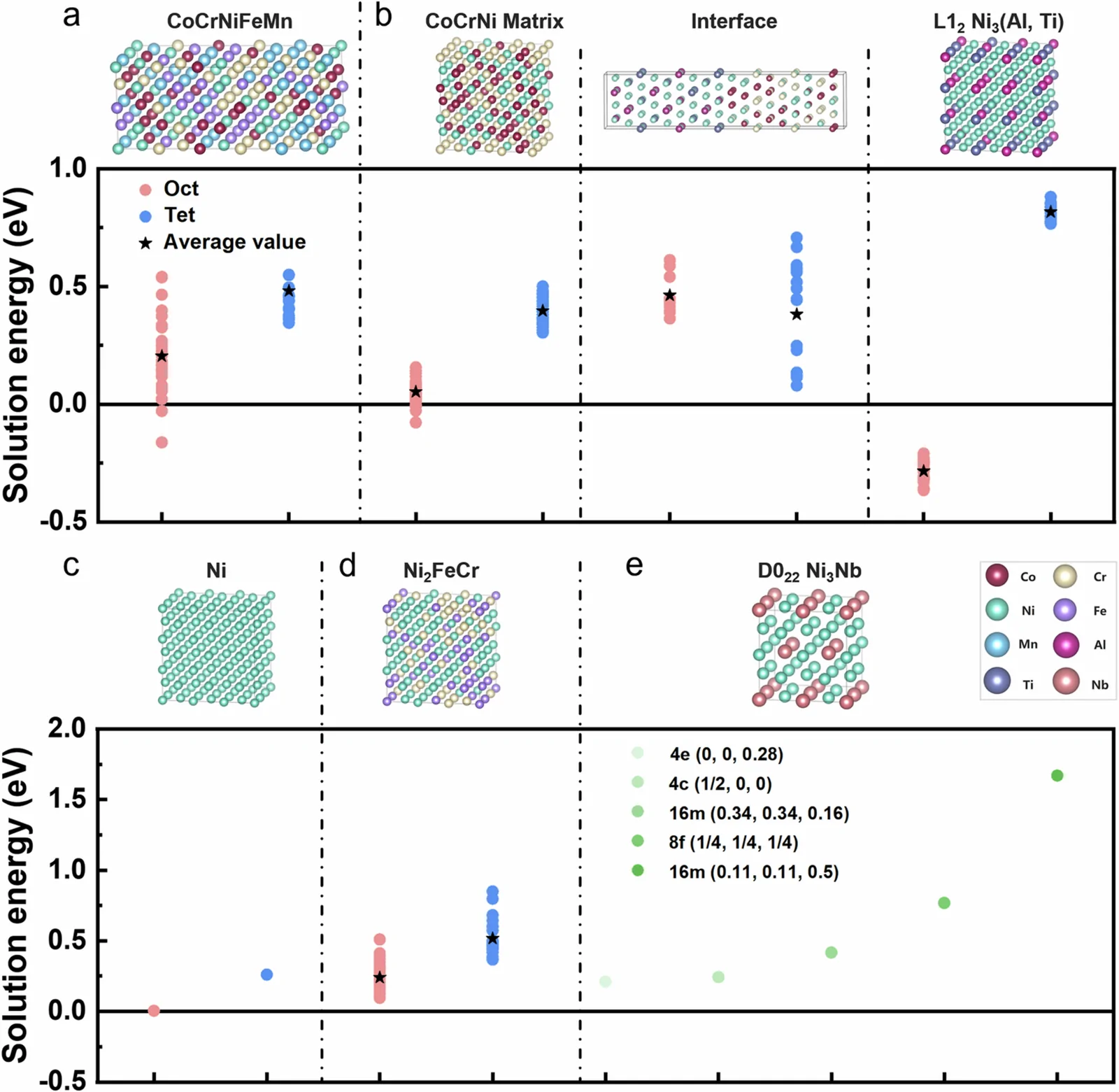
DFT calculations on hydrogen solution energies at various interstitial sites. **a**, Atomistic structure used in calculation and hydrogen solution energies at the octahedral interstitial sites and tetrahedral interstitial sites in the bulk of CoCrNiFeMn FCC matrix. **b**, Atomistic structure and hydrogen solution energies at octahedral and tetrahedral sites in the CoCrNi FCC matrix, CoCrNi ($1\bar{1}\bar{1}$)/L1_2_ Ni_3_(Al, Ti) ($1\bar{1}\bar{1}$) interface and L1_2_ Ni_3_(Al, Ti) structure. A subset of octahedral sites that are not energetically favourable for hydrogen solutions is excluded. **c**, Atomistic structure and hydrogen solution energies at octahedral and tetrahedral sites in the Ni FCC matrix. **d**, Atomistic structure and hydrogen solution energies at octahedral and tetrahedral sites in the Ni_2_FeCr FCC matrix. **e**, Atomistic structure and hydrogen solution energies at octahedral and tetrahedral sites in the D0_22_ Ni_3_Nb structure.

**Extended Data Fig. 3:**
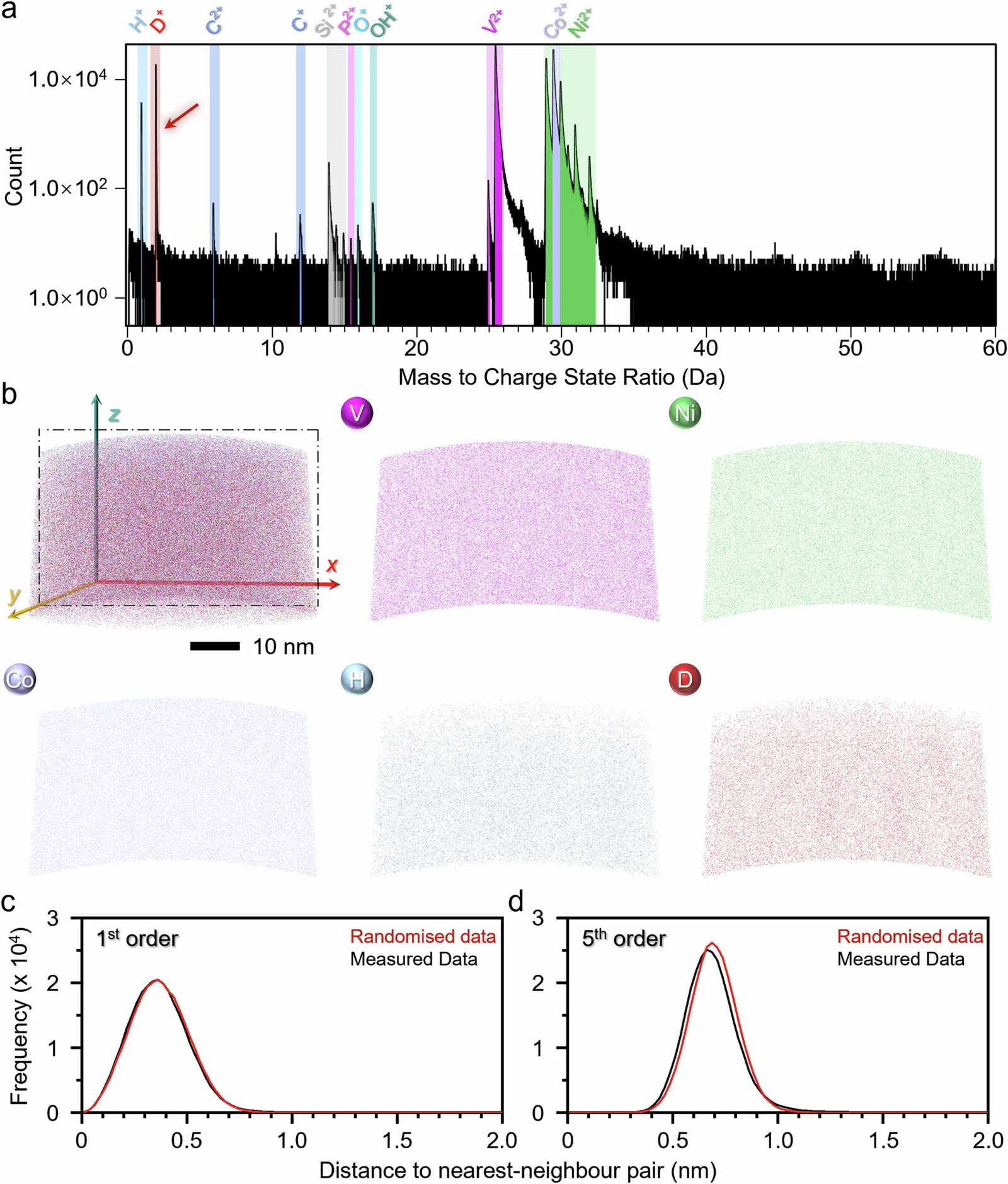
Voltage-pulsing APT data of a D-charged single-phase CoNiV specimen. **a**, Mass spectrum with the presence of D peak (red). **b**, Three-dimensional reconstruction of all elements and the 20-nm slice views of the principal elements (Co, Ni, V), background hydrogen (blue) and deuterium (red). **c**, Distribution of D–D pair distances for the first nearest D neighbour, compared with a randomized dataset, both peaking at approximately 0.4 nm, indicating a random D distribution. **d**, Same analysis for the fifth nearest D neighbour, with a most probable distance of approximately 0.7 nm, consistent with random, solid-solution-like interstitial deuterium.

Figure 1c shows the distribution of deuterium–deuterium pair distances for the first nearest neighbour. The most probable pair distance in the experimental data is close to that in the randomized dataset (both approximately 0.8 nm), indicating that the deuterium atoms are randomly distributed. We repeated the analysis for the fifth nearest deuterium neighbour (Fig. 1d) and obtained a consistent result, with a larger most probable distance of about 1.2 nm, as expected when a more distant neighbour is considered. These results are consistent with interstitial deuterium (as a proxy of hydrogen) in solid solution. Notably, hydrogen solution in the CoCrNiFeMn matrix is not highly thermodynamically favourable according to our first-principles calculations, for neither octahedral nor tetrahedral interstitial sites (Extended Data Fig. 2a). Figure 1 therefore suggests the effectiveness of cryo-APT for preserving the charged D atoms and enabling observation of their distribution in solid solution. Random D distributions were also consistently observed in solution-treated CoNiV and (CoCrNi)_94_Al_3_Ti_3_ alloys (Extended Data Fig. 3 and Supplementary Fig. 1), supporting the generality of this observation among FCC MPEAs.

## FCC MPEA with coherent L1_2_ precipitates

**Fig. 2:**
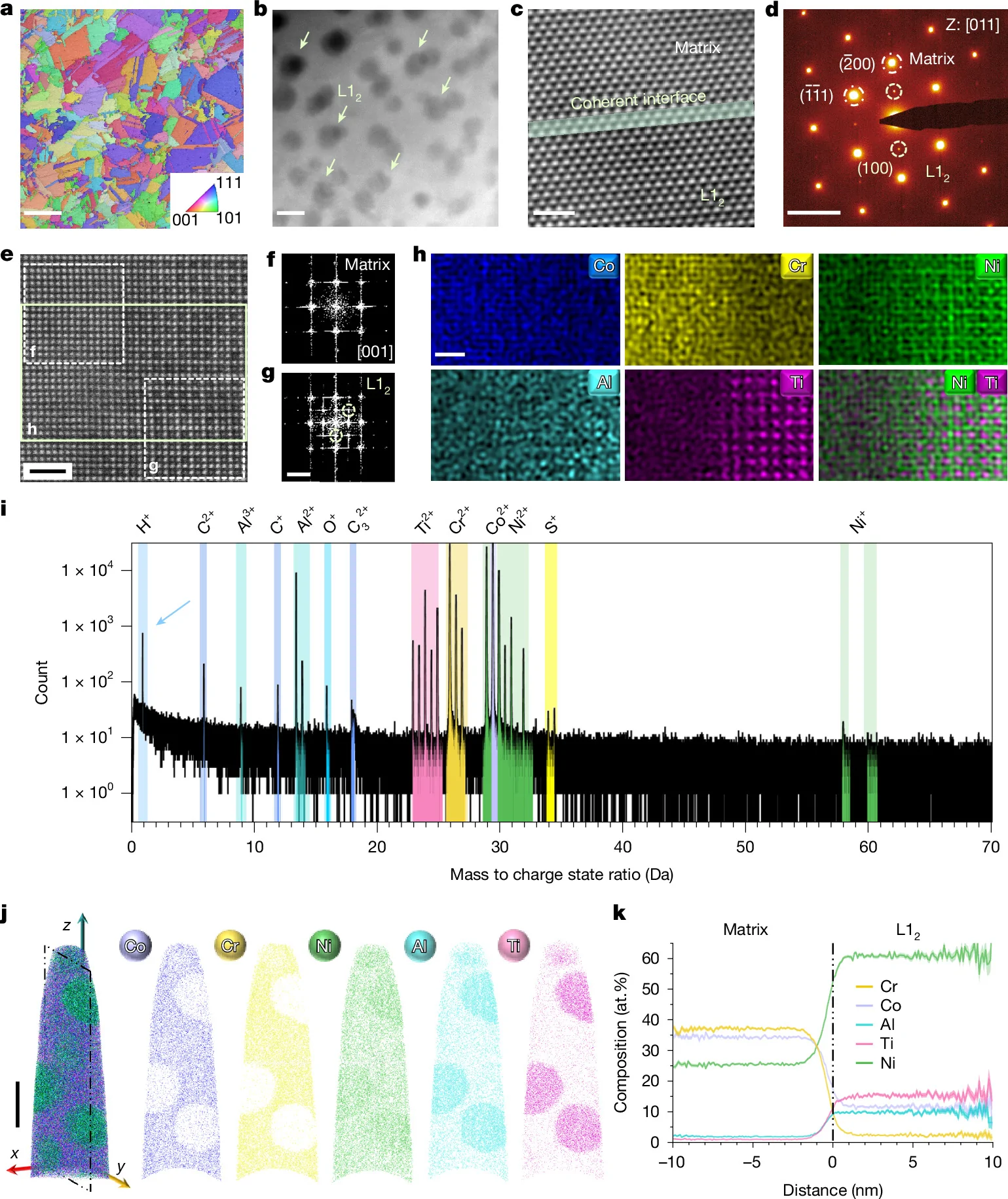
Structural and analytical analyses of deuterium-free dual-phase (CoCrNi)_94_ Al_3_Ti_3_ specimen. **a**, EBSD inverse pole figure map. **b**, STEM image, showing a high density of L1_2_ precipitates (green arrows) with an average diameter of 18.5 ± 3.5 nm. **c**, HRTEM image of the coherent interface between the matrix and L1_2_ precipitates along the [011] zone axis. **d**, SAED pattern showing the characteristic diffraction spots of the FCC L1_2_ precipitates (green), which are distinct from the FCC matrix (white). **e**, High-resolution HAADF-STEM image of the coherent interface between the FCC matrix and L1_2_ phase along the [001] zone axis, showing the analysed regions for **f**, **g** and **h**. **f**,**g**, FFT data of the FCC matrix and the L1_2_ precipitate from regions in **e**, respectively. **h**, EDS mapping results from the region in **e**, with the matrix elements (Co, Cr and Ni) and the L1_2_ precipitate elements (Ni, Al and Ti). **i**, Voltage-pulsing APT mass spectrum, showing the 1-Da peak (blue arrow) associated with background hydrogen. **j**, Three-dimensional data reconstruction (left) and the 10-nm slices of the matrix elements (Co, Cr and Ni) and L1_2_ precipitate (Ni, Al and Ti). **k**, Proxigram from the isoconcentration surfaces (isosurfaces) of 60 at.% Ni, showing the stoichiometry of Ni_3_(Al, Ti). Scale bars, 100 μm (**a**); 20 nm (**b**,**j**); 1 nm (**c**,**e**,**h**); 5 nm^−1^ (**d**,**g**).

As a similar randomness of D distribution could also arise if a large number of superabundant vacancies was introduced during electrolytic charging and act as traps^14,28^, we used a dual-phase FCC (CoCrNi)_94_Al_3_Ti_3_ MPEA containing L1_2_ Ni_3_(Al, Ti) intermetallic precipitates with highly coherent interfaces with the matrix, which provides a heterogeneous microstructure less favoured to form superabundant vacancies. The specimens were first solution-treated at 1,000 °C for 1 h to obtain a precipitate-free state and then aged at 800 °C for 5 h to induce precipitation. Structural and compositional characterization of the aged (CoCrNi)_94_Al_3_Ti_3_ FCC MPEA is shown in Fig. 2. Figure 2a presents an electron backscatter diffraction (EBSD) inverse pole figure map; a comparison of EBSD maps before and after ageing is provided in Supplementary Fig. 2, indicating that ageing to form precipitates does not substantially change the grain morphology.

Figure 2b shows a dark-field STEM image, revealing a high number density of L1_2_ precipitates with an average diameter of 18.5 ± 3.5 nm in the matrix. Figure 2c is a high-resolution transmission electron microscopy (HRTEM) image of the L1_2_–matrix interface showing a highly coherent interface. The diffraction pattern in Fig. 2d confirms the presence of L1_2_ phase within the FCC matrix. Figure 2e is another HRTEM image marking the regions analysed in Fig. 2f–h. The fast Fourier transform (FFT) pattern from the FCC matrix region (Fig. 2f) is distinct from that of the L1_2_-containing region (Fig. 2g), further confirming the presence of L1_2_ precipitates. Figure 2h shows the energy-dispersive X-ray spectroscopy (EDS) maps with the enrichment of Ni, Al and Ti in the L1_2_-containing region.

The APT mass spectrum of a deuterium-free specimen in Fig. 2i shows peaks from the major elements and a H peak at 1 Da, but no D peak at 2 Da. Figure 2j shows the three-dimensional APT reconstruction and 10-nm slices of the main elements, clearly revealing precipitates enriched in Ni, Al and Ti. Figure 2k presents a proximity histogram (proxigram) across an isoconcentration surface (isosurface) defined at 60 at.% Ni, showing the agreement between the theoretical stoichiometry and the composition of the L1_2_ precipitates measured by APT. APT reconstructions from a precipitate-free (CoCrNi)_94_Al_3_Ti_3_ specimen are provided in Supplementary Fig. 3, which show homogeneous elemental distributions and a mass spectrum similar to that in Fig. 2 and Supplementary Fig. 3, respectively, confirming the precipitation owing to heat treatment in good agreement with literature^29^.

Such high-strength, ordered FCC L1_2_ Ni_3_(Al, Ti) precipitates embedded in a high-entropy CoCrNi matrix can be more resistant to damage arising from high-fugacity electrolytic charging^30,31^. Also, this dual-phase material provides an opportunity to study interstitial hydrogen behaviour in two different FCC lattices. Because L1_2_ Ni_3_(Al, Ti) has a strong affinity for hydrogen^12,13^ (Extended Data Fig. 2b), we anticipated that such precipitates should bind hydrogen more strongly and therefore retain a higher deuterium concentration than the surrounding CoCrNi matrix.

To compliment APT analyses, we used thermal desorption spectroscopy (TDS) to compare hydrogen retention in the precipitate-free and precipitated (CoCrNi)_94_Al_3_Ti_3_ specimens on the macroscopic scale. As indicated by the arrow in Supplementary Fig. 4, the precipitated specimen shows an increased amount of hydrogen desorbed at higher temperatures, indicating the presence of microstructural features that hold hydrogen more strongly than the FCC matrix; these features are most likely the L1_2_ Ni_3_(Al, Ti) precipitates. Although the experimental approaches are different, these TDS results are consistent with the trends predicted by our first-principles calculations using density functional theory (DFT) for evaluating the solution energies of hydrogen in all relevant interstitial sites (that is, both octahedral and tetrahedral), as shown in Extended Data Fig. 2b, considering in the CoCrNi matrix, at the L1_2_–matrix interface and within the L1_2_ Ni_3_(Al, Ti) lattice. Owing to the chemical disorder, the solution energy values for hydrogen in the MPEAs are broadly distributed compared with those in pure metals. We find that hydrogen exhibits the lowest solution energy (approximately −0.28 eV) at the specific octahedral sites of the L1_2_ Ni_3_(Al, Ti) phase.

## Deuterium enrichment in L1_2_ precipitates

For the (CoCrNi)_94_Al_3_Ti_3_ specimen containing Ni_3_(Al, Ti) precipitates, we repeated most of the procedures used for the CoCrNiFeMn specimen. The only modification was an extended holding time for 10 s before LN_2_ quenching and cryo-transfer, with an intention to promote preferential deuterium desorption from the matrix, thereby enhancing the deuterium concentration contrast between phases with low (matrix) and high (precipitate) hydrogen-binding energies in the APT dataset. This controlled holding after deuterium saturation allows the charged deuterium atoms to remain mobile briefly before quenching, leading to more pronounced deuterium loss from the low-binding-energy phase than from the high-binding-energy phase. The measured deuterium distribution can reflect both thermodynamic and kinetic effects during this brief redistribution, as demonstrated in our previous work^16^.

**Fig. 3:**
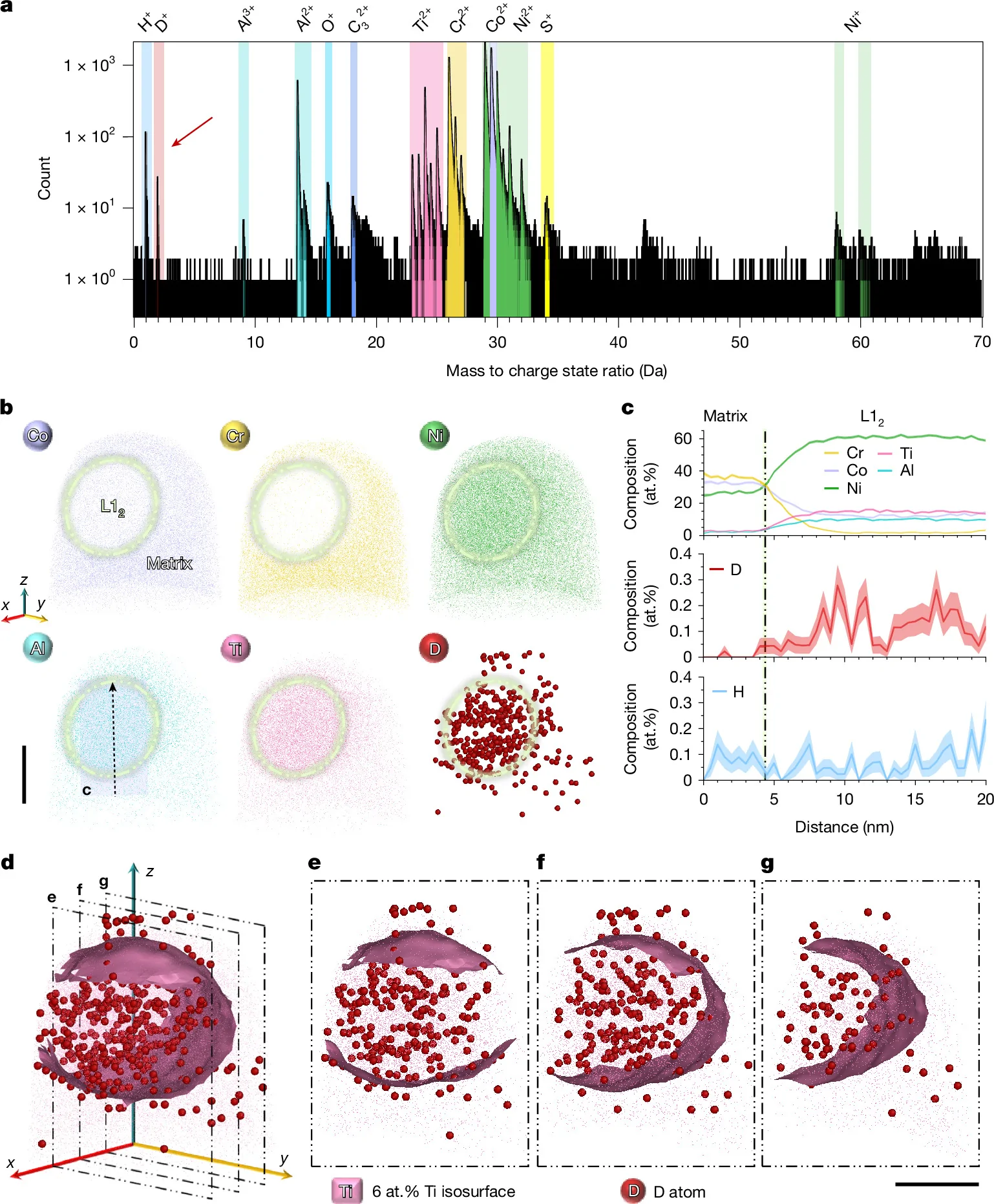
Voltage-pulsing APT results of deuterium-charged dual-phase (CoCrNi)_94_Al_3_Ti_3_ MPEA. **a**, Mass spectrum, showing a notable deuterium peak at 2 Da (red arrow) with respect to the peak of background hydrogen at 1 Da. **b**, 10-nm slice views of the principal elements in the matrix (Co, Cr and Ni) and L1_2_ precipitate elements (Ni, Al and Ti), as well as deuterium (D, representing signal) with green outlines, delineating the incorporated L1_2_ precipitate. **c**, One-dimensional profile from the region of interest noted in the Al map in **b**, across the matrix–L1_2_ precipitate interface, showing the element concentration variations corresponding to the L1_2_ precipitate as well as the D concentration (red) in the L1_2_ precipitate, distinct from the concentration variation of the background H (blue). **d**, Three-dimensional view of D atoms (red) and 6 at.% Ti isosurface (pink), as well as the locations of the slices for **e**, **f** and **g**. **e**–**g**, 10-nm slice views of D atoms (red) and 6 at.% Ti isosurface (pink). Scale bars, 10 nm.

**Extended Data Fig. 4:**
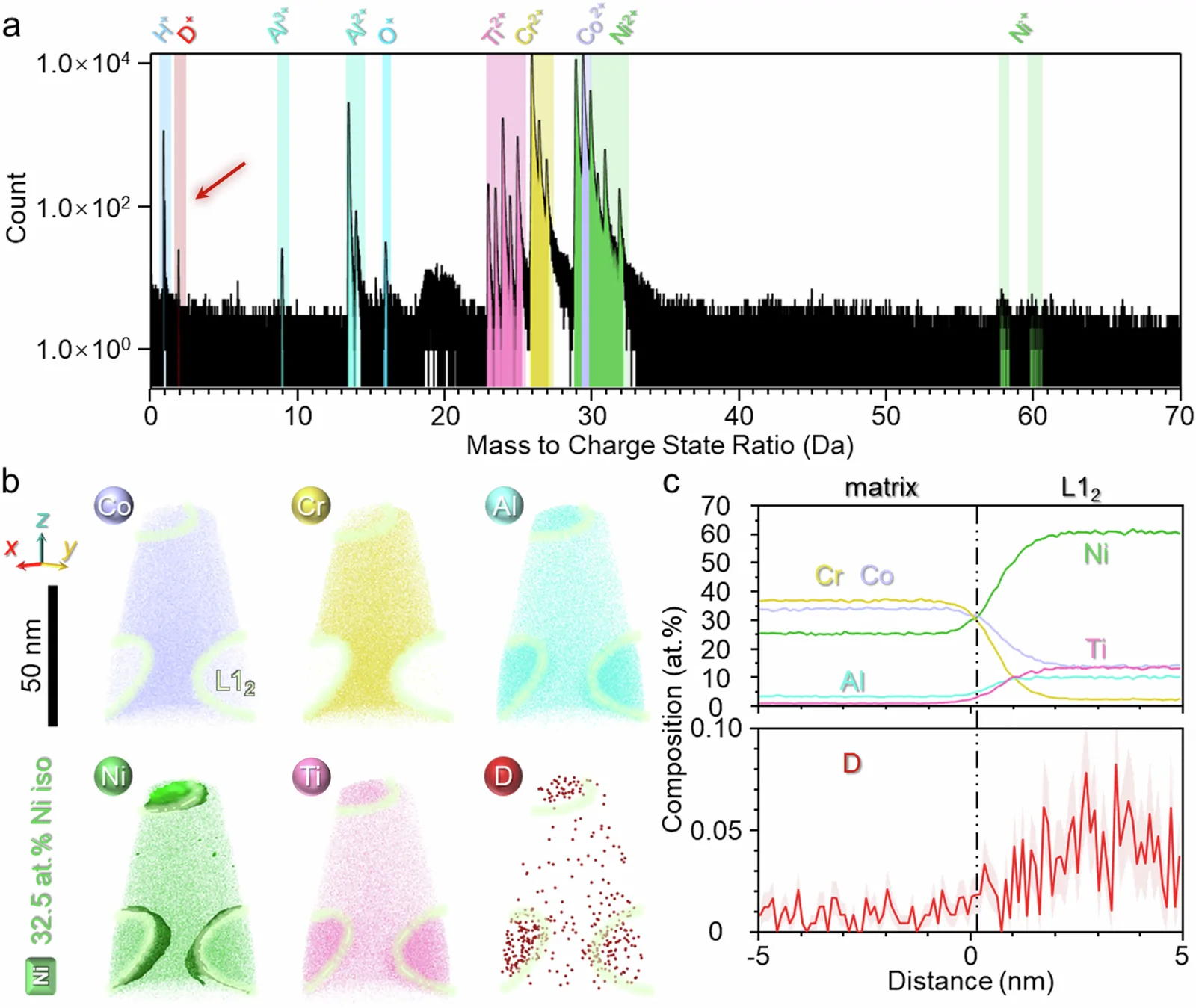
Reproduced voltage-pulsing APT data of D-charged precipitated (CoCrNi)_94_Al_3_Ti_3_ specimen corresponding to Fig. 3. **a**, Mass spectrum with the presence of D peak (red). **b**, Three-dimensional reconstructions of the principal elements (Co, Cr and Ni) and the L1_2_ precipitate elements (Ni, Al and Ti) and a 32.5 at.% Ni isosurface (green), as well as deuterium (D, red). **c**, Cumulative proxigram from the Ni isosurfaces, showing D concentration in the L1_2_ regions.

The APT results are shown in Fig. 3. The mass spectrum in Fig. 3a shows a clear D peak in red at 2 Da, together with the peaks of the major elements. Because of the controlled holding at room temperature, the D peak is less intense than that observed for the CoCrNiFeMn specimen in Fig. 1a. Figure 3b shows the three-dimensional reconstruction with the principal elements and D atoms (red), with green outlines added to enhance the visibility of the L1_2_ precipitate. A distinctly higher D concentration is observed inside the L1_2_ Ni_3_(Al, Ti) precipitate. In Fig. 3c, we plot the one-dimensional concentration profiles of D, H and the major alloying elements along a cylindrical region of interest indicated in the Al map in Fig. 3b. The D profile (red) is clearly different from the H background (blue), indicating that the D signal is not associated with the artefact related to background hydrogen. Figure 3d shows another view of the reconstruction, together with the D atoms, the Ti isosurface and the positions of the 10-nm two-dimensional slices used to generate Fig. 3e–g. We repeated the experiment on another specimen and the corresponding results (Extended Data Fig. 4) show the same D enrichment in the L1_2_ precipitates, confirming reproducibility. Taken together, these observations show that the Ni_3_(Al, Ti) L1_2_ precipitates contain a higher hydrogen (represented by deuterium) concentration than the surrounding CoCrNi FCC matrix. We interpret deuterium enrichment in the L1_2_ FCC precipitates as being predominantly associated with interstitial hydrogen in the FCC lattice, for the reasons discussed below.

First, our previous work has shown that APT can resolve D concentrated within a second phase while showing little or no D at an adjacent interface with a low elastic strain field^18^. It demonstrated that APT detected D in cementite rather than at the pearlite interface, consistent with DFT predictions for the relative hydrogen affinities of cementite, ferrite and their interface, ruling out artefacts related to ion-trajectory effects in APT (ref. ^19^). The present study has a related microstructural configuration—a hydrogen-affinitive FCC L1_2_ second phase, an FCC matrix and a coherent interface with weak local elastic strain—so a similar enrichment of D in the L1_2_ phase, rather than at the interface, is expected.

Second, to validate the low populations of vacancy traps in our specimens, we performed positron annihilation measurements on both the precipitate-free and precipitated (CoCrNi)_94_Al_3_Ti_3_ specimens, their 2%-strained counterparts (which should contain more vacancies) and pure nickel reference specimens. The results, summarized in Supplementary Table 1, show that, although the nickel references give measurable positron signals (rows 1 and 2), all (CoCrNi)_94_Al_3_Ti_3_ specimens (rows 3–6) are below the detection limit of the instrument. This indicates that the vacancy concentrations in these alloys are indeed low. Our DFT calculations also show that the vacancy formation energies in CoCrNi and L1_2_ Ni_3_(Al, Ti) are greater than 1.5 eV (Supplementary Fig. 5), indicating that the formation of a substantial equilibrium vacancy population is unlikely.

**Extended Data Fig. 5:**
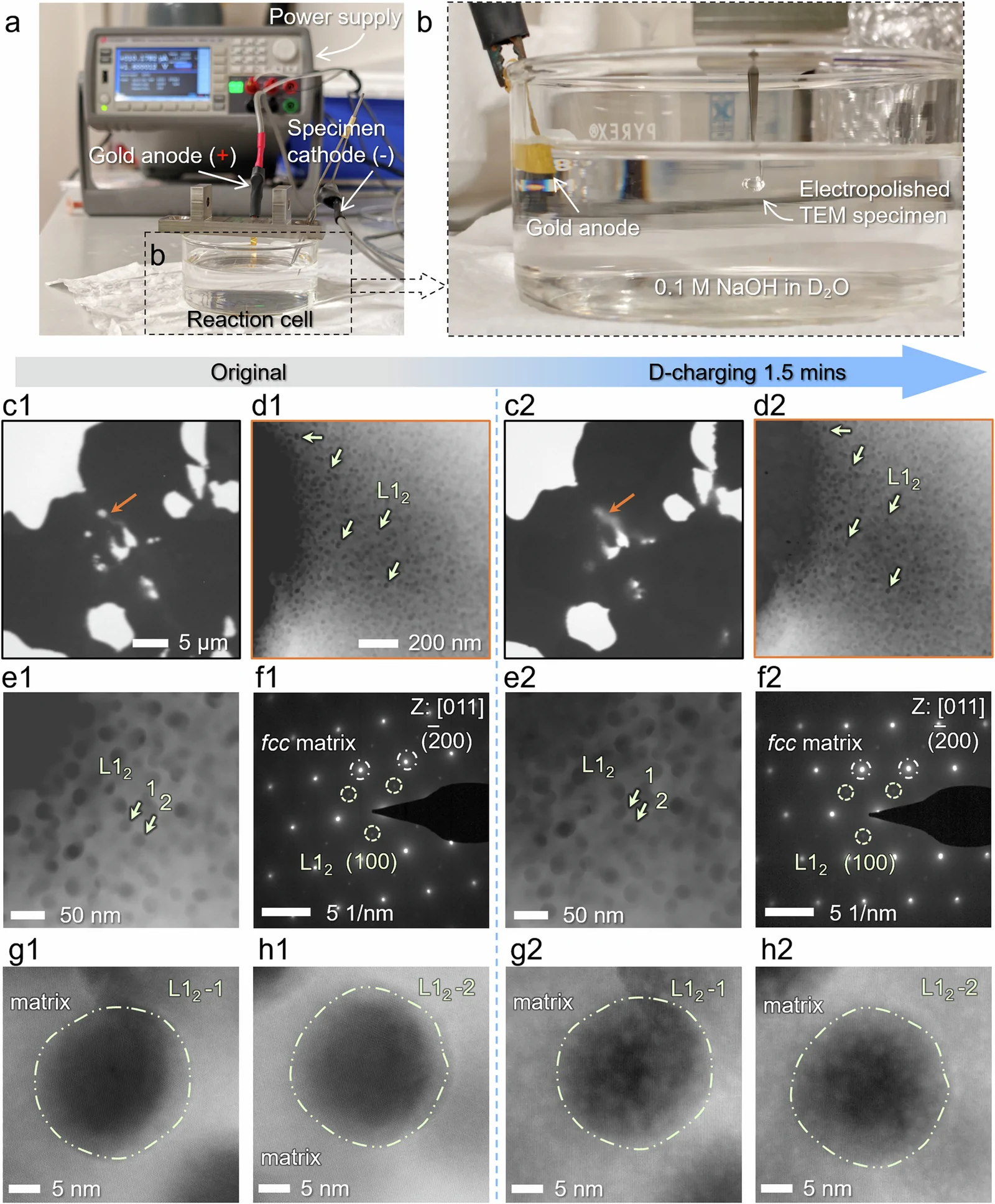
Quasi in situ TEM observations of the same region in (CoCrNi)_94_Al_3_Ti_3_ alloy during electrochemical D-charging. **a**,**b**, Experimental set-up used for quasi in situ D-charging of TEM specimens in 0.1 M NaOH prepared in D_2_O. **c1**,**c2**, Low-magnification TEM images showing the same specimen region before charging and after D-charging for 1.5 min. The arrow marks the same feature used for relocation. **d1**,**d2**, Corresponding higher-magnification HAADF-STEM images of the same area containing L1_2_ precipitates. The same precipitates were tracked throughout the charging process. No obvious microstructural changes were observed after D-charging. **e1**,**e2**, HAADF-STEM images showing the same L1_2_ precipitates before charging and after D-charging for 1.5 min. The precipitates labelled 1 and 2 were selected for repeated characterization. **f1**,**f2**, SAED patterns acquired along the [011] zone axis. Superlattice reflections associated with the ordered L1_2_ structure remain visible throughout the charging process. **g1**,**g2**,**h1**,**h2**, HRTEM images of precipitates 1 and 2. No stacking faults, dislocations or other newly introduced crystalline defects were detected after D-charging, as confirmed by the corresponding FFT patterns (data not shown).

**Extended Data Fig. 6:**
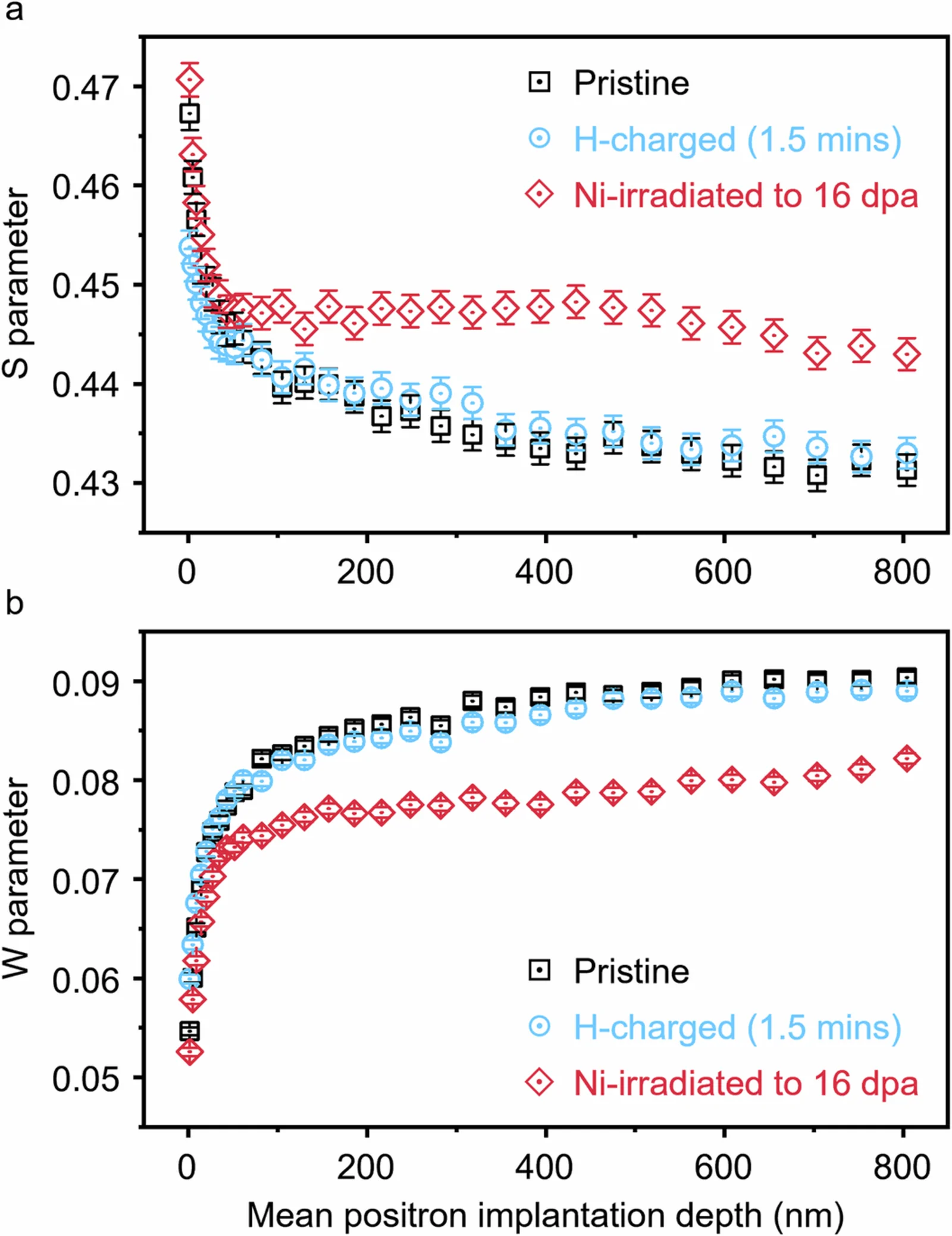
Depth-dependent Doppler-broadening positron annihilation spectroscopy results for the pristine, hydrogen-charged and Ni-ion-irradiated (CoCrNi)_94_Al_3_Ti_3_ alloy with L1_2_ precipitates. **a**,**b**, S parameter (**a**) and W parameter (**b**) as functions of the mean positron implantation depth. The H-charged specimen shows values close to those of the pristine specimen, indicating no detectable increase in vacancy-type open-volume defects after 1.5 min of H-charging process. By contrast, the specimen irradiated to 16 dpa exhibits a pronounced increase in S together with a decrease in W, indicating enhanced positron trapping at irradiation-induced vacancy-type defects.

**Extended Data Fig. 7:**
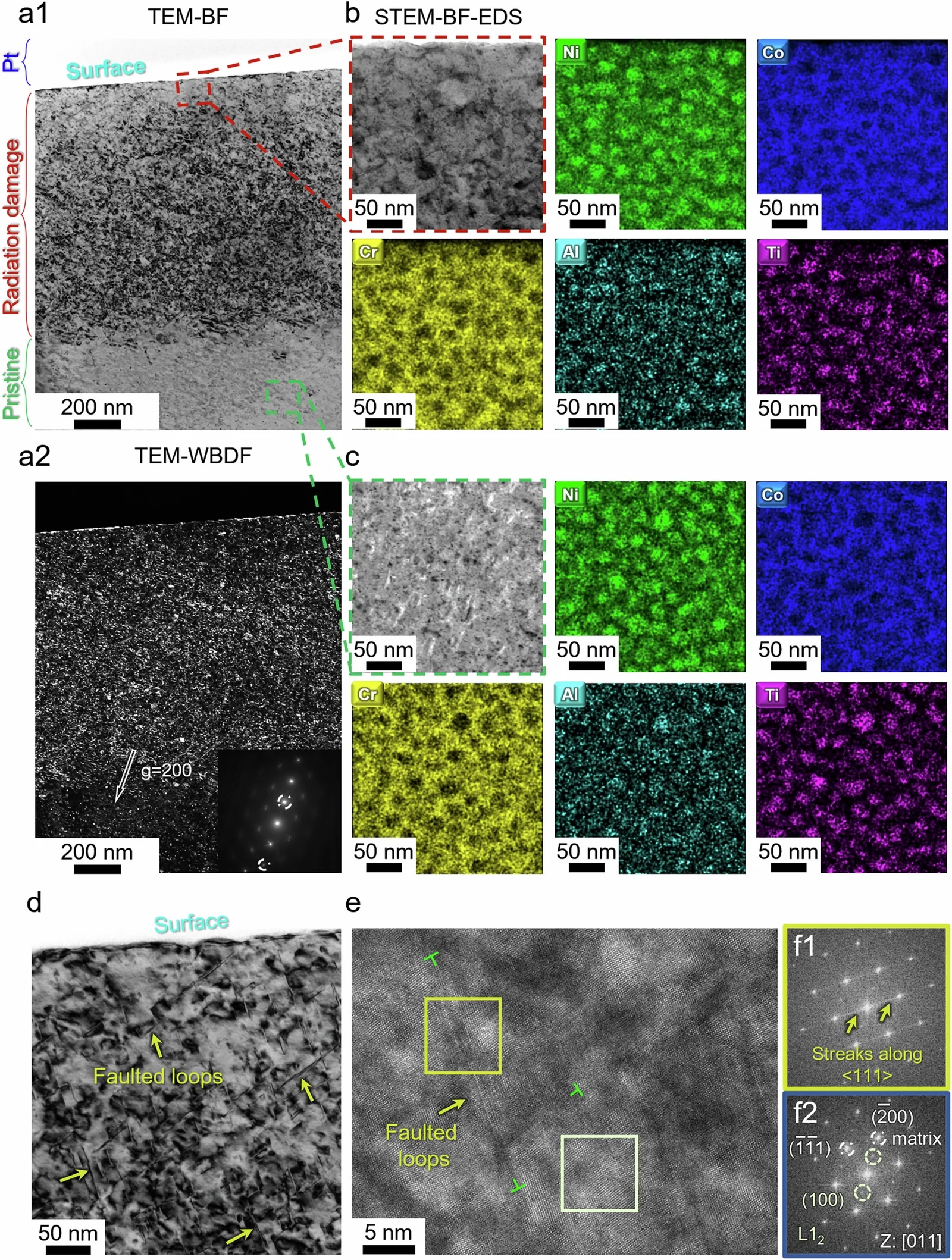
TEM characterization of irradiation-induced defects and precipitate stability after Ni-ion irradiation on a (CoCrNi)_94_Al_3_Ti_3_ alloy with L1_2_ precipitates. **a**_**1**_, Cross-sectional two-beam bright-field (BF) TEM image of the Ni-ion-irradiated specimen acquired under *g* = 200, *Z* = [011] conditions, showing a high-density irradiation-damaged layer extending from the surface to a depth of approximately 1 μm. **a**_**2**_, Corresponding weak-beam dark-field (WBDF) TEM image obtained under *g*/3*g* conditions, further revealing the dense distribution of irradiation-induced defects within the damaged region. **b**,**c**, STEM-BF images and corresponding EDS elemental maps collected from the irradiation-damaged region and the underlying pristine region, respectively. The comparable elemental partitioning between these two regions indicates that the precipitates retain their original morphology and spatial distribution after irradiation. **d**, Higher-magnification TEM-BF image of the near-surface damaged region, in which numerous faulted loops are observed. **e**, HRTEM image of a representative faulted loop. **f1**,**f2**, FFT patterns taken from the dark yellow and pale yellow-green boxed regions in panel **e**, respectively. The streaking features along the <111> direction confirm that the dominant irradiation-induced defects are Frank-type faulted loops with Burgers vectors of *b* = *a*/3<111>. No TEM-visible vacancy clusters or voids are detected in the high-magnification images.

We further examined whether the electrochemical D-charging process could introduce crystalline defects that might act as trapping sites for deuterium, as suggested in previous studies^31–34^. Quasi in situ TEM observations were performed on the same L1_2_ precipitates before and after D charging, and no stacking faults, dislocations or other newly introduced crystalline defects were detected (Extended Data Fig. 5). Also, as shown in Extended Data Figs. 6 and 7, positron annihilation spectroscopy, which is commonly used to characterize vacancy defects and hydrogen–vacancy complexes^35,36^, showed no notable increase in vacancy concentration after D charging. This contrasts with the irradiated specimen, which contains more defects (primarily vacancies) and thus exhibited higher positron annihilation signals.

The combination of DFT calculations, quasi in situ TEM observations during D charging and positron annihilation measurements suggests that the vacancy concentrations are low, so detected D is predominantly associated with interstitial lattice sites in the L1_2_ phase, although a minor contribution from non-detectable vacancies or vacancy–H_n_/D_n_ complexes, or even elastic fields or the intrinsic APT detection limitations, cannot be completely excluded^14,28,37,38^.

## Probing an FCC superalloy with dual precipitates

**Extended Data Fig. 8:**
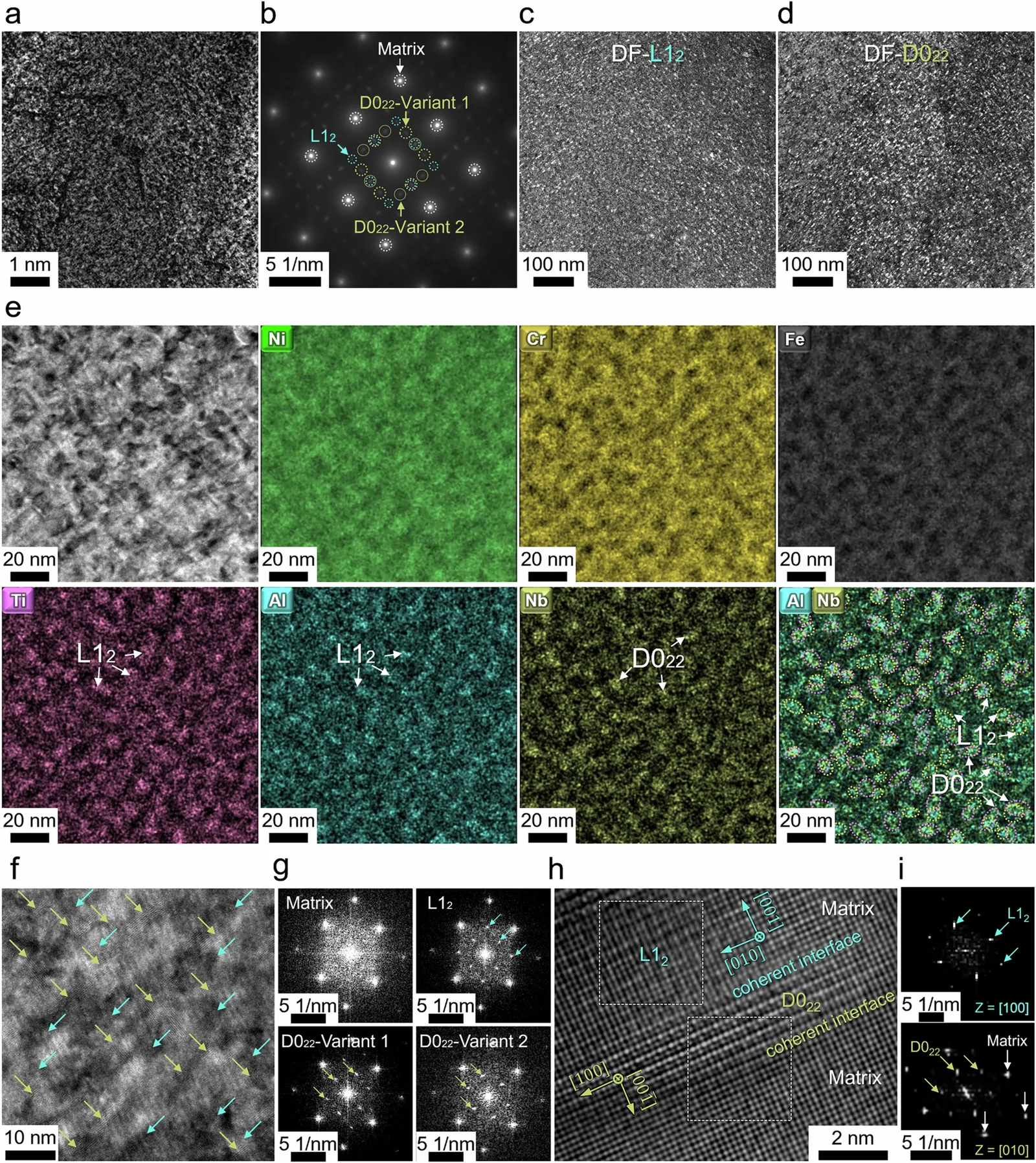
Structural analyses of coherent-dual-precipitate Inconel 718 nickel-based superalloy. **a**, Representative TEM image showing the microstructure of Inconel 718 nickel-based superalloy. **b**, SAED pattern of **a**, showing the characteristic diffraction spots of the L1_2_ precipitates (turquoise) and D0_22_ precipitates (green), which are distinct from the FCC matrix (white). **c**, Dark-field TEM (DF-TEM) image of L1_2_ phase, showing a high density of L1_2_ precipitates. **d**, DF-TEM image of D0_22_ phase, showing a high density of D0_22_ precipitates. **e**, High-resolution HAADF-STEM image and the corresponding EDS mapping, with the matrix elements (Ni, Cr and Fe), the L1_2_ precipitate elements (Ni, Al and Ti) and the D0_22_ precipitate elements (Ni and Nb). The L1_2_ and the D0_22_ precipitates are spatially connected dual precipitates. **f**, HRTEM image of the sequential precipitation of L1_2_ (turquoise) and D0_22_ (green) precipitates. **g**, FFT images of the FCC matrix, the L1_2_ and two variants of D0_22_ precipitates. **h**, HRTEM image of a single L1_2_–D0_22_ composite particle, showing the L1_2_–D0_22_ and matrix–D0_22_ coherent interfaces. **i**, FFT images of the coherent L1_2_ and D0_22_ precipitates.

With the establishment that APT can resolve hydrogen in different phases, we extended our study to a more complex alloy system containing several types of precipitate, which is an Inconel 718 nickel-based superalloy (54Ni–19Fe–21Cr–2.6Nb–1.6Mo–0.97Ti–0.58Al–0.24C at.%), by 3D printing and tailored it to contain two types of coherent precipitate—Ni_3_Nb body-centred tetragonal (BCT) D0_22_ and Ni_3_(Al, Ti) L1_2_ FCC—embedded in an FCC matrix. As shown in Extended Data Fig. 8, we conducted TEM analyses to confirm the presence of a high number density of precipitates in the Ni matrix, Ni–Al–Ti enrichment in the L1_2_ phase, Ni–Nb enrichment in the D0_22_ phase and the high coherency of L1_2_–D0_22_ and matrix–D0_22_ interfaces. This co-precipitation strategy enabled both phases of interest to appear within the small APT field of view, allowing a comparison under identical experimental conditions.

**Fig. 4:**
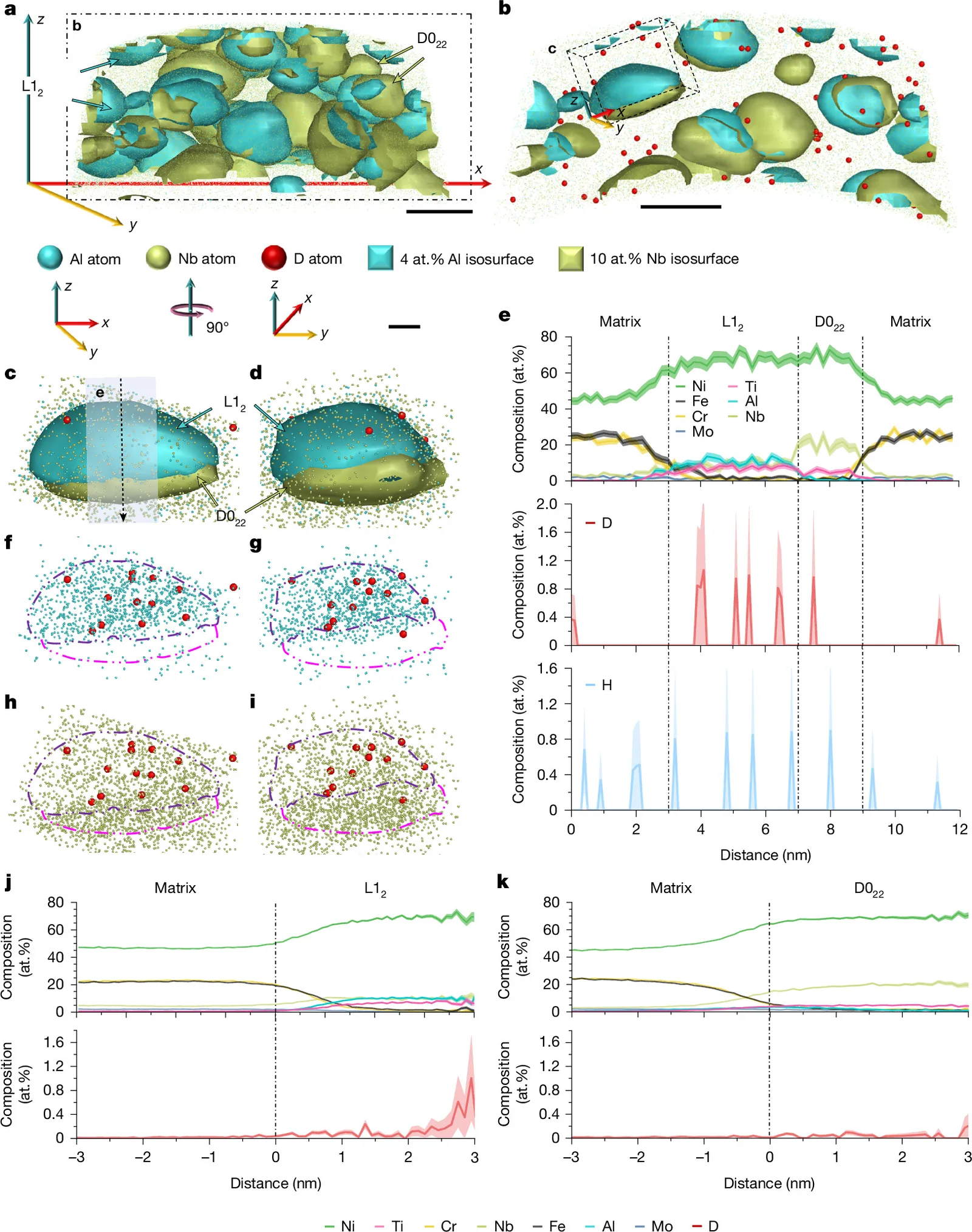
Voltage-pulsing APT results of deuterium-charged dual-precipitate Inconel 718 nickel-based superalloy. **a**, Three-dimensional reconstruction with 4 at.% Al (turquoise) and 10 at.% Nb (green) isosurfaces, as well as the location of the slice for **b**. **b**, 10-nm slice view of the Al and Nb isosurfaces, D atoms (not all shown owing to blockage of the isosurfaces) and the region of interest for **c**. **c**,**d**, Two viewing aspects of a dual precipitate of L1_2_ (turquoise) and D0_22_ (green) in three dimensions. **e**, One-dimensional concentration profile from the region of interest noted in **c**, showing the presence of D concentration primarily in the L1_2_ region, not the D0_22_ region, in contrast to the distribution of background hydrogen (blue). **f**,**g**, The Al (turquoise) and D atom maps, corresponding to **c** and **d**, respectively. **h**,**i**, The Nb (green) and D atom maps, corresponding to **c** and **d**, respectively. **j**, Cumulative proxigram from the Al isosurfaces shown in **a**, showing the D concentration in L1_2_ precipitates. **k**, Cumulative proxigram from the Nb isosurfaces shown in **a**, showing the lack of D in D0_22_ precipitates. Scale bars, 10 nm (**a**,**b**); 2 nm (**c**,**d**).

We performed identical treatment for the (CoCrNi)_94_Al_3_Ti_3_ specimen. The results are presented in Fig. 4. Figure 4a,b shows the spatially connected dual-precipitate region in three-dimensional reconstruction and a 10-nm slice, respectively. The Al-rich L1_2_ precipitates are identified in turquoise using 4 at.% Al isosurfaces, whereas the Nb-rich D0_22_ precipitates are identified in green using 10 at.% Nb isosurfaces. In Fig. 4c–i, we show a single L1_2_–D0_22_ composite particle from two viewing directions, highlighting the Al and Nb isosurfaces (Fig. 4c,d), the Al and D atoms (Fig. 4f,g) or the Nb and D atoms (Fig. 4h,i). The corresponding mass spectrum is shown in Supplementary Fig. 6.

**Extended Data Fig. 9:**
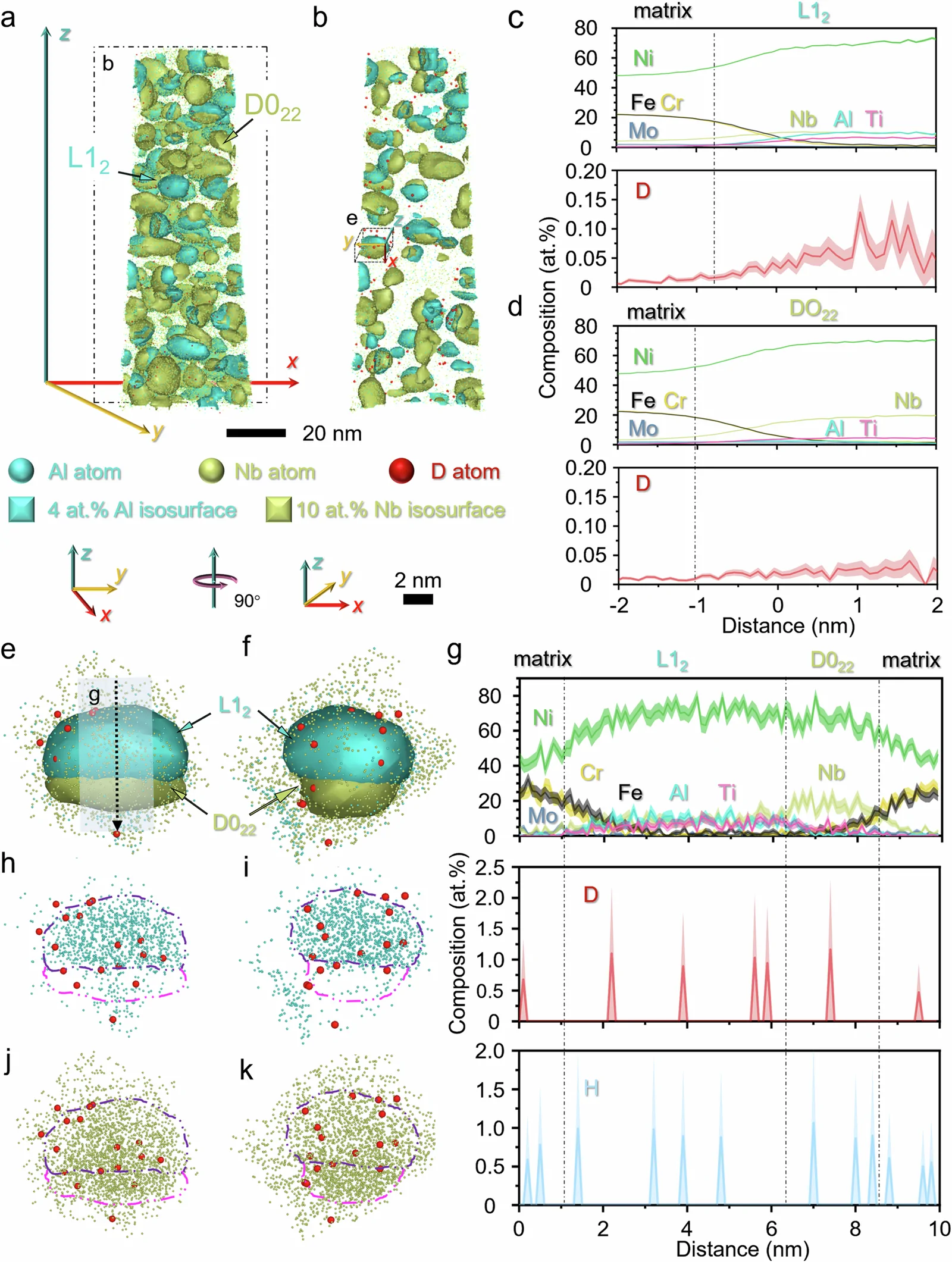
Reproduced voltage-pulsed APT of a deuterium-charged dual-precipitate Inconel 718 nickel-based superalloy corresponding to Fig. 4. **a**, Three-dimensional reconstruction showing 4 at.% Al (turquoise) and 10 at.% Nb (green) isosurfaces, along with the position of the slice shown in **b**. **b**, 10-nm-thick slices showing the Al and Nb isosurfaces, D atoms and the region of interest used in **e**. **c**, Cumulative proxigram from the Al isosurfaces in **a**, showing D enrichment in L1_2_ precipitates. **d**, Cumulative proxigram from the Nb isosurfaces in **a**, showing the lack of D in D0_22_ precipitates. **e**,**f**, Two three-dimensional viewing angles of a dual precipitate consisting of L1_2_ (turquoise) and D0_22_ (green). **g**, One-dimensional concentration profile taken from the region of interest in **e**, revealing that D is concentrated primarily in the L1_2_ region and not in the D0_22_ region, in contrast to the distribution of background hydrogen (blue). **h**,**i**, Al (turquoise) and D atom maps correspond to the views in **e** and **f**, respectively. **j**,**k**, Nb (green) and D atom maps correspond to the views in **e** and **f**, respectively.

As a result, D atoms were found predominantly within the L1_2_ precipitates, as confirmed by the one-dimensional concentration profile in Fig. 4e, which was obtained from the cylindrical region of interest indicated in Fig. 4c. To quantify this further, we acquired proxigrams using either the turquoise Al isosurfaces (representing L1_2_) or the green Nb isosurfaces (representing D0_22_), as shown in Fig. 4j,k, respectively. These profiles clearly show an increased D concentration in the L1_2_ precipitates (Fig. 4j) and only a low D concentration in the D0_22_ precipitates (Fig. 4k). We repeated the experiment on another Inconel APT tip and the data in Extended Data Fig. 9 and Supplementary Fig. 7 show the same trend, confirming reproducibility. We also conducted DFT analysis to determine the hydrogen solution energies in a pure Ni reference (Extended Data Fig. 2c), the Ni_2_FeCr Inconel FCC matrix (Extended Data Fig. 2d) and the Ni_3_Nb D0_22_ BCT precipitate (Extended Data Fig. 2e), finding that none of the sites in D0_22_ can hold hydrogen better than those in the L1_2_ precipitates, which is consistent with the APT results in Figs. 3 and 4.

In summary, we investigated interstitial hydrogen/deuterium in solid solutions of FCC MPEAs, in both bulk matrix and precipitate forms, by APT, with results consistent with DFT predictions of hydrogen solution energies. The deuterium partitioning reflects interstitial occupancy, although minor contributions from vacancies, vacancy–hydrogen/deuterium complexes or elastic fields cannot be fully excluded. Chemical affinity and crystal structure are shown to govern local hydrogen distribution. These findings establish an experimental protocol for connecting atomistic predictions with direct observation, enabling new opportunities for understanding hydrogen thermodynamics and kinetics in materials and providing guidance for designing alloys with improved performance for hydrogen-related applications.

## Methods

### Materials

The ingot of CoCrNiFeMn high-entropy alloy was prepared by vacuum arc melting a mixture of pure elements with purity greater than 99.95 wt.% under an argon atmosphere after a vacuum of 3.3 Pa has been reached. The ingot was melted at least four times to ensure compositional homogeneity, followed by homogenization at 1,200 °C for 24 h, cold rolling to a 90% thickness reduction (from 10 mm to 1 mm) and annealing at 950 °C for 4 h.

The sample with a nominal chemical composition of (CoCrNi)_94_Al_3_Ti_3_ (at.%) was synthesized through vacuum arc melting high-purity metals (purity ≥ 99.9 wt.%) under an argon atmosphere. To ensure compositional homogeneity and prevent element segregation, the sample was melted five times before being poured into a round ingot with a diameter of 50 mm. The as-cast sample was homogenized at 1,200 °C for 3 h and then cold-rolled along with its thickness to reduce the thickness by 50%. Two different microstructures were achieved by applying distinct heat treatments to the as-rolled samples: heating at 1,000 °C for 1 h, yielding the precipitate-free state (solution-treated), followed by 800 °C for 5 h, yielding the precipitated sample. All heat-treated samples were water-quenched.

The ingot of CoNiV alloy was synthesized by vacuum arc melting under an argon atmosphere (99.99% purity) after a pressure of 3.3 Pa. To ensure compositional homogeneity, the ingots were remelted five times. The ingots were then hot-forged at 1,200 °C into rectangular slabs with a thickness of 5 mm and a width of 20 mm, followed by homogenization at 1,200 °C for 3 h. Subsequently, the homogenized alloys were cold-rolled to a total thickness reduction of 50%. Finally, the cold-rolled sheets were subjected to solution annealing at 1,000 °C for 1 h (solution-treated).

The Inconel 718 nickel-based superalloy had a chemical composition of 54.72Ni–18.60Fe–18.70Cr–4.24Nb–2.62Mo–0.80Ti–0.27Al–0.05C wt.% (53.93Ni–19.26Fe–20.80Cr–2.64Nb–1.58Mo–0.97Ti–0.58Al–0.24C at.%). This alloy (diameter 15 mm, length 70 mm) was fabricated using a commercial selective laser melting system (EOS M 400) with an average powder size of 30 ± 11 µm, a scanning strategy of 67° rotation, a laser power of 340 W, scanning speed of 1,100 mm s^−1^ and layer thickness of approximately 50 µm. The printed samples were initially homogenized at 1,100 °C for 1 h and then water-quenched to room temperature. Subsequently, the samples were subjected to a two-step ageing treatment: first at 720 °C for 1 h, followed by furnace cooling to 620 °C, at which they were held for 8 h before final air cooling to room temperature. The sequential D0_22_ (γ″-type) and L1_2_ (γ′-type) precipitates are introduced during the 720 °C and 620 °C ageing treatments, respectively.

### Electron microscopy

Samples for the EBSD analysis were initially polished using 5000-grit SiC sandpaper, subsequently polished with diamond paste for 3 min and finally electrolytically polished in a 1:9 solution of HClO_4_ and C_2_H_5_OH at 243 K for 20 s under a DC voltage of 25 V. The pre-thinned TEM foils, with a thickness of 50–60 μm, were polished using double-jet electropolishing at 243 K in 10 volume percent of HClO_4_ in methanol to prepare the TEM sample.

EBSD mapping used a Symmetry S2 detector (Oxford Instruments) and TEM imaging was conducted on a JEM-2100F (JEOL). High-resolution STEM imaging was performed using the FEI Themis Z microscope equipped with an EDS detector. Atomic-resolution chemical characterization with compositional mapping was performed using four in-column Super-X silicon drift detectors.

### Atom probe tomography

The sample preparation began with matchstick bars with dimensions 1 × 1 × 13 mm^3^. The samples were initially polished using 800-grit sandpaper. Coarse electropolishing was then performed using a 25% perchloric acid solution in glacial acetic acid at voltages ranging from 15 to 30 V to polish the samples into needle-like specimens. Fine electropolishing was subsequently performed using a 2% perchloric acid solution in ethylene glycol butyl ether at voltages ranging from 10 to 25 V. This fine electropolishing procedure used a platinum wire ring cathode and was monitored under a 40× optical microscope. A key step involved forming a neck until the top portion detached, creating a sharp tip.

Before deuterium charging, FIB tip sharpening was performed on the electropolished tips to ensure that the samples satisfied APT requirements in tip sharpness. APT was then performed using a LEAP 4000X instrument (CAMECA), operating in voltage-pulsing mode with a 0.2% detection rate, 200-kHz pulse frequency, 20% pulse fraction and sample temperature of 50 K. Data processing was performed using AP Suite software (version 6.1, CAMECA). The image compression factors and *k* factors used for reconstruction were 1.3 and 3.3 for the CoCrNiFeMn alloy and the Inconel 718 nickel-based superalloy and 1.5 and 3.3 for the (CoCrNi)_94_Al_3_Ti_3_ alloy, respectively. These reconstruction parameters were selected following standard APT reconstruction procedures and, for the precipitate-containing datasets, refined by comparison with precipitate morphologies independently observed by TEM. Peak ranging was performed by considering the full isotopic distributions of the major alloying elements and possible molecular ions; the bulk composition analysis is shown in Supplementary Tables 2–4. A peak-free region around 2.5 Da was defined as noise and used to estimate the local low-mass background adjacent to the 2-Da signal. Three representative mass spectra are shown in Supplementary Fig. 8 and the results for all eight successful cryo-APT datasets out of 68 trials are summarized in Supplementary Table 5. Because D^+^ and H_2_^+^ overlap at 2 Da and cannot be resolved directly, this overlap was treated separately as a systematic uncertainty and assessed using uncharged reference specimens analysed under identical voltage-pulsing conditions. APT reconstruction sensitivity was further evaluated by reconstructing the dataset shown in Fig. 3 using an alternative image compression factor value of 1.3, which did not alter the observed preferential deuterium enrichment within the L1_2_ precipitate (Supplementary Fig. 9).

### Electrolytic deuterium charging

A charging solution of 0.1 M NaOH in D_2_O (Sigma-Aldrich) was used to create a deuterium-rich environment around the samples at the cathode in an electrolytic circuit, with a gold counter-electrode. This was carried out in a custom-designed cryo-transfer glovebox (M. BRAUN Inertgas-Systeme). Four sharp specimens were pre-mounted in a four-needle atom probe sample puck with polyether ether ketone insulation. The four tips protruded from the puck to the same height and were immersed into the charging solution at a depth that was barely visible and could only be identified by means of the capillary of the liquid solution. Deuterium charging was conducted at 1.6 V for 90 s powered by Keysight 2901A, followed by immediately immersing the whole puck in a LN_2_ bath.

### Cryogenic sample transfer and cryo-FIB for APT specimens

After deuterium charging and LN_2_ quenching, the specimens for Figs. 3 and 4 were transferred from the glovebox to a Hydra plasma focused ion beam (PFIB; Thermo Fisher Scientific) with a cryo-stage using a cryogenic sample carrier (Ferrovac). The sharpening process was completed on a cryo-stage at approximately −190 °C. Two ion-beam conditions were used—30 kV–0.1 nA and 30 kV–30 pA—to remove ice and further refine and smooth the tips during the annular milling process. A final polishing step at 5 kV–50 pA was applied for 10 s to remove the possible ion-damaged layer. The APT specimens were then transferred using the cryogenic transfer suitcase from the PFIB system to the APT system for APT measurements.

### Thermal desorption spectroscopy

For the TDS tests, samples measuring 1 × 8 × 10 mm^3^ were polished using 5000-grit SiC sandpaper. The samples were then charged for 72 h at a constant current density of 20 mA cm^−^^2^ in a solution containing 0.5 M H_2_SO_4_ and 2 g l^−1^ CH_4_N_2_S. TDS measurements were subsequently performed using a mass spectrometer (JTF-20A), with heating rates of 100 °C h^−1^, 200 °C h^−1^ and 300 °C h^−1^.

### Density functional theory calculations

All DFT calculations were performed using the projector augmented wave^39^ method, as implemented in the Vienna Ab initio Simulation Package (VASP)^40^. The exchange-correlation effects were selected as the generalized gradient approximation determined by the Perdew–Burke–Ernzerhof functional^41^. To simulate chemical disorder, the special quasirandom structure (SQS) approach^42^ was applied using the Alloy Theoretic Automated Toolkit (ATAT)^43,44^. A plane-wave cut-off of 450 eV was used and the Brillouin zones were sampled with a gamma-centred Monkhorst–Pack grid^45^. Next, the Methfessel–Paxton method^46^ was used with a smearing width of 0.1 eV. The supercell size and atomic positions were allowed to relax until the Hellmann–Feynman forces were less than 0.01 eV Å^−1^ and convergence criteria for energies and forces were set to 1.0 × 10^−5^ eV per atom and 0.02 eV Å^−1^, respectively.

CoCrNiFeMn MPEA, CoCrNi MPEA and L1_2_ Ni_3_(Al, Ti) precipitates, pure Ni, Ni_2_FeCr (matrix of Inconel 718 superalloy) and D0_22_ Ni_3_Nb precipitates were studied in this work. The CoCrNiFeMn, CoCrNi, L1_2_ Ni_3_(Al, Ti), Ni and Ni_2_FeCr are all FCC structures and D0_22_ Ni_3_Nb has a tetragonal structure (I4/mmm, space group 139), the Ni atoms occupy the 4d (0, 1/2, 1/4) and 2b (0, 0, 1/2) positions and the Nb atoms occupy the 2a (0, 0, 0) positions. The SQS method was used to model the disordered phases including CoCrNiFeMn, CoCrNi, L1_2_ Ni_3_(Al, Ti) and Ni_2_FeCr. The CoCrNi, Ni_2_FeCr, L1_2_ Ni_3_(Al, Ti) and Ni phases were modelled using 108 atoms with 3 × 3 × 3 supercells according to their compositions, with all of the Ni atoms occupying the face-centred sites in L1_2_ Ni_3_(Al, Ti). CoCrNiFeMn and D0_22_ Ni_3_Nb were constructed by a 5 × 3 × 2 supercell containing 120 atoms and a 2 × 2 × 1 supercell containing 36 atoms, respectively. The CoCrNi ($1\bar{1}\bar{1}$)/L1_2_ Ni_3_(Al, Ti) ($1\bar{1}\bar{1}$) interface was constructed on the basis of experimental data and more than three SQS configurations for each bulk and interface structure were evaluated for comparison.

The DFT analysis mainly considered both octahedral interstitial and tetrahedral interstitial sites. The solution energy (*E*_s_) of a hydrogen atom at a trapping site was calculated using equation (1):

1 $${E}_{{\rm{s}}}={E}_{{\rm{trap}}+{\rm{H}}}-{E}_{{\rm{trap}}}-1/2{E}_{{{\rm{H}}}_{2}}$$

in which *E*_trap+H_ and *E*_trap_ denote the total energies of the system with and without an interstitial hydrogen atom, respectively, and $1/2{E}_{{{\rm{H}}}_{2}}$ denotes the half energy of a H_2_ molecule. Given the heterogeneous chemical environment surrounding the interstitial sites in MPEAs, more than 20 randomly selected interstitial sites were sampled in each bulk phase structure, along with ten randomly selected interstitial sites at each interface structure, including both octahedral interstitial sites and tetrahedral interstitial sites.

We performed further calculations about the formation of vacancy. We selected three representative phases (CoCrNi, L1_2_ Ni_3_(Al, Ti) and D0_22_ Ni_3_Nb) to conduct the relevant investigations, which can reflect the general energetic characteristics. Vacancies were introduced by removing atoms from the perfect supercell, the vacancy formation energy ${E}_{{\rm{V}}}^{{\rm{f}}}$ was computed as follows:

2 $${E}_{{\rm{V}}}^{{\rm{f}}}={E}_{{\rm{V}}}-{E}_{{\rm{bulk}}}+{\mu }_{i}$$

in which *E*_V_ and *E*_bulk_ are the energies of defective and defect-free supercells, respectively, and *μ*_*i*_ is the chemical potential of the removed atom from the supercell. The chemical potential of the vacancy atom *i* was calculated according to the Widom-type substitution technique^47^. We calculated 40 substituted structures to determine the *μ*_*i*_ of each element *i*. The calculated chemical potentials of different elements in CoCrNi were used to calculate the formation energies of vacancies in CoCrNi. For L1_2_ Ni_3_(Al, Ti) and D0_22_ Ni_3_Nb, *μ*_*i*_ denotes the total energy of the X atom at its ground state.

According to quantum mechanics, particles maintain a residual non-zero energy even at 0 K, which is defined as zero-point energy (ZPE)^48^. Here we used the ZPE and entropy corrections on H solution energies for three of the investigated alloys (CoCrNi, L1_2_ Ni_3_(Al, Ti) and D0_22_ Ni_3_Nb). The Gibbs free energy of hydrogen dissolution, Δ*G*_s_ is defined as follows:

3 $$\Delta {G}_{{\rm{s}}}={E}_{{\rm{s}}}+\Delta {E}_{{\rm{ZPE}}}-T\Delta S$$

in which *E*_s_ is the calculated solution energy, Δ*E*_ZPE_ is the difference in ZPE of hydrogen in the dissolved state and the gas phase and Δ*S* is the entropy difference between the dissolved state and the gas phase of hydrogen at standard conditions (atmospheric pressure, 298 K). The calculated ZPE of a diatomic H_2_ gas is 0.269 eV, which is in good agreement with previous studies^48–50^. Supplementary Fig. 10 illustrates the comparison of energy results with and without ZPE and entropy corrections. The energy slightly increases after ZPE correction is applied and the increase of tet sites is marginally larger than the oct sites for CoCrNi and Ni_3_(Al, Ti). The energy increases further with more entropy corrections for all three alloys. It can be seen that the ZPE and entropy corrections exert no notable influence on the energy comparison.

**Extended Data Fig. 10:**
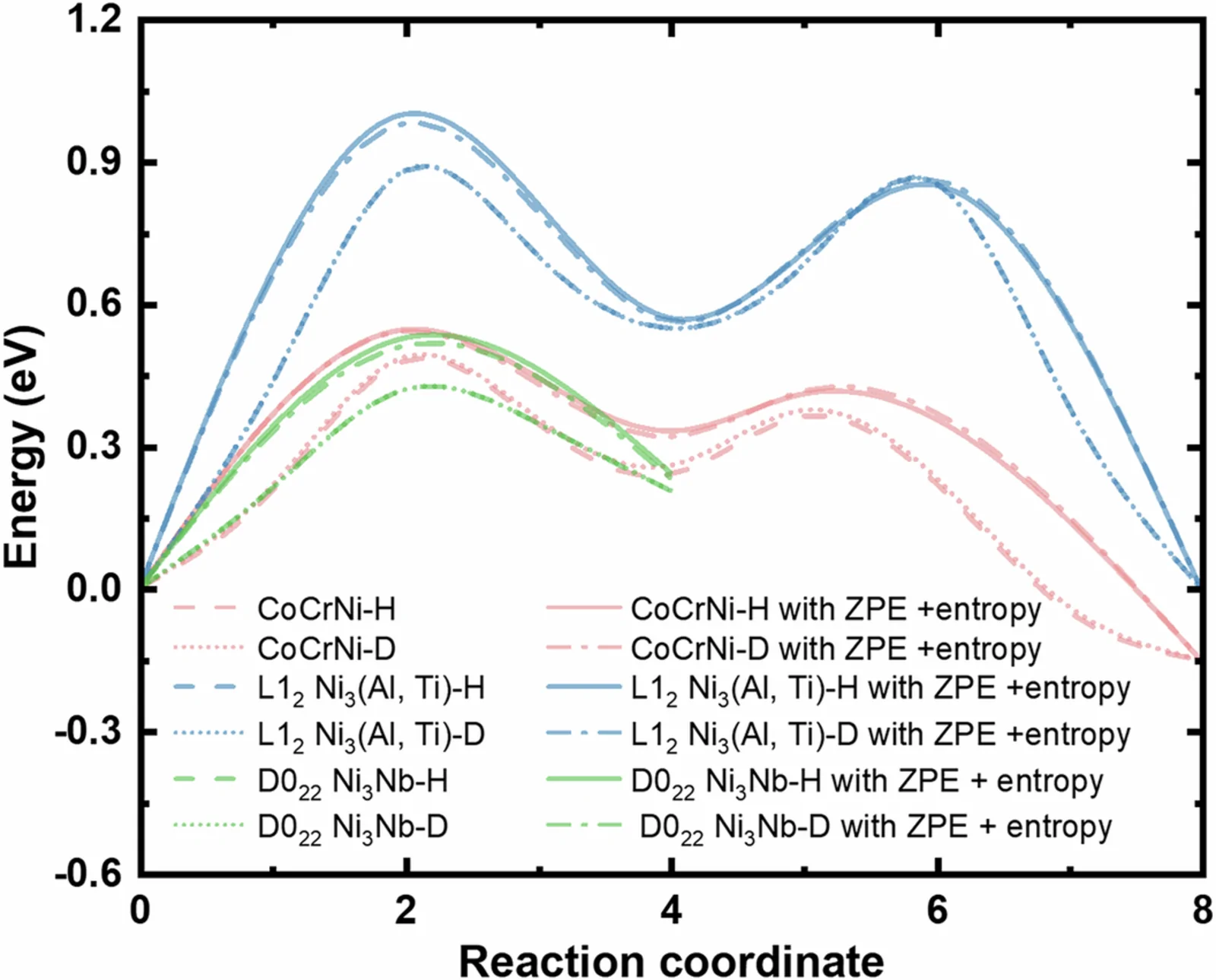
Hydrogen and deuterium diffusion pathways. Calculated H and D diffusion energy profiles along the reaction coordinate in CoCrNi (pink), L1_2_ Ni_3_(Al, Ti) (blue) and D0_22_ Ni_3_Nb (green). Profiles are shown before and after inclusion of ZPE and entropy corrections.

Isotopic effect was also considered for these alloys (CoCrNi, L1_2_ Ni_3_(Al, Ti) and D0_22_ Ni_3_Nb). H atom was substituted by D atom and the calculated results are shown in Supplementary Fig. 11. Replacing H with D exerts a negligible influence on the solution energies for all three phases. The energy slightly increases with the ZPE correction, which is similar to the case for H. However, the general trend of energy comparison is insensitive to isotopic effects. The diffusion of H/D atoms within the three phases (CoCrNi, L1_2_ Ni_3_(Al, Ti) and D0_22_ Ni_3_Nb) was investigated using the climbing-image nudged elastic band method. As shown in Extended Data Fig. 10, the diffusion barriers of H and D in the three phases are similar to each other. After applying the corrections, the saddle points of the diffusion curves of all three phases (CoCrNi, L1_2_ Ni_3_(Al, Ti) and D0_22_ Ni_3_Nb) shift upward slightly, with minor differences observed between H and D. The general energetic trend remains the same after the corrections.

#### Quasi in situ TEM observation during electrochemical deuterium charging

Electropolished TEM foils of the aged (CoCrNi)_94_Al_3_Ti_3_ alloy were used for quasi in situ D-charging experiments. Before charging, specific regions containing L1_2_ precipitates were identified and characterized using bright-field TEM, high-angle annular dark-field scanning transmission electron microscopy (HAADF-STEM), selected-area electron diffraction (SAED) and HRTEM. The specimen was then electrochemically charged in 0.1 M NaOH prepared in D_2_O using a gold anode. A charging time of 1.5 min and the charging conditions used are the same as cryogenic APT experiments.

After charging, the specimen was removed from the electrolyte, dried and re-examined by TEM. The same regions and individual precipitates were relocated and compared with their former states. SAED and HRTEM analyses were performed after charging to assess whether D-charging introduced crystalline defects, including stacking faults, dislocations or other lattice defects.

#### Ion irradiation and positron annihilation spectroscopy

Ion irradiation was performed using a NEC 2 × 1.7 MV tandem accelerator at Peking University. The (CoCrNi)_94_Al_3_Ti_3_ specimen (10 × 10 × 0.9 mm^3^) was irradiated with 3 MeV Ni^2+^ ions to a fluence of 1.5 × 10^16^ ions cm^−2^ at 500 °C, corresponding to a peak damage level of approximately 16 dpa, as calculated by SRIM-2013 using the quick Kinchin–Pease model. The displacement threshold energies used in the SRIM calculations were 40 eV for Co, Cr, Ni and Ti and 25 eV for Al. To minimize oxidation and other possible surface contamination during high-temperature irradiation, the target chamber was evacuated to a pressure below 1 × 10^−4^ Pa before heating and irradiation were initiated. After irradiation, the specimen was kept under vacuum during cooling and removed from the chamber only after the temperature had decreased to less than 50 °C.

Positron annihilation lifetime spectroscopy was performed using a ^22^Na positron source to complement the Doppler-broadening measurements and assess vacancy-type defects in the investigated alloys, corresponding to Supplementary Table 1. Vacancy-type defects introduced by hydrogen charging, performed under the same conditions as those used for the cryogenic APT experiments, and by ion irradiation were characterized by Doppler-broadening positron annihilation spectroscopy using a slow positron beam^51,52^ at the Institute of High Energy Physics, Chinese Academy of Sciences. A ^22^Na radioactive source was used to produce the positron beam, with a beam diameter of approximately 0.55 cm. To minimize specimen-to-specimen variability, Doppler-broadening positron annihilation spectroscopy measurements were performed sequentially on the same specimen in the pristine state, after electrochemical hydrogen charging and after subsequent Ni-ion irradiation. The incident positron energy varied from 0.5 to 25.0 keV. The corresponding mean positron implantation depth ranged from approximately 1.5 to 804.0 nm (corresponding to the irradiated region spanning approximately 5.8–15.3 dpa), as estimated using the empirical relation:

4 $$Z=\frac{40}{\rho }{E}^{1.6}$$

in which *Z* is the mean positron implantation depth in nm, *ρ* is the mass density of the target material in g cm^−3^ and *E* is the incident positron energy in keV. A density of 8.58 g cm^−3^ was used for the (CoCrNi)_94_Al_3_Ti_3_ alloy. Positron annihilation γ-ray spectra were recorded using a high-purity Ge detector. For each spectrum, more than 4.5 × 10^5^ counts were accumulated within the 499.5–522.5 keV annihilation peak. The Doppler-broadening spectra were analysed using the S and W parameters, which represent positron annihilation with low-momentum valence electrons and high-momentum core electrons, respectively. The annihilation peak was integrated over the energy range 499.5–522.5 keV. The S parameter was defined as the ratio of the counts in the central energy window of 510.2–511.8 keV to the total counts in the 499.5–522.5 keV annihilation peak. The W parameter was defined as the ratio of the counts in the high-momentum wing regions of 505.1–508.4 keV and 513.6–516.9 keV to the total counts in the 499.5–522.5 keV annihilation peak. An increase in S accompanied by a decrease in W indicates enhanced positron trapping at vacancy-type open-volume defects.

## Online content

Any methods, additional references, Nature Portfolio reporting summaries, source data, extended data, supplementary information, acknowledgements, peer review information; details of author contributions and competing interests; and statements of data and code availability are available at https://doi.org/10.1038/s41586-026-11238-4.

## Supplementary information

Supplementary Information Supplementary Figs. 1–11 and Supplementary Tables 1–5.

Peer Review File

## Data Availability

All data in this study are available at http://github.com/huangchaohitsz-crypto/Source-Data_Toward-observing-interstitial-hydrogen-in-solution-in-metal-lattices.

## References

[1] Boretti, A. & Pollet, B. G. Hydrogen economy: paving the path to a sustainable, low-carbon future. *Int. J. Hydrog. Energy* **93**, 307–319 10.1016/j.ijhydene.2024.10.350 (2024).

[2] Chen, Y.-S. et al. Hydrogen trapping and embrittlement in metals – a review. *Int. J. Hydrog. Energy* **136**, 789–821 10.1016/j.ijhydene.2024.04.076 (2025).

[3] Chen, Y. S. et al. Observation of hydrogen trapping at dislocations, grain boundaries, and precipitates. *Science* **367**, 171–175 10.1126/science.aaz0122 (2020). 31919217

[4] Chen, Y.-S. et al. Atom probe tomography for the observation of hydrogen in materials: a review. *Microsc. Microanal.* **29**, 1–15 10.1093/micmic/ozac005 (2023).

[5] Koyama, M. et al. Recent progress in microstructural hydrogen mapping in steels: quantification, kinetic analysis, and multi-scale characterisation. *Mater. Sci. Technol.* **33**, 1481–1496 10.1080/02670836.2017.1299276 (2017).

[6] Gault, B. et al. Towards establishing best practice in the analysis of hydrogen and deuterium by atom probe tomography. *Microsc. Microanal.* **30**, 1205–1220 10.1093/mam/ozae081 (2025). 39226242

[7] Nagumo, M. *Fundamentals of Hydrogen Embrittlement* (Springer, 2016).

[8] Pundt, A. & Kirchheim, R. Hydrogen in metals: microstructural aspects. *Annu. Rev. Mater. Res.* **36**, 555–608 10.1146/annurev.matsci.36.090804.094451 (2006).

[9] Andresen, A. F. & Maeland, A. Hydrides for energy storage. In Proc. International Symposium Held in Geilo, Norway, 14–19 August 1977 (eds Andresen, A. F. & Maeland, A. J.) (Elsevier, 1978).

[10] Jia, Y.-J. & Han, W.-Z. Mechanisms of hydride nucleation, growth, reorientation, and embrittlement in zirconium: a review. *Materials* **16**, 2419 10.3390/ma16062419 (2023). PMC10059671 36984297

[11] Rethinasabapathy, M. et al. Recent progress in functional nanomaterials towards the storage, separation, and removal of tritium. *Adv. Mater.* **35**, e2301589 10.1002/adma.202301589 (2023). 37435972

[12] Chang, Y. et al. Ti and its alloys as examples of cryogenic focused ion beam milling of environmentally-sensitive materials. *Nat. Commun.* **10**, 942 10.1038/s41467-019-08752-7 (2019). PMC6391424 30808943

[13] Chang, Y. H. et al. Characterizing solute hydrogen and hydrides in pure and alloyed titanium at the atomic scale. *Acta Mater.* **150**, 273–280 10.1016/j.actamat.2018.02.064 (2018).

[14] Fukai, Y. Formation of superabundant vacancies in M–H alloys and some of its consequences: a review. *J. Alloys Compd.* **356**, 263–269 10.1016/S0925-8388(02)01269-0 (2003).

[15] Saksena, A. et al. Optimizing site-specific specimen preparation for atom probe tomography by using hydrogen for visualizing radiation-induced damage. *Int. J. Hydrog. Energy* **50**, 165–174 10.1016/j.ijhydene.2023.09.057 (2024).

[16] Huang, C. et al. Strong hydrogen trapping by tangled dislocations in cold-drawn pearlitic steels. *Acta Mater.* **296**, 121231 10.1016/j.actamat.2025.121231 (2025).

[17] Zhao, H. et al. Hydrogen trapping and embrittlement in high-strength Al alloys. *Nature* **602**, 437–441 10.1038/s41586-021-04343-z (2022). PMC8850197 35173345

[18] Niu, R. M. et al. Hydrogen-enhanced deformation in pearlite. *Acta Mater.* **281**, 120327 10.1016/j.actamat.2024.120327 (2024).

[19] Gault, B., Moody, M. P., Cairney, J. M. & Ringer, S. P. Atom probe crystallography. *Mater. Today* **15**, 378–386 10.1016/s1369-7021(12)70164-5 (2012).

[20] Takahashi, J., Kawakami, K., Kobayashi, Y. & Tarui, T. The first direct observation of hydrogen trapping sites in TiC precipitation-hardening steel through atom probe tomography. *Scr. Mater.* **63**, 261–264 10.1016/j.scriptamat.2010.03.012 (2010).

[21] Zhou, Z. Y. et al. Cryogenic atom probe tomography and its applications: a review. *Microstructures* **3**, 2023043 10.20517/microstructures.2023.38 (2023).

[22] Vukkum, V. B. et al. Insights from quasi-in situ cryogenic-transfer atom probe tomography for analyzing hydrogen diffusion in metallic alloys. *npj Mater. Degrad.* **9**, 85 10.1038/s41529-025-00626-2 (2025).

[23] Fukai, Y. Superabundant vacancies formed in metal–hydrogen alloys. *Phys. Scr.* **T103**, 11–14 10.1238/Physica.Topical.103a00011 (2003).

[24] Turnbull, A. & Hutchings, R. Analysis of hydrogen atom transport in a two-phase alloy. *Mater. Sci. Eng. A* **177**, 161–171 (1994).

[25] Cantor, B., Chang, I. T. H., Knight, P. & Vincent, A. J. B. Microstructural development in equiatomic multicomponent alloys. *Mater. Sci. Eng. A* **375**, 213–218 10.1016/j.msea.2003.10.257 (2004).

[26] Belo, J. S. et al. Hydrogen diffusivity and interaction with Fe_20_Mn_20_Ni_20_Co_20_Cr_20_ and Fe_22_Mn_40_Ni_30_Co_6_Cr_2_ high-entropy alloys. *J. Alloys Compd.* **815**, 152314 10.1016/j.jallcom.2019.152314 (2020).

[27] Gault, B., Moody, M. P., Cairney, J. M. & Ringer, S. P. *Atom Probe Microscopy* (Springer, 2012).

[28] Fukai, Y. *The Metal–Hydrogen System. Basic Bulk Properties* (Springer, 2005).

[29] Zhao, Y. L. et al. Heterogeneous precipitation behavior and stacking-fault-mediated deformation in a CoCrNi-based medium-entropy alloy. *Acta Mater.* **138**, 72–82 10.1016/j.actamat.2017.07.029 (2017).

[30] Xia, D. D. et al. Effect of NiAl content on microstructure and properties of (NiAl)_x_(CoCrFeNi)_94-x_Ta_6_ alloy. *Intermetallics* **183**, 108796 10.1016/j.intermet.2025.108796 (2025).

[31] Cheng, H. et al. The influence of L1_2_ ordered precipitates on hydrogen embrittlement behavior in CoCrNi-based medium entropy alloys. *Acta Mater.* **260**, 119328 10.1016/j.actamat.2023.119328 (2023).

[32] Nagumo, M. Hydrogen related failure of steels – a new aspect. *Mater. Sci. Technol.* **20**, 940–950 10.1179/026708304225019687 (2004).

[33] Hermida, J. D. & Roviglione, A. Stacking fault energy decrease in austenitic stainless steels induced by hydrogen pairs formation. *Scr. Mater.* **39**, 1145–1149 10.1016/S1359-6462(98)00285-1 (1998).

[34] Li, Q.-J., Sheng, H. & Ma, E. Strengthening in multi-principal element alloys with local-chemical-order roughened dislocation pathways. *Nat. Commun.* **10**, 3563 10.1038/s41467-019-11464-7 (2019). PMC6687833 31395881

[35] An, X. et al. Investigation of the interaction between hydrogen and irradiation defects in titanium by using positron annihilation spectroscopy. *Int. J. Hydrog. Energy* **46**, 13162–13170 10.1016/j.ijhydene.2021.01.128 (2021).

[36] Jena, P., Ponnambalam, M. J. & Manninen, M. Positron annihilation in metal-vacancy—hydrogen complexes. *Phys. Rev. B* **24**, 2884–2887 10.1103/PhysRevB.24.2884 (1981).

[37] Kirchheim, R. Reducing grain boundary, dislocation line and vacancy formation energies by solute segregation. I. Theoretical background. *Acta Mater.* **55**, 5129–5138 10.1016/j.actamat.2007.05.047 (2007).

[38] London, A. J. Quantifying uncertainty from mass-peak overlaps in atom probe microscopy. *Microsc. Microanal.* **25**, 378–388 10.1017/s1431927618016276 (2019). 30761977

[39] Blochl, P. E. Projector augmented-wave method. *Phys. Rev. B* **50**, 17953–17979 10.1103/physrevb.50.17953 (1994). 9976227

[40] Kresse, G. & Furthmuller, J. Efficient iterative schemes for *ab initio* total-energy calculations using a plane-wave basis set. *Phys. Rev. B* **54**, 11169–11186 10.1103/physrevb.54.11169 (1996). 9984901

[41] Perdew, J. P., Burke, K. & Ernzerhof, M. Generalized gradient approximation made simple. *Phys. Rev. Lett.* **77**, 3865–3868 10.1103/PhysRevLett.77.3865 (1996). 10062328

[42] Zunger, A., Wei, S., Ferreira, L. G. & Bernard, J. E. Special quasirandom structures. *Phys. Rev. Lett.* **65**, 353–356 10.1103/PhysRevLett.65.353 (1990). 10042897

[43] van de Walle, A. et al. Efficient stochastic generation of special quasirandom structures. *Calphad* **42**, 13–18 10.1016/j.calphad.2013.06.006 (2013).

[44] van de Walle, A., Asta, M. & Ceder, G. The alloy theoretic automated toolkit: a user guide. *Calphad* **26**, 539–553 10.1016/s0364-5916(02)80006-2 (2002).

[45] Monkhorst, H. J. & Pack, J. D. Special points for Brillouin-zone integrations. *Phys. Rev. B* **13**, 5188–5192 10.1103/PhysRevB.13.5188 (1976).

[46] Methfessel, M. & Paxton, A. T. High-precision sampling for Brillouin-zone integration in metals. *Phys. Rev. B* **40**, 3616–3621 10.1103/physrevb.40.3616 (1989). 9992329

[47] Luo, Z., Gao, W. & Jiang, Q. Determinants of vacancy formation and migration in high-entropy alloys. *Sci. Adv.* **11**, eadr4697 10.1126/sciadv.adr4697 (2025). PMC11698082 39752502

[48] Setyawan, W. & Senor, D. J. Influence of hydrogen isotopes on vacancy formation and antisite defect diffusion in palladium and vanadium metals. *Comput. Mater. Sci.* **197**, 110641 10.1016/j.commatsci.2021.110641 (2021).

[49] Counts, W. A., Wolverton, C. & Gibala, R. First-principles energetics of hydrogen traps in α-Fe: point defects. *Acta Mater.* **58**, 4730–4741 10.1016/j.actamat.2010.05.010 (2010).

[50] Jiang, D. E. & Carter, E. A. Diffusion of interstitial hydrogen into and through bcc Fe from first principles. *Phys. Rev. B* **70**, 064102 10.1103/PhysRevB.70.064102 (2004).

[51] Li, C. et al. Implantation profiles and depth distribution of slow positron beam simulated by Geant4 toolkit. *Phys. Scr.* **94**, 045301 10.1088/1402-4896/aafaa2 (2019).

[52] Yu, R. S., Maekawa, M., Kawasuso, A., Wang, B. Y. & Wei, L. Positron annihilation study of 4H-SiC by Ge^+^ implantation and subsequent thermal annealing. *Nucl. Instrum. Methods Phys. Res. B* **270**, 47–49 10.1016/j.nimb.2011.10.006 (2012).

