# Supplementary material for: Towards observing interstitial hydrogen in solution in metal lattices
Source: Nature. 2026 Nov 4;659(8140):357–64. doi: 10.1038/s41586-026-11238-4 (PMC13754584; doi:10.1038/s41586-026-11238-4)
Supplement: Supplementary file 1 [file 41586_2026_11238_MOESM1_ESM.pdf]

nature portfolio

https://doi.org/10.1038/s41586-026-11238-4

Supplementary information

Towards observing interstitial hydrogen in solution in metal lattices

In the format provided by the
authors and unedited

Supplementary Information for

Toward observing interstitial hydrogen in solution in metal lattices

Chao Huang[1,2,3,†], Saiyu Liu[1,†], Wenting Lv[4,†], Chun Chen[2,3,†], Shangkun Shen[5,†], Ranming Niu[2,3,*], Zhao Xu[1], Yanqi Tu[1], Siwen Shen[5,6,7], Cheng-Yun Wu[2], Pang-Yu Liu[2,3], Kewei Gao[1], Yu Yang[4], Hung-Wei Yen[8,9], Xinping Mao[6], Engang Fu[10], Julie Cairney[2,3,*], Xiaolu Pang[1,*], Rongjian Shi[1,*], and Eason Yi-Sheng Chen[2,5,7,*]

[1] School of Materials Science and Engineering, State Key Laboratory of Nuclear Power Safety Technology and Equipment, University of Science and Technology Beijing, Beijing, 100083, China

[2] Australian Centre for Microscopy and Microanalysis, The University of Sydney, NSW 2006, Australia

[3] School of Aerospace Mechanical and Mechatronic Engineering, The University of Sydney, NSW 2006, Australia

[4] National Key Laboratory of Computational Physics, Institute of Applied Physics and Computational Mathematics, 100088, Beijing, China

[5] School of Materials Science and Engineering, Nanyang Technological University, Singapore, 639798

[6] Institute for Carbon Neutrality, University of Science and Technology Beijing, Beijing, 100083, China

[7] Energy Research Institute @ NTU, Nanyang Technological University, Singapore, 637141

[8] Department of Materials Science and Engineering, National Taiwan University, Taipei, 10617, Taiwan

[9] High Entropy Materials Center, National Tsing Hua University, Hsinchu, 30013, Taiwan

[10] State Key Laboratory of Nuclear Physics and Technology, School of Physics, Peking University, Beijing, 100871, China

[†] The authors contribute equally to this work.

[*] Corresponding authors: eason.chen@ntu.edu.sg; rongjianshi@ustb.edu.cn; pangxl@mater.ustb.edu.cn; julie.cairney@sydney.edu.au; ranming.niu@sydney.edu.au

Content

Supplementary Fig. 1 | Voltage-pulsing APT data of a D-charged single-phase $(CoCrNi)_{94}Al_3Ti_3$ specimen 3

Supplementary Fig. 2 | Electron backscatter diffraction analyses of the FCC $(CoCrNi)_{94}Al_3Ti_3$ specimens before and after ageing at 800 °C for 5 hours 4

Supplementary Fig. 3 | Voltage-pulsing atom probe tomography data of deuterium-free single-phase $(CoCrNi)_{94}Al_3Ti_3$ alloy corresponding to Fig. 2 5

Supplementary Fig. 4 | Thermal desorption spectroscopy results from $(CoCrNi)_{94}Al_3Ti_3$ specimens with various heating rate 5

Supplementary Fig. 5 | Vacancy formation energies in CoCrNi and $L1_2$ $Ni_3(Al, Ti)$ 6

Supplementary Fig. 6 | Mass spectrum of the D-charged, dual-precipitate Inconel 718 nickel-based superalloy 6

Supplementary Fig. 7 | Mass spectrum of the D-charged, dual-precipitate Inconel 718 nickel-based superalloy 7

Supplementary Fig. 8 | Assessment of peak ranging and background signal in representative APT datasets 8

Supplementary Fig. 9 | Reconstruction-parameter sensitivity of deuterium partitioning in the $(CoCrNi)_{94}Al_3Ti_3$ alloy 9

Supplementary Fig. 10 | Solution energy results with and without ZPE and entropy corrections 10

Supplementary Fig. 11 | Energy results of isotopic effects in CoCrNi, $L1_2$ $Ni_3(Al, Ti)$, and $D0_{22}$ Ni_3Nb 10

Supplementary Table 1 | Positron Annihilation results 11

Supplementary Table 2 | Bulk composition of D-charged CoCrFeMnNi APT dataset 11

Supplementary Table 3 | Bulk compositions of the D-charged $(CoCrNi)_{94}Al_3Ti_3$ APT dataset. 11

Supplementary Table 4 | Bulk compositions of D-charged Inconel 718 superalloy APT dataset 12

Supplementary Table 5 | Summary of the 2 Da signal and local low-mass noise analysis for the 8 successful cryo-APT datasets out of 68 trials. The table reports the corresponding dataset, total ion count, 2 Da ion count, measured 2 Da ionic fraction and statistical uncertainty, together with the ion count, ionic fraction and statistical uncertainty measured from the peak-free noise region around 2.5 Da 12

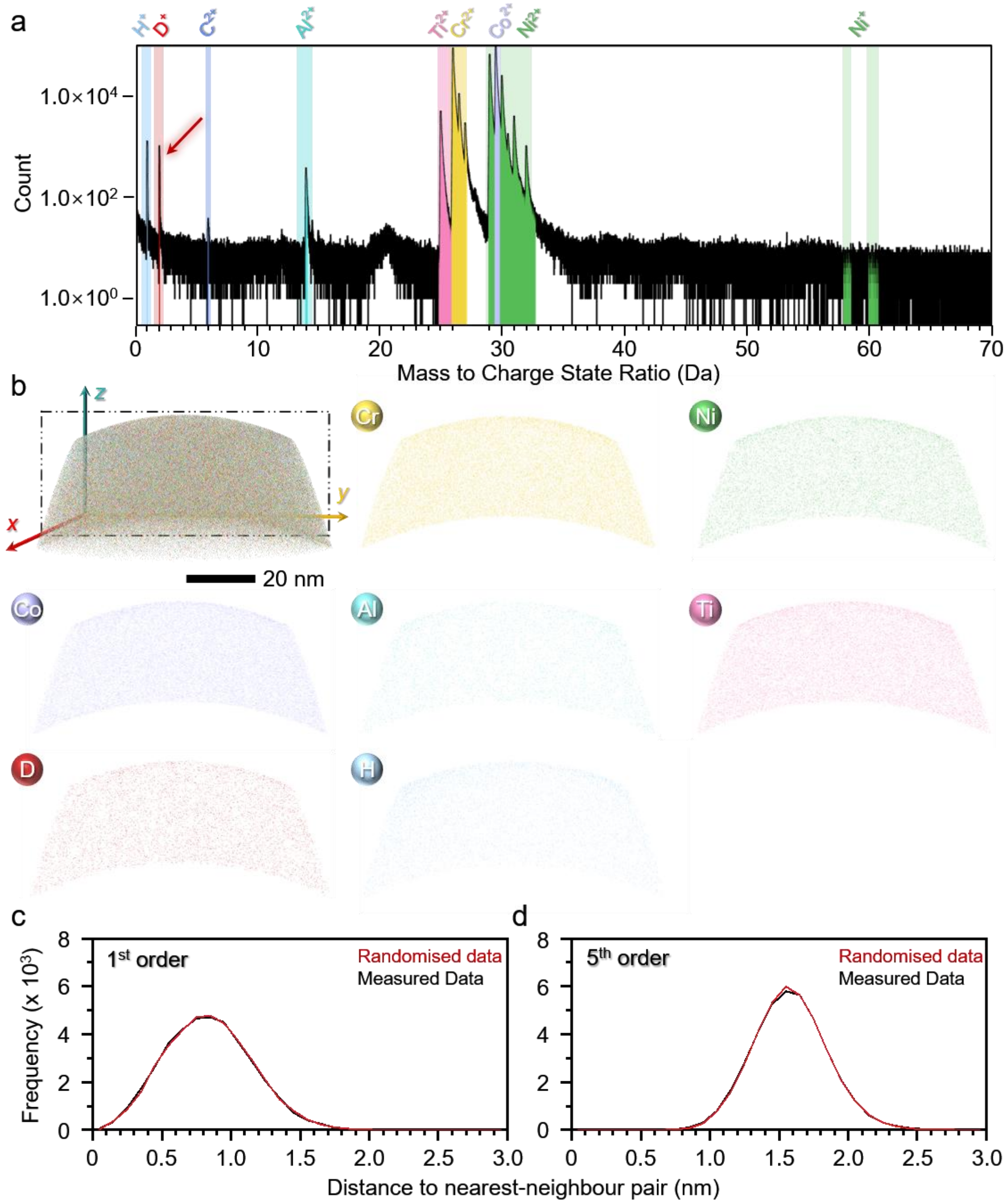

Supplementary Fig. 1 | Voltage-pulsing APT data of a D-charged single-phase $(CoCrNi)_{94}Al_3Ti_3$ specimen: (a) Mass spectrum with the presence of D peak (red). (b) Three-dimensional reconstruction of all elements and the 20-nm slice views of the principal elements (Co, Cr, Ni, Al, Ti), background hydrogen (blue), and deuterium (red). (c) Distribution of D–D pair distances for the 1st nearest D neighbor, compared with a randomized dataset, both peaking at approximately 0.8 nm, indicating a random D distribution. (d) Same analysis for the 5th nearest D neighbor, with a most probable distance of approximately 1.5 nm, consistent with random, solid-solution-like interstitial deuterium.

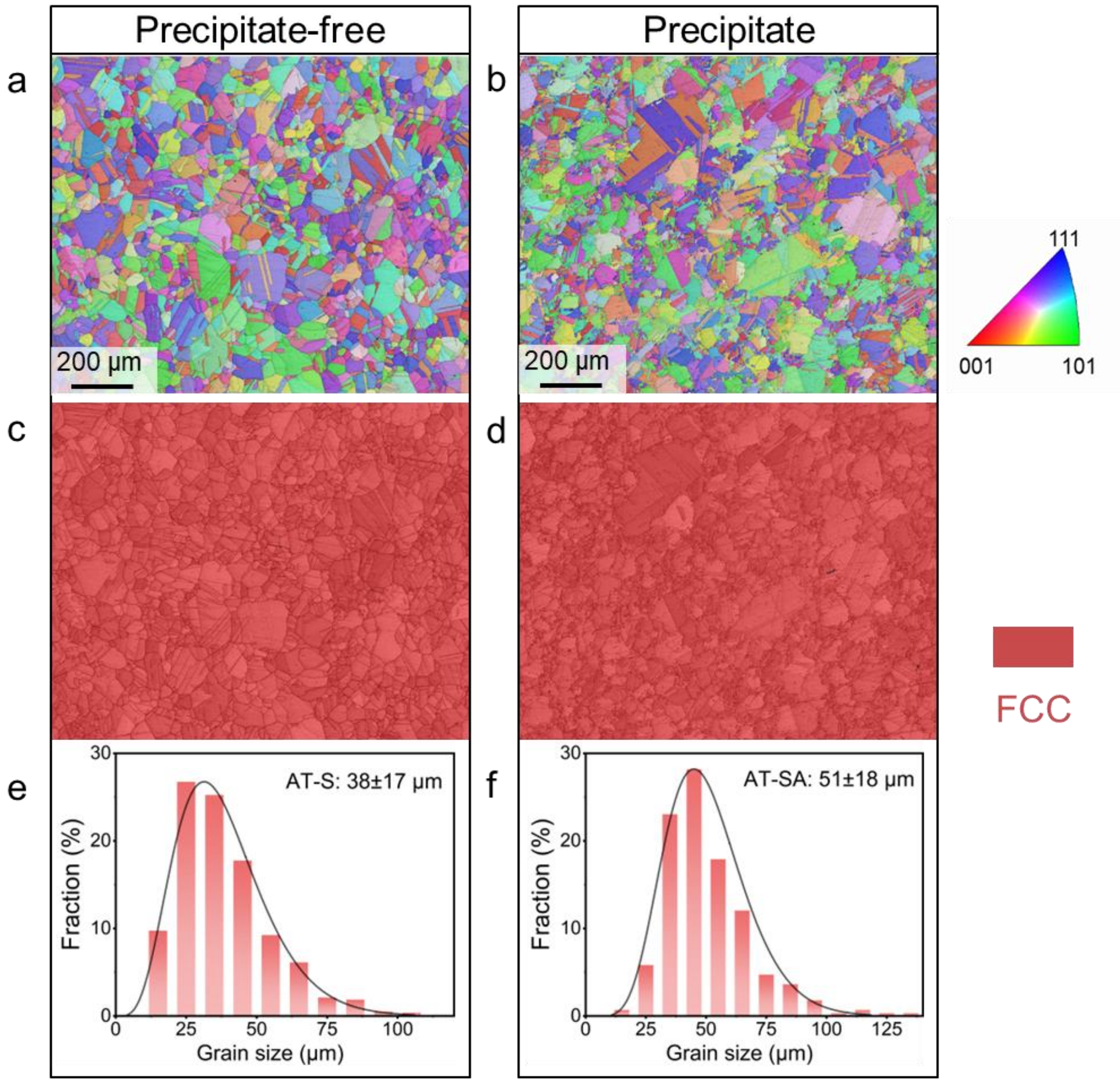

Supplementary Fig. 2 | Electron backscatter diffraction analyses of the FCC $(CoCrNi)_{94}Al_3Ti_3$ specimens before and after ageing at 800 °C for 5 hours: (a, b) Inverse pole figure maps before and after ageing, respectively. (c, d) Phase maps overlaid on band-contrast images before and after ageing, respectively. (e, f) Grain size statistics before and after ageing, respectively.

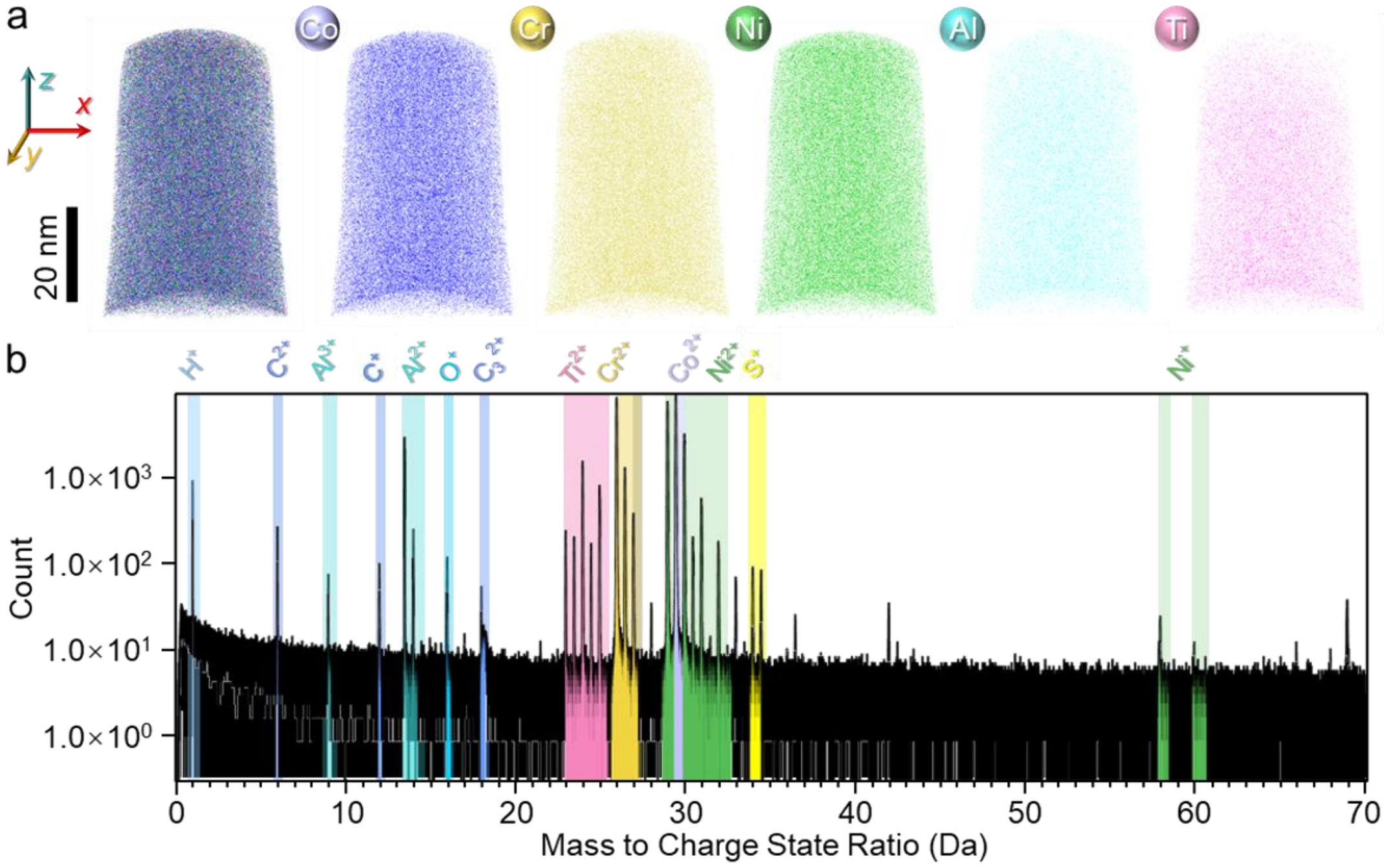

Supplementary Fig. 3 | Voltage-pulsing atom probe tomography data of deuterium-free single-phase $(CoCrNi)_{94}Al_3Ti_3$ alloy corresponding to Fig. 2. (a) Three-dimensional reconstruction of all elements and those of the principal elements (Co, Cr, Ni, Al and Ti). (b) Mass spectrum consistent with Fig. 2i.

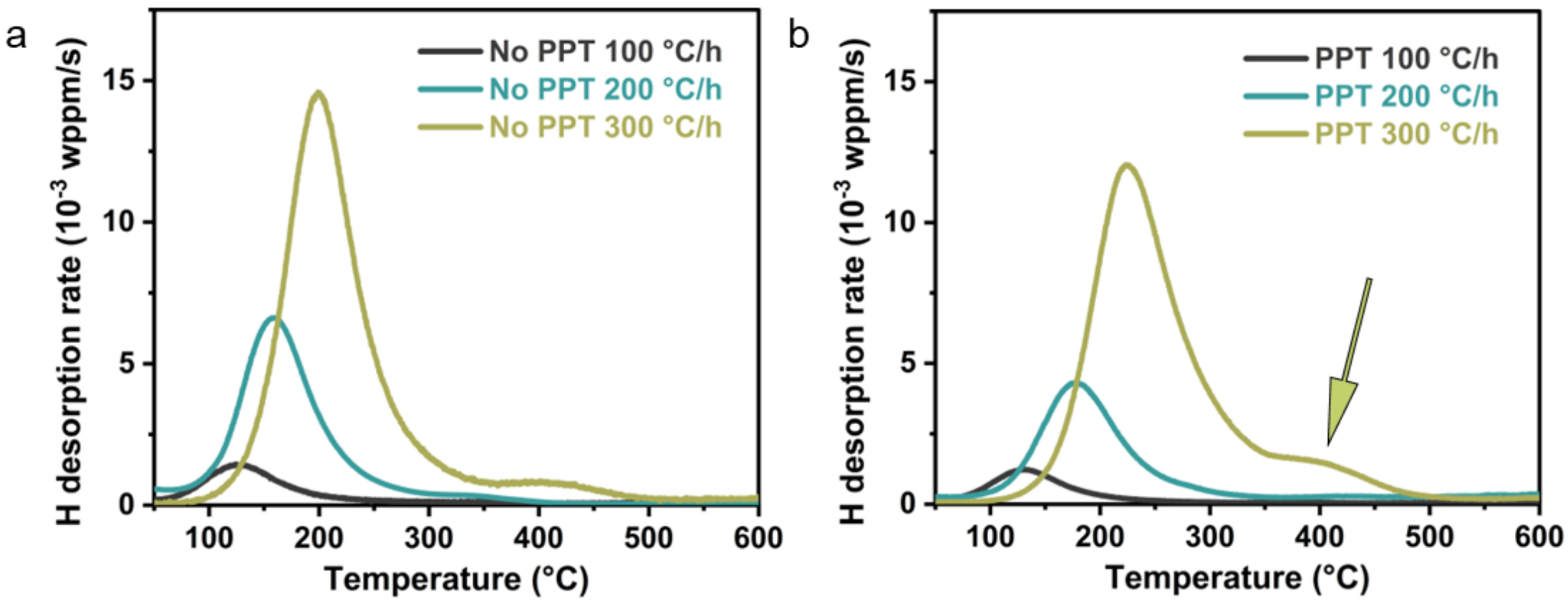

Supplementary Fig. 4 | Thermal desorption spectroscopy results from $(CoCrNi)_{94}Al_3Ti_3$ specimens with various heating rate. (a) Hydrogen desorption profile of precipitate-free $(CoCrNi)_{94}Al_3Ti_3$ specimen. (b) Hydrogen desorption profile of precipitated $(CoCrNi)_{94}Al_3Ti_3$ specimen, showing an increase of hydrogen desorption at high temperature (arrow).

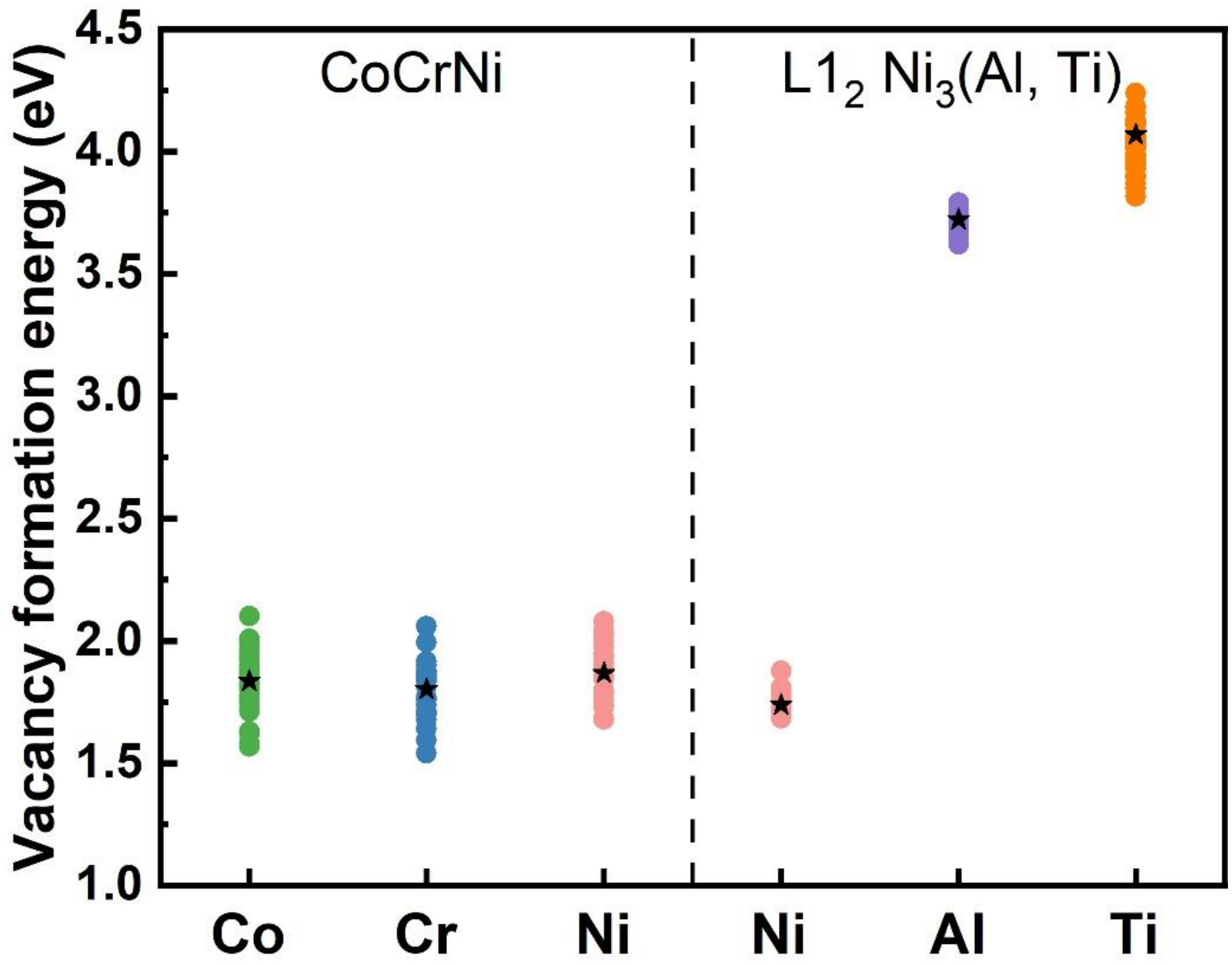

Supplementary Fig. 5 | Vacancy formation energies in CoCrNi and L1$_2$ Ni$_3$(Al, Ti).

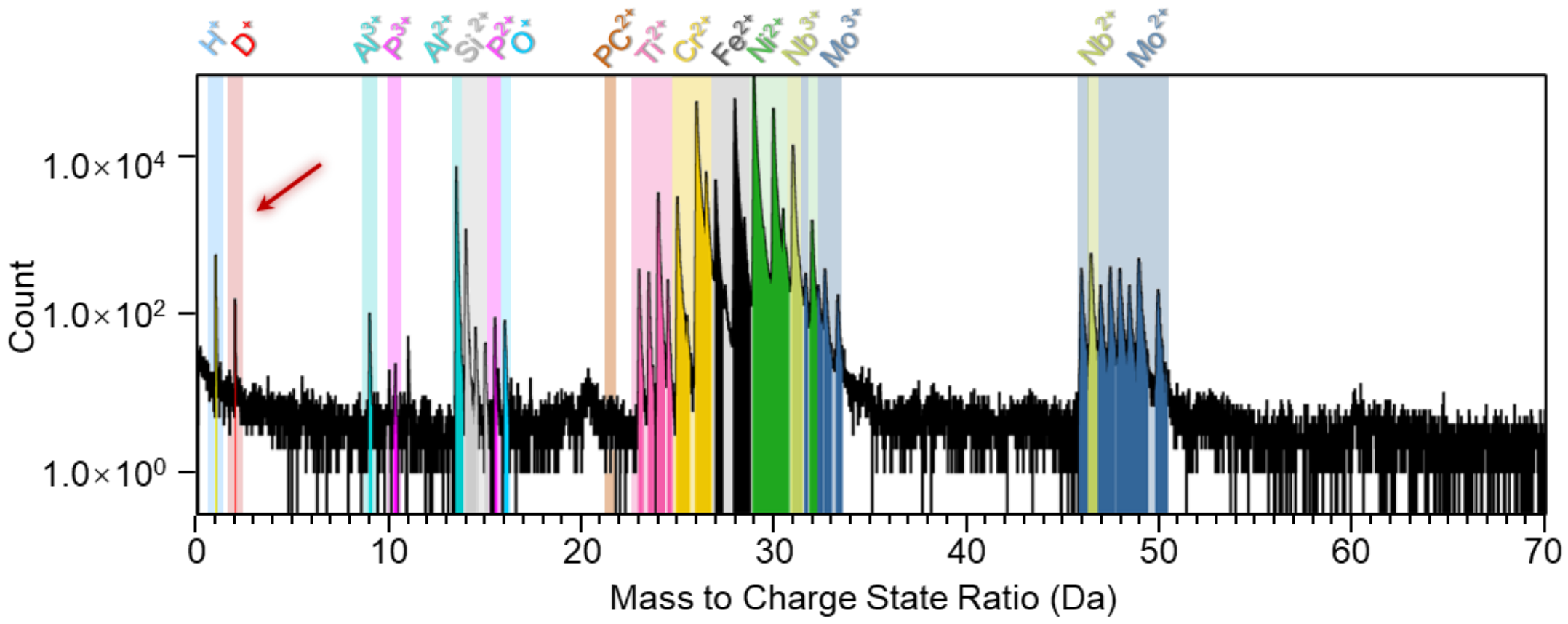

Supplementary Fig. 6 | Mass spectrum of the D-charged, dual-precipitate Inconel 718 nickel-based superalloy. This is from the reproduced voltage-pulsing APT data corresponding to Fig. 4, showing the presence of the D peak (red).

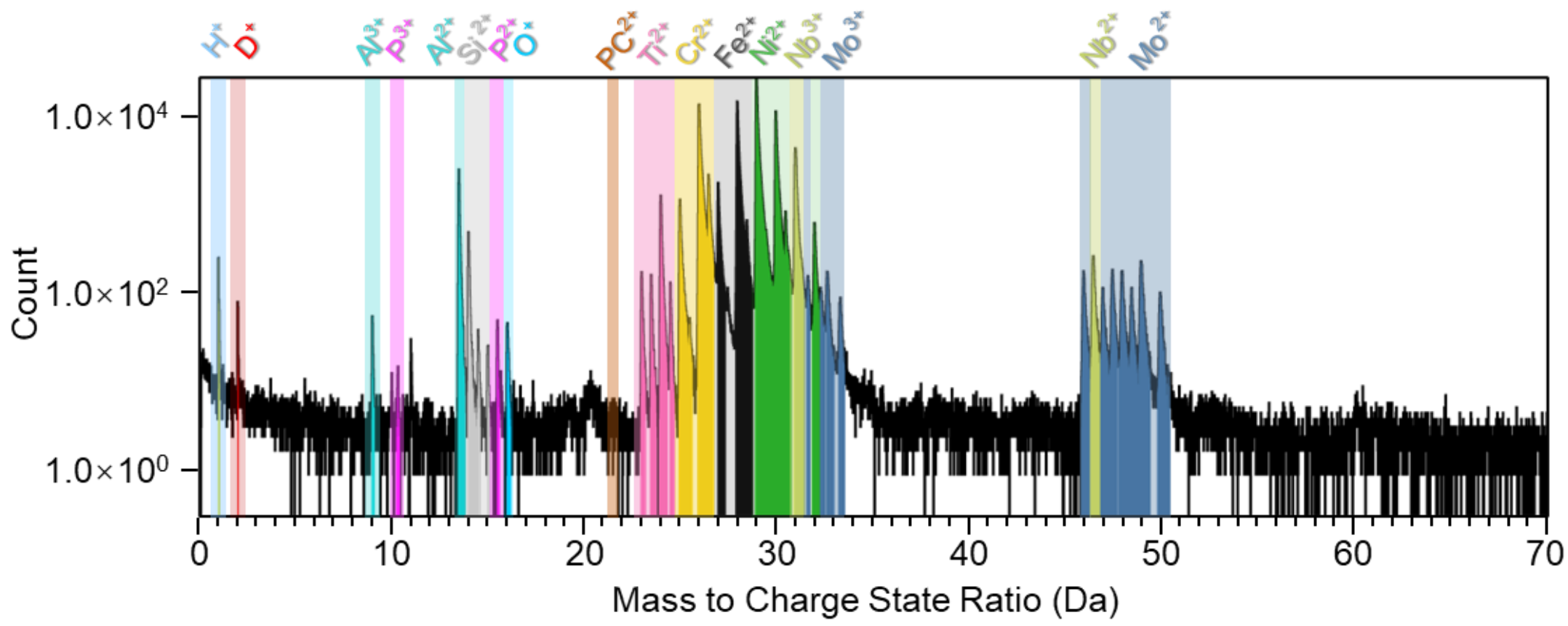

Supplementary Fig. 7 | Mass spectrum of the D-charged, dual-precipitate Inconel 718 nickel-based superalloy. This is from the reproduced voltage-pulsing APT data corresponding to Extended Data Fig. 9, showing the presence of the D peak (red).

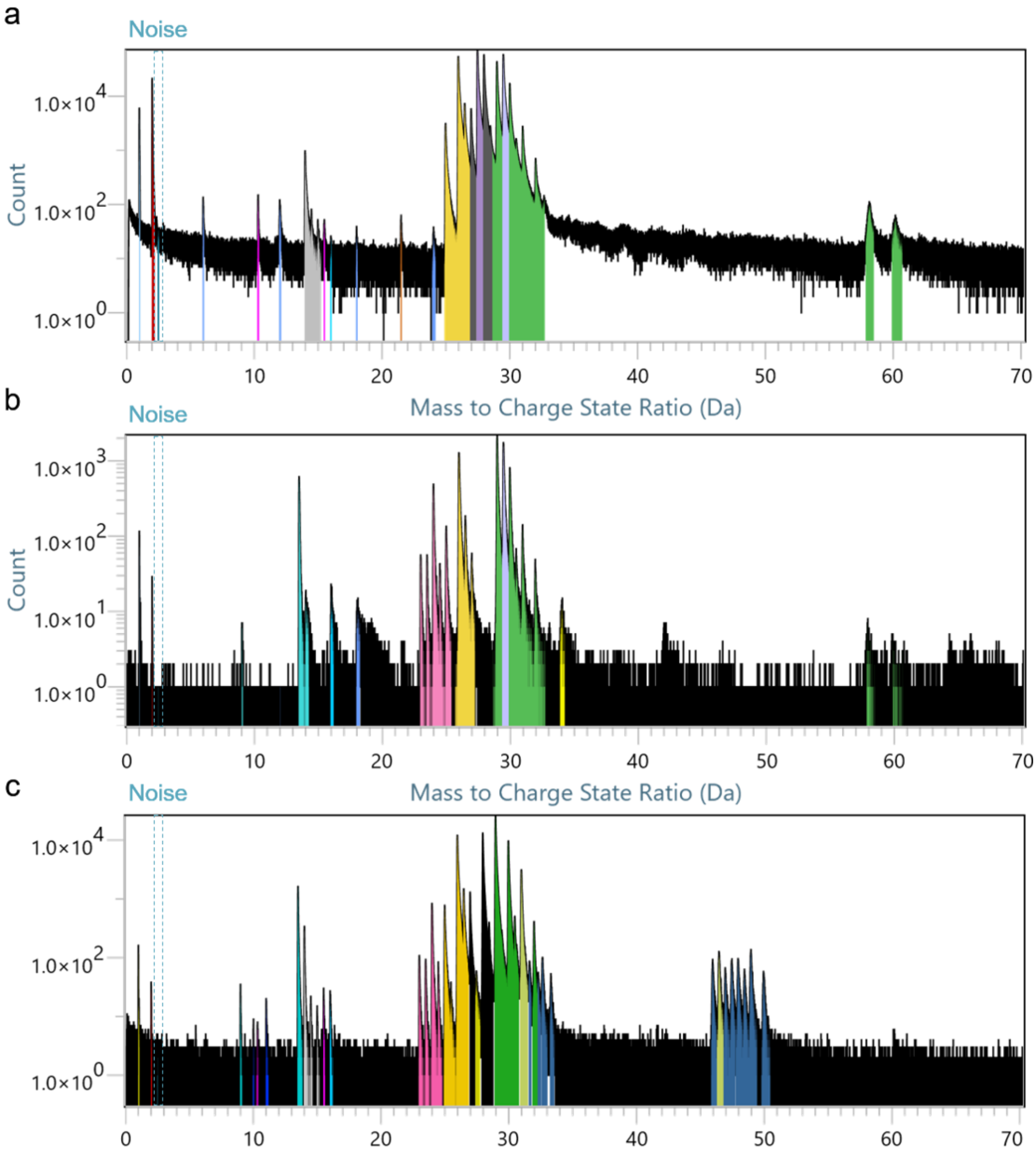

Supplementary Fig. 8 | Assessment of peak ranging and background signal in representative APT datasets. Mass spectra of representative datasets from (a) CoCrFeMnNi, (b) $(CoCrNi)_{94}Al_3Ti_3$ and (c) Inconel 718 in main text. The dashed region marks a peak-free mass-to-charge-state-ratio window around 2.5 Da, which was used to estimate the background/noise contribution.

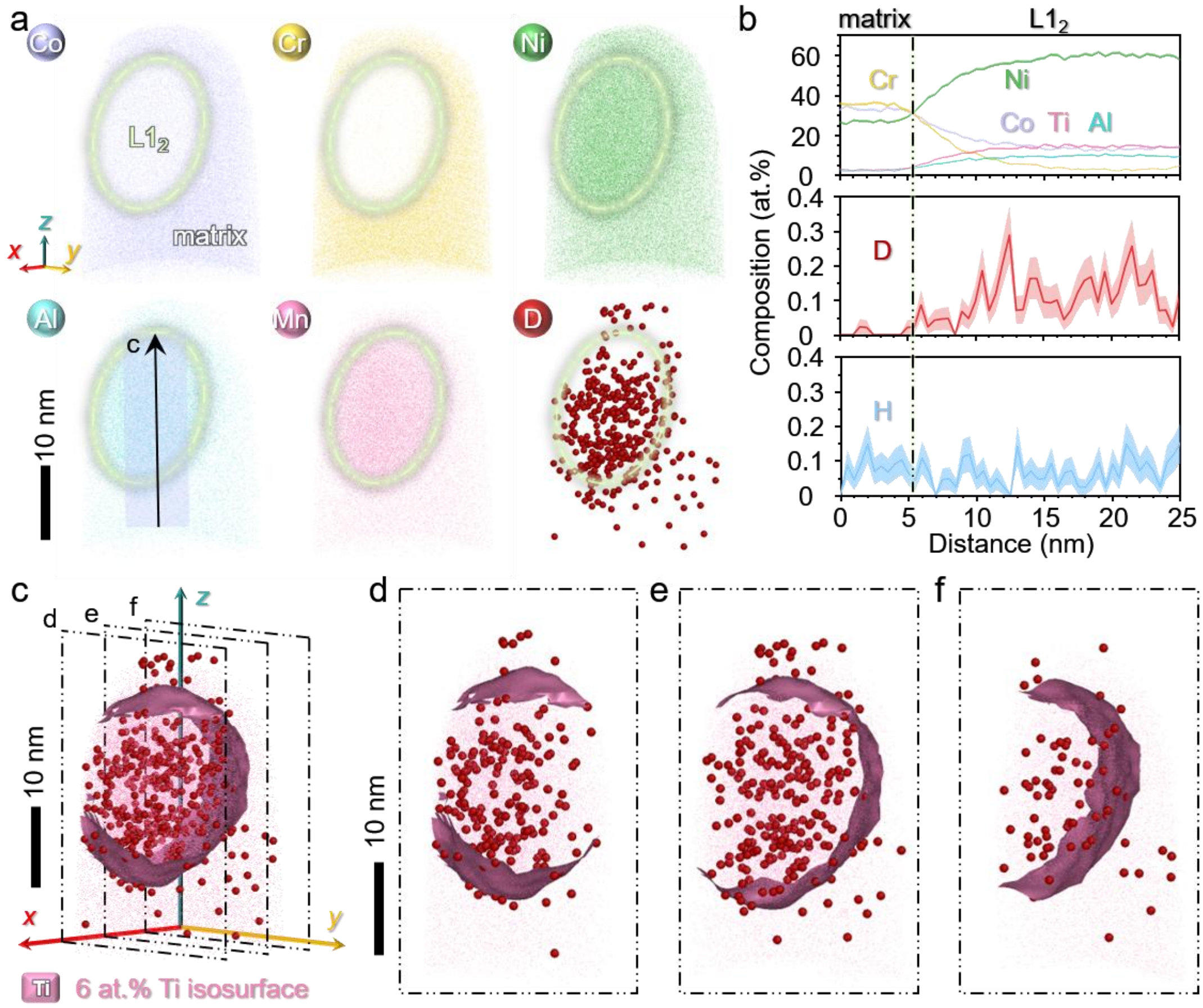

Supplementary Fig. 9 | Reconstruction-parameter sensitivity of deuterium partitioning in the $(CoCrNi)_{94}Al_3Ti_3$ alloy. The same APT dataset was reconstructed using a different image compression factor (ICF = 1.3). Although the reconstructed specimen geometry changes slightly, deuterium remains preferentially enriched within the $L1_2$ precipitate, with a similar concentration to that obtained from the reconstruction used in Fig. 3 of main text (ICF = 1.5). This indicates that reasonable variations in reconstruction parameters do not alter the conclusion that deuterium is preferentially enriched within the $L1_2$ precipitate.

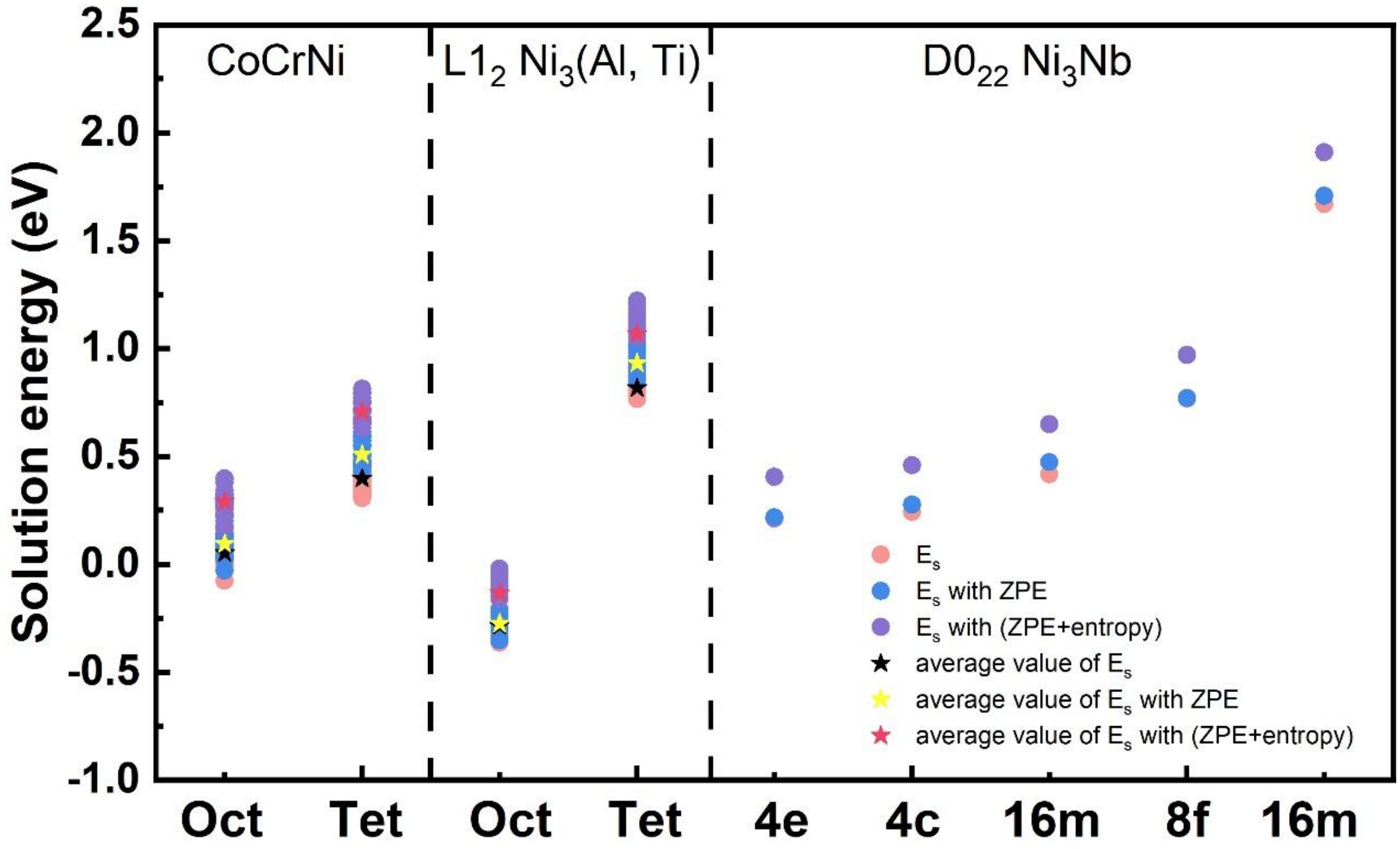

Supplementary Fig. 10 | Solution energy results with and without ZPE and entropy corrections.

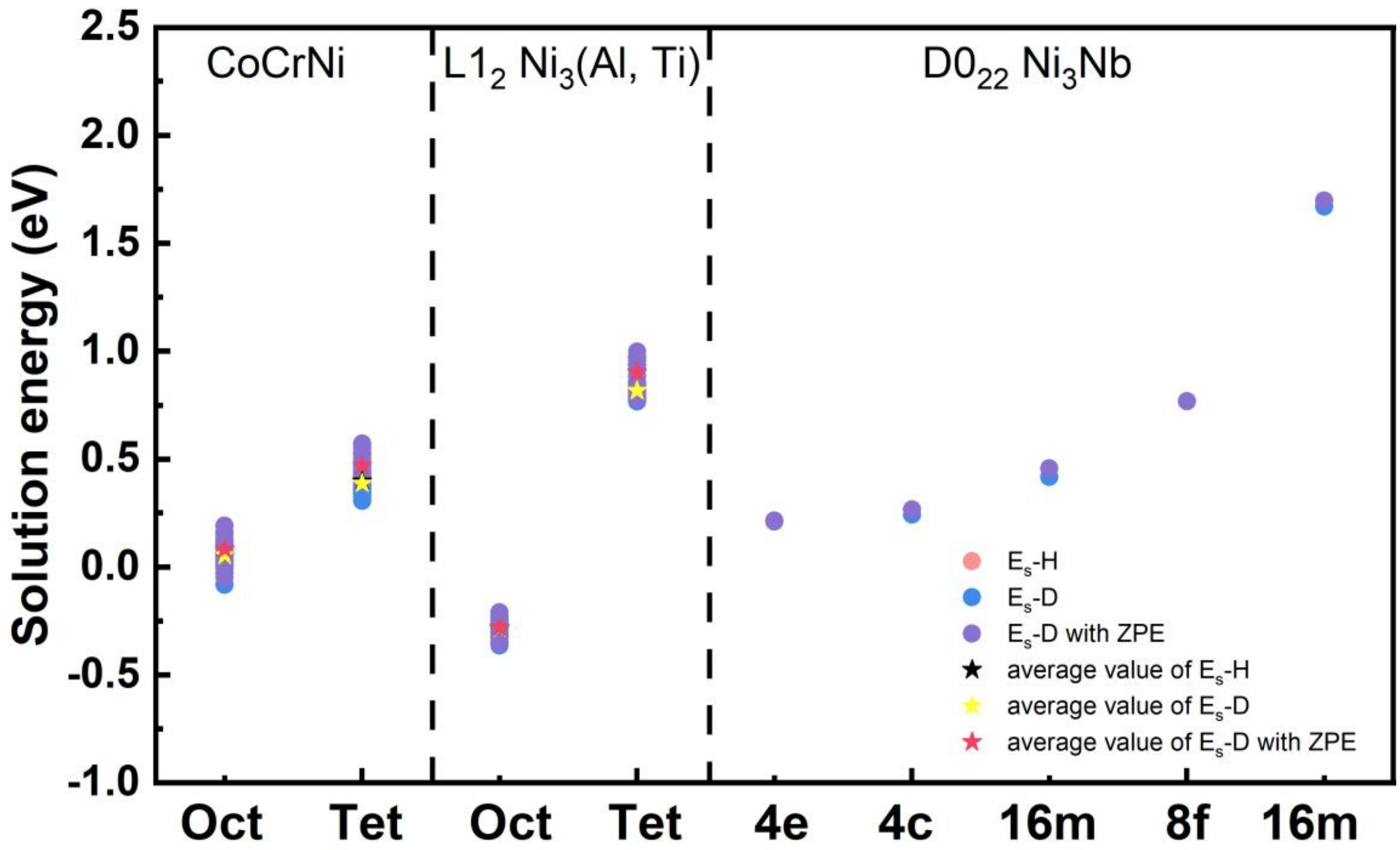

Supplementary Fig. 11 | Energy results of isotopic effects in CoCrNi, L1$_2$ Ni$_3$(Al, Ti), and D0$_{22}$ Ni$_3$Nb.

Supplementary Table 1 | Positron Annihilation results.

Specimen	Description	Lifetime (ns)	Relative intensity
Ref. Ni specimen 1	Pure Ni annealed at 1000 ℃ for 1 hour	0.269 ± 0.007	9.01% ± 0.49%
Ref. Ni specimen 2	Pure Ni annealed at 1000 ℃, compressed for 50%, and then annealed again at 1000 ℃, 1 hour	0.258 ± 0.021	6.20% ± 1.30%
Homogenized MPEA without vacancies	$(CoCrNi)_{94}Al_3Ti_3$ solutionized at 1000 ℃, 1 hour	Not detectable	Not detectable
Homogenized MPEA with vacancies	$(CoCrNi)_{94}Al_3Ti_3$ solutionized at 1000 ℃, 1 hour, and tensile strained for 2%	Not detectable	Not detectable
Precipitated MPEA without vacancies	$(CoCrNi)_{94}Al_3Ti_3$ solutionized at 1000 ℃ for 1 hour, aged at 800 ℃, 5 hours	Not detectable	Not detectable
Precipitated MPEA with vacancies	$(CoCrNi)_{94}Al_3Ti_3$ solutionized at 1000 ℃ for 1 hour, aged at 800 ℃, 5 hours, and strained for 2%	Not detectable	Not detectable

Supplementary Table 2 | Bulk composition of D-charged CoCrFeMnNi APT dataset.

Ion Type	Ion Count	Ionic Fraction (%)	Ionic Fraction (sigma) (%)
**Ni**	7981596	22.54	0.0088
**Mn**	6997942	19.77	0.0082
**Fe**	6914582	19.53	0.0081
**Cr**	6534182	18.46	0.0079
**Co**	6228282	17.59	0.0076
**D**	510975	1.44	0.0020
**Si**	99307	0.28	0.00089
**H**	97449	0.28	0.00088
**C**	21901	0.062	0.00042
**P**	10030	0.028	0.00028
**Noise**	3437	0.0097	0.00017
**PC2**	3060	0.0086	0.00016
**O**	1567	0.0044	0.00011

Supplementary Table 3 | Bulk compositions of the D-charged $(CoCrNi)_{94}Al_3Ti_3$ APT dataset.

Ion Type	Ion Count	Ionic Fraction (%)	Ionic Fraction (sigma) (%)
**Ni**	302294	41.84	0.091
**Co**	174150	24.10	0.064
**Cr**	148163	20.50	0.059
**Ti**	52535	7.27	0.033
**Al**	38663	5.35	0.028
**O**	1839	0.25	0.0059
**S**	1719	0.24	0.0057
**C**	1658	0.23	0.0056
**H**	1132	0.16	0.0047
**D**	362	0.050	0.0026
**Noise**	10	0.0014	0.00044

Supplementary Table 4 | Bulk compositions of D-charged Inconel 718 superalloy APT dataset.

Ion Type	Ion Count	Ionic Fraction (%)	Ionic Fraction (sigma) (%)
**Ni**	1152070	50.10	0.057
**Cr**	464239	20.19	0.032
**Fe**	454568	19.77	0.032
**Nb**	121210	5.27	0.016
**Mo**	43455	1.89	0.0092
**Al**	29287	1.27	0.0075
**Ti**	25513	1.11	0.0070
**Si**	5950	0.26	0.0034
**H**	1592	0.069	0.0017
**O**	538	0.023	0.0010
**P**	530	0.023	0.0010
**D**	365	0.016	0.00083
**Noise**	43	0.0019	0.00029

Supplementary Table 5 | Summary of the 2 Da signal and local low-mass noise analysis for the 8 successful cryo-APT datasets out of 68 trials. The table reports the corresponding dataset, total ion count, 2 Da ion count, measured 2 Da ionic fraction and statistical uncertainty, together with the ion count, ionic fraction and statistical uncertainty measured from the peak-free noise region around 2.5 Da.

No.	Corresponding Dataset	Original Dataset	Total Ion Count	2 Da Ion Count	2 Da Ionic Fraction (%)	2 Da Ionic Fraction (sigma) (%)	Noise Ion Count	Noise Ionic Fraction (%)	Noise Ionic Fraction (sigma) (%)
**1**	Fig. 1	R18_60357	35404310	510975	1.44	0.0020	3437	0.0097	0.00017
**2**	Fig. 3	R18_63173	722525	362	0.050	0.0026	10	0.0014	0.00044
**3**	Fig. 4	R18_64108	2299358	365	0.016	0.00083	43	0.0019	0.00029
**4**	Extended Data Fig. 1	R18_60355	13208238	190823	1.45	0.0033	786	0.0060	0.00021
**5**	Extended Data Fig. 3	R18_64543	9925962	288199	2.90	0.0055	266	0.0027	0.00016
**6**	Supplementary Fig. 1	R18_64538	32513297	38913	0.12	0.00061	617	0.0019	0.000076
**7**	Extended Data Fig. 4	R18_63174	4991869	390	0.0078	0.00040	85	0.0017	0.00019
**8**	Extended Data Fig. 9	R18_64128	6107879	610	0.010	0.00040	78	0.0013	0.00015
