# Supplementary material for: Towards observing interstitial hydrogen in solution in metal lattices
Source: Nature. 2026 Nov 4;659(8140):357–64. doi: 10.1038/s41586-026-11238-4 (PMC13754584; doi:10.1038/s41586-026-11238-4)
Supplement: Supplementary file 2 [file 41586_2026_11238_MOESM2_ESM.pdf]

nature portfolio

Peer Review File

Toward observing interstitial hydrogen in solution in metal lattices

Corresponding Author: Professor Eason Yi-Sheng Chen

Any redactions in this file are there to maintain patient confidentiality, the confidentiality of unpublished data, or to remove third-party material.

This file contains all reviewer reports in order by version, followed by all author rebuttals in order by version.

Version 0:

Reviewer comments:

Referee #1

(Remarks to the Author)
The study describes Atomic Probe Tomography (ATP) dual-phase and multi-precipitate alloys such as FCC CoCrNiFeMn, (CoCrNi)94Al3Ti3 with FCC L12 Ni3(Al,Ti) intermetallic precipitates with coherent interfaces with the matrix to demonstrate chemistry-dependent deuterium partitioning between FCC matrix and precipitates. Remarkably, the D atoms seem to be found predominantly inside the L12 precipitates. The APT results seem to align with DFT-predicted solution energies, which indicate which can enable direct detection of hydrogen on such materials. The measurements and calculations are well done, and the study provides useful insights into the mechanism of hydrogen effects in complex alloy systems. However, some areas of the manuscript require some additional attention.
1. The main criticism is that broad conclusions are drawn from specific CoCrNiFeMn FCC high-entropy alloys with coherent precipitates, but neglect broader validation, for example in other broad classes of high-entropy alloys. This potentially overstates APT's readiness for routine interstitial H analysis in diverse materials, as implied in the manuscript. This reviewer recommends expanding this study to at least another alloy class to assess generality of this approach and findings.
2. Figure 3 shows that the $Ni_3(Al,Ti)$ $L1_2$ precipitates contain a higher concentration than the surrounding CoCrNi FCC matrix, is it possible that at least some hydrogen (or deuterium) is located in trapping sites?
3. The Conclusion section if too general and it really doesn't really capture the depth and the mechanistic aspects of these results. What are the broader implications of this work?
4. The nearest-neighbor analyses and vacancy energy calculations seem to indicate against random vacancy trapping, but what are the reasons behind this?
5. The work should also address the FIB preparation effects, which literature highlights as persistent challenges in APT hydrogen detection.

Referee #2

(Remarks to the Author)
This manuscript addresses an important and challenging question: whether atom probe tomography can reliably distinguish interstitial hydrogen in solid solution from hydrogen trapped at defects or introduced by artefacts. The work is ambitious and technically strong, with clear strengths including the use of deuterium, cryo-transfer, and a combined APT–TDS-DFT approach applied to three FCC systems of increasing complexity. The results convincingly show a phase-dependent deuterium partitioning, consistent with differences in chemical affinity between phases. However, the manuscript over-interprets these findings. While the observed partitioning is robust, it does not unambiguously demonstrate that the signal corresponds exclusively to interstitial hydrogen, as contributions from vacancies, V-Hn complexes, defects, elastic fields, local chemical heterogeneities, or kinetic effects cannot be fully excluded. The main limitation is therefore not the data quality, but the level of certainty claimed.
The experimental approach relies on three materials (CoCrNiFeMn, (CoCrNi)94Al3Ti3 with L12 precipitates, and Inconel 718 with L12 and D022), showing increasing chemical contrast. Deuterium is introduced by electrochemical charging followed by cryo-transfer, and APT reveals random distribution in the single-phase alloy and preferential partitioning in L12 phases in multiphase systems, in qualitative agreement with DFT calculations. The authors attempt to exclude vacancy

trapping through thermodynamic estimates and PAS measurements, but these arguments remain insufficient, especially under non-equilibrium charging conditions.

Overall, the work is promising and potentially suitable for a journal such as Nature, but requires major revision. The authors should moderate their claims and, if possible, include additional controls addressing vacancy-related trapping, kinetic effects, H/D isotope differences, and APT quantification uncertainties.

Major Comments:
1) The use of three materials is well motivated and provides a clear progression from a simple system to a complex industrial alloy, with Inconel 718 offering a particularly relevant internal comparison. However, the experimental design is not orthogonal, as multiple parameters are changed simultaneously (composition, chemical disorder, precipitates, coherency, elastic fields, thermal history, and even experimental timing). As a result, the observed deuterium distribution cannot be attributed solely to chemical effects. This limitation becomes critical when claiming intrinsic chemical dependence, since microstructural and kinetic factors are also known to strongly influence hydrogen behavior in complex FCC alloys.

2) The argument excluding vacancy trapping is not fully convincing. It relies on equilibrium thermodynamics, while the experiments are performed under non-equilibrium electrochemical charging conditions known to generate superabundant vacancies and V-H_n complexes. Therefore, converting deuterium content into an equilibrium vacancy formation energy is not sufficient to rule out vacancy-related contributions. In addition, the use of the precipitation temperature is not appropriate, as relevant defects are likely formed during room-temperature charging and subsequent handling. Finally, the PAS measurements are performed on non-charged states and do not probe the actual charged condition; moreover, PAS sensitivity depends on defect chemistry, so hydrogen-decorated vacancies cannot be excluded

3) The protocol introduces a significant kinetic bias. The delay before quenching allows diffusion and preferential desorption, meaning the measured distribution is not a direct snapshot after charging. As shown in recent cryo-APT studies, such short thermal pauses can affect hydrogen redistribution, so the results likely reflect a combination of thermodynamic and kinetic effects.

4) The manuscript largely neglects the role of SFE, although it is central in FCC alloys. Hydrogen is known to modify SFE and interact strongly with dislocations and planar defects. In HEA/MEA systems, local chemical order produces a wide distribution of fault energies and defect structures. Therefore, part of the observed deuterium distribution may be associated with defect structures rather than purely interstitial solution.

5)The enrichment of deuterium in L12 precipitates is interpreted as a chemical effect. However, coherent precipitates generate elastic fields due to lattice misfit. Hydrogen solubility is known to depend on hydrostatic stress. Therefore, the observed partitioning is likely a combined chemical-elastic effect, not purely chemical.

6) The DFT approach captures average trends but does not fully represent the broad distribution of interstitial site energies expected in chemically complex alloys. In addition, the calculations are limited to ideal interstitial configurations and do not account for competing states such as vacancy–hydrogen complexes or defect-associated trapping. As a result, the interpretation in terms of a single solution energy per phase is likely oversimplified, and the DFT results should be viewed as indicative trends rather than a complete description of hydrogen behavior

7) DFT calculations are performed at 0K, without ZPE, entropy, or defect contributions. They describe ideal interstitial solutions, not the full competition between possible hydrogen states.

8) The use of deuterium is appropriate, but isotopic effects (ZPE, diffusivity, binding energies) are not considered in calculations. This limits strict quantitative comparison

9) APT quantification needs stronger support. Although best practices are followed, key sources of uncertainty (mass overlaps, background signal, and reconstruction sensitivity) are not fully documented, which limits the strength of the conclusions.

Minor Comments;

1) Language should be moderated (avoid “unambiguous”, “direct evidence”).
2) TDS comparison is indirect due to different protocols.
3) More details on statistics and reproducibility are needed.

To conclude, the manuscript provides a significant contribution by demonstrating phase-dependent deuterium partitioning in FCC alloys. However, it does not yet prove unambiguously that the signal corresponds exclusively to interstitial hydrogen. The issue is not the absence of results, but the excess of certainty in their interpretation.

Here is a list of references that may help the authors address these points:

1.Fukai, Y. The Metal–Hydrogen System: Basic Bulk Properties, Springer Series in Materials Science, Springer, Berlin/Heidelberg, 1993, re edition 2005.
2.Fukai, Y. “Formation of superabundant vacancies in M–H alloys and some of its consequences: a review.” Journal of

Alloys and Compounds 356-357 (2003) 263–269.
3.Fukai, Y. and Okuma, N. “Evidence of copious vacancy formation in Ni and Pd under a high hydrogen pressure.” Japanese Journal of Applied Physics 32 (1993), L1256 - L1258
4.Kirchheim, R. “Reducing grain boundary, dislocation line and vacancy formation energies by solute segregation. I. Theoretical background.” Acta Mater 55 (2007), 5129–5138.
5.Nagumo, M. “Hydrogen related failure of steels - a new aspect.” Mater Sci and Technology 20 (2004), 940-950.
6. Jena, P. and Sinha, A. K. “Positron annihilation in metal-vacancy–hydrogen complexes.” Phys Rev B 24 (1981) 2884-2894.
7. An, X. et al. “Investigation of the interaction between hydrogen and vacancies by positron annihilation spectroscopy” Int J of Hydrogen Energy 46 (2021).
8. Gault, B. et al. “Towards Establishing Best Practice in the Analysis of Hydrogen and Deuterium by Atom Probe Tomography.” Microscopy and Microanalysis 30 (2024)
9. London, A. J. “Quantifying Uncertainty from Mass-Peak Overlaps in Atom Probe Microscopy.” Microscopy and Microanalysis 25 (2019) 378–388.
10. Mouton, I. et al. “Quantification Challenges for Atom Probe Tomography of Hydrogen and Deuterium in Zircaloy-4.” Microscopy and Microanalysis 25 (2019) 481-488.
11. Saksena, A. et al. “Optimizing site-specific specimen preparation for atom probe tomography by using hydrogen for visualizing radiation-induced damage.” Int. J of Hydrogen Energy 50 (2024), 165-174.
12.Wang, S. et al. “Systematic quantification of hydrogen in pipeline steel by atom probe tomography after ambient charging and transfer.” Materials Degradation 8(2024)
13. Vukkum, V. B. et al. “Insights from quasi-in situ cryogenic-transfer atom probe tomography for analyzing hydrogen diffusion in metallic alloys ” npj Materials Degradation 9 (2025).
14. Hermida, J. D. and Roviglione, A. “Stacking fault energy decrease in austenitic stainless steels induced by hydrogen pairs formation.” Scripta Mater 39 (1998) 1145-1149.
15. He, Y. et al. “Effect of hydrogen atom and hydrogen-filled vacancies on stacking fault energy in γ-Fe by first-principles calculations.” Int J of Hydrogen Energy 44 (2019), 17136-17145.
16. Xie, Z. et al. “Sluggish hydrogen diffusion and hydrogen decreasing stacking fault energy in a high-entropy alloy.” Materials Today Communications 26 (2021)
17. Ren, X. L. et al. “Hydrogen solution in high-entropy alloys.” Phys Chemistry Chemical Physics 23 (2021).
18. Wang, Y. Z. et al. “Disentangling diffusion heterogeneity in high-entropy alloys.” Acta mater 222 (2022).
19. Li, Q.-J. et al. “Strengthening in multi-principal element alloys with local-chemical-order roughened dislocation pathways.” Nature Communications 10 (2019) 3563.
20. Palumbo, O. et al. “Hydrogen and Deuterium Solubility, Diffusivity and Permeability from Sorption Measurements in the Ni33Ti39Nb28 Alloy.” Molecules 28 (2023) 1082.
21. Bourgeois, N. et al. “Vibration analysis of hydrogen, deuterium and tritium in metals: consequences on the isotope effect.” Journal of Physics: Condensed Matter 30 (2018)
22. Setyawan, W. et al. “Influence of hydrogen isotopes on vacancy formation and antisite defect diffusion in Pd and V.” Computational Materials Science 197 (2021).
23. Nazarov, R., Hickel, T. and Neugebauer, J. “First-principles study of the thermodynamics of hydrogen-vacancy interaction in fcc iron.” phys rev B 82 (2010) 224104.
24. Wang, Y. et al. “Hydrogen influence on diffusion in nickel from first-principles calculations.” Phys Rev B 91 (2015) 094106.
25. Song, J. and Curtin, W. A. “Atomic mechanism and prediction of hydrogen embrittlement in iron.” Nature Materials 12 (2013), 145-151.
26. Zhou, X. et al. “Mechanism and Prediction of Hydrogen Embrittlement in fcc Metals.” Phys Rev Letters 127 (2021), 175501.
27. Cheng, H. et al. “The influence of $L1_2$ ordered precipitates on hydrogen embrittlement behavior in CoCrNi-based medium entropy alloys.” Acta Mater 260 (2023), 119328.
28. Yoo, J. et al. “Effects of lattice misfit of γ/γ′ phases on hydrogen embrittlement behavior in Ni-based single crystal superalloy ” J of Materials Research and Technology (2024).
29. Shi, H. et al. “In situ investigation of the interaction between hydrogen and stacking faults in a bulk austenitic steel” Acta Mater(2024)

[redacted]

[redacted]

[redacted]

[redacted]

[redacted]

[redacted]

[redacted]

[redacted]

[redacted]

[redacted]

Version 1:

Reviewer comments:

Referee #1

(Remarks to the Author)
The authors have satisfactorily addressed the technical concerns and improved the presentation of the results. These revisions have significantly strengthened the work, and the manuscript is now suitable for publication.

Referee #2

(Remarks to the Author)
The authors have made a substantial effort to answer the reviewers' comments. I acknowledge the important additional work, including new APT datasets, quasi in situ TEM, positron annihilation measurements and additional DFT calculations. These new results reinforce the evidence for reproducible phase dependent deuterium partitioning. However, they do not demonstrate without ambiguity that the detected deuterium is located predominantly at defect-free interstitial sites. The main issue therefore remains the level of certainty used in the manuscript.

Major comments:

1) The title, abstract, introduction and conclusion still use strong expressions such as "observation of interstitial hydrogen," "direct experimental evidence" and "directly probe hydrogen in solid solution." More cautious wording is needed, as the results show deuterium partitioning consistent with interstitial hydrogen in solid solution, without directly identifying its atomic state.

2) The additional TEM and positron measurements make dominant defect trapping unlikely, but do not fully exclude isolated vacancies or V-Hn/V-Dn complexes. The equilibrium vacancy calculation at 800 °C is not directly applicable to defects generated during non-equilibrium room-temperature charging. Moreover, the small differences between the H-charged and pristine PAS curves should be quantified, together with the detection limit. The manuscript should therefore conclude that vacancy trapping is unlikely to be dominant, rather than excluded.

3. The 10s hold at room temperature promotes preferential deuterium desorption from the matrix. The measured distribution therefore reflects both thermodynamic and kinetic effects, rather than the state immediately after charging. The possible influence of elastic fields around the coherent precipitates should also be discussed.

4) The treatment of isotope effects needs clarification. Static DFT energies and classical NEB barriers are normally

independent of isotope mass. H/D differences require vibrational and ZPE corrections, including at the transition state. The authors should explain how the different H and D curves were obtained or restrict their conclusion to general energetic trends.

5) The APT uncertainty discussion should focus more directly on the low-mass region. A background noise measured around 55 Da does not quantify possible overlap between D+ and H_2^+ at 2 Da. The authors should provide the low-mass spectra, the number of independent tips, the D concentration for each replicate and a distinction between counting and systematic uncertainties.

Minor comments
1) Regarding page 20, I am not familiar with Ni94(Al,Ti)L12 precipitates; perhaps they are Ni3(Al,Ti)L12?
2) On page 49, I see "steel samples" those aren't steels!
3) The manuscript still contains several grammatical errors and should be carefully checked.

Version 2:

Reviewer comments:

Referee #2

(Remarks to the Author)
The authors have satisfactorily addressed all my comments. I have no further comments.

Open Access This Peer Review File is licensed under a Creative Commons Attribution 4.0 International License, which permits use, sharing, adaptation, distribution and reproduction in any medium or format, as long as you give appropriate credit to the original author(s) and the source, provide a link to the Creative Commons license, and indicate if changes were made.
In cases where reviewers are anonymous, credit should be given to 'Anonymous Referee' and the source.
The images or other third party material in this Peer Review File are included in the article's Creative Commons license, unless indicated otherwise in a credit line to the material. If material is not included in the article's Creative Commons license and your intended use is not permitted by statutory regulation or exceeds the permitted use, you will need to obtain permission directly from the copyright holder.
To view a copy of this license, visit https://creativecommons.org/licenses/by/4.0/

Response letter

Editor/Reviewers' comments in ***italic blue***, authors' responses in **black**, and revised content as in the revised manuscript in **highlighted black**

Editor:

While they find your work of interest, they have raised points that need to be addressed before we can make a decision on publication. These include, but are not limited to, the effect of FIB on what is detected in the samples as well as possible deuterium trapping in precipitates (Reviewer 1) but most importantly, the manuscript having to be revised so that it provides a more nuanced discussion of the uncertainties (Reviewer 2 but also to some degree Reviewer 1); Reviewer 1 also asks to extend the study to other systems, but as this is very onerous it is something we are willing to overlook as long as the text is softened in the revision (although if you do have additional data, I would encourage you to include it).

We have addressed all comments from the reviewers in the revised manuscript. Regarding the possible effect of FIB, we added discussion supported by literature as well as details about our cryogenic Xe-PFIB workflow for APT specimen preparation, clarifying that we used the best practice that minimized the introduction of defects during APT specimen preparation. Regarding uncertainties around possible presence of structural defects after hydrogen charging, which are not visible in APT reconstructions but can contribute to deuterium trapping, we performed a quasi in-situ TEM experiment that confirms the absence of charging-induced defects, such as stacking faults or dislocations, in the same area of observation before and after the same deuterium charging used for APT specimen.

In addition, we conducted a set of additional positron annihilation spectroscopy experiments and obtained the results that show no detectable vacancy-type signals after the same deuterium charging used for APT specimens. We compared the results with that of a reference specimen from the same material but was ion-irradiated and thus had detectable vacancy signals, suggesting the positron technique can detect vacancies effectively and confirm the low concentration of vacancy in our APT specimens. We have also extended our study to other MPEA systems and included new results from a single-phase CoNiV and a single-phase $(CoCrNi)_{94}Al_3Ti_3$, which show that high deuterium concentrations can be consistently detected in the MPEAs. Finally, we extended our DFT investigation with greater details to address Reviewer 2's concern about clarity. Although we have provided strong evidence to support our original statement about hydrogen solution, we still revised the description in the new version of manuscript with a more balanced and nuanced interpretation to minimize any possible confusion. Several conclusions were softened to avoid overstatement while preserving the main findings. We thank you for your patience while we were working on the key experiments for addressing reviewers' concerns. We thank you for considering our submission.

Referee #1 (Remarks to the Author):

The study describes Atomic Probe Tomography (ATP) dual-phase and multi-precipitate alloys such as FCC CoCrNiFeMn, $(CoCrNi)_{94}Al_3Ti_3$ with FCC L12 $Ni_3(Al,Ti)$ intermetallic precipitates with coherent interfaces with the matrix to demonstrate chemistry-dependent deuterium partitioning between FCC matrix and precipitates. Remarkably, the D atoms seem to be found predominantly inside the L12 precipitates. The APT results seem to align with DFT-predicted solution energies, which indicate which can enable direct detection of hydrogen on such materials. The measurements and calculations are well done, and the study provides useful insights into the mechanism of hydrogen effects in complex alloy systems. However, some areas of the manuscript require some additional attention.

We appreciate the reviewer's comments and have addressed all of them as detailed below.

1. The main criticism is that broad conclusions are drawn from specific CoCrNiFeMn FCC high-entropy alloys with coherent precipitates, but neglect broader validation, for example in other broad classes of high-entropy alloys. This potentially overstates APT's readiness for routine interstitial H analysis in diverse materials, as implied in the manuscript. This reviewer recommends expanding this study to at least another alloy class to assess generality of this approach and findings.

We expanded the study with two additional FCC high-entropy alloys, solution-treated CoNiV and $(CoCrNi)_{94}Al_3Ti_3$. Both alloys were prepared by melting followed by homogenization treatment to obtain single-phase FCC microstructures. As shown in the figures below, which have also been added as the Extended Data Figs. 3 and 4 in the revised manuscript, deuterium atoms were found to be randomly distributed within the matrix, supported by nearest-neighbor analyses, consistent with other results presented in the original manuscript for observing hydrogen in solution (but not trapped by defects). We also revised our description about the results, which now reads as below.

Page 10 Lines 3 to 6:

Random D distributions were also consistently observed in solution-treated CoNiV and $(CoCrNi)_{94}Al_3Ti_3$ alloys (Extended Data Figs. 3 and 4), supporting the generality of this observation among FCC MPEAs.

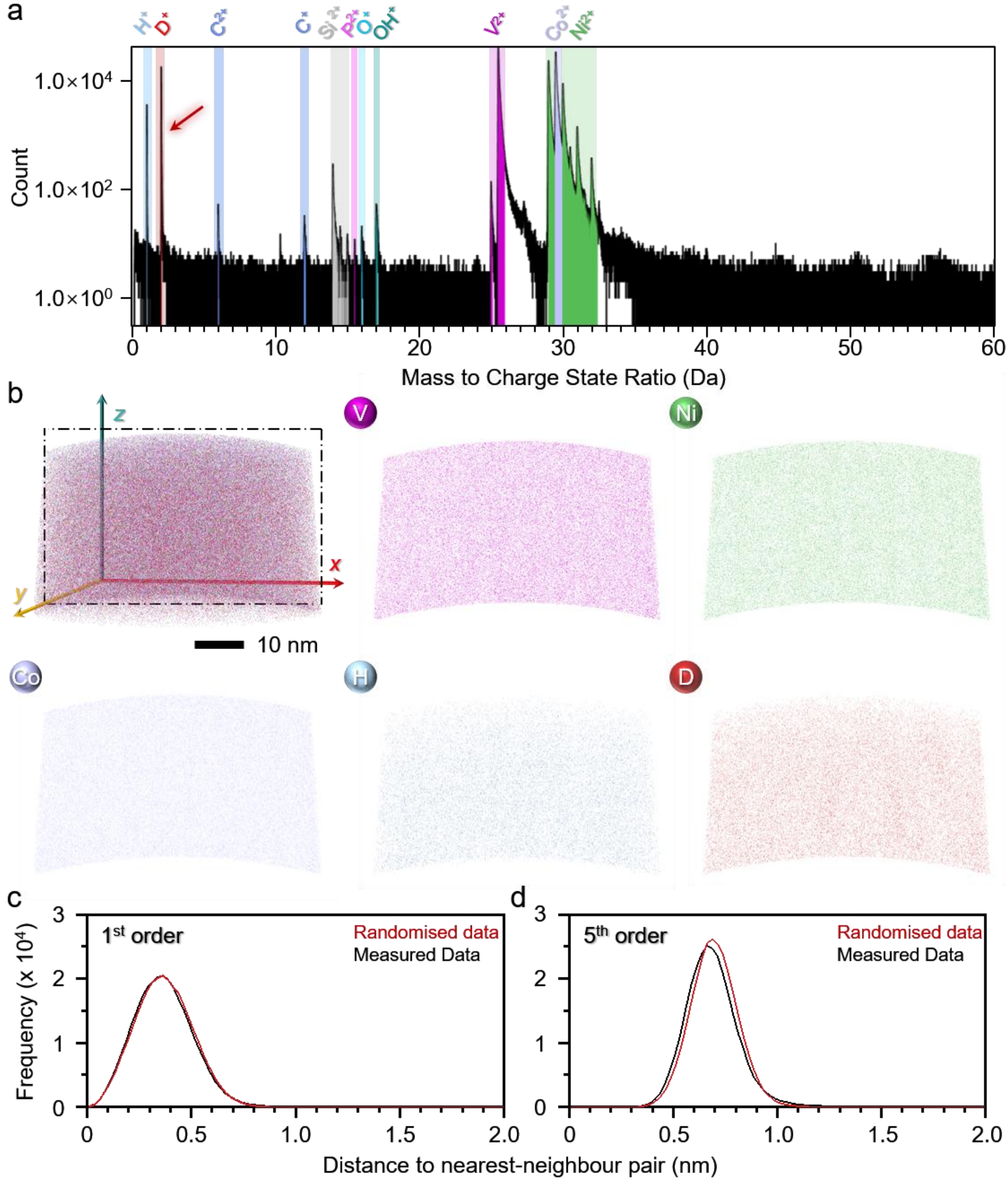

Extended Data Fig. 3 Voltage-pulsing APT data of a D-charged single-phase CoNiV specimen. a, Mass spectrum with the presence of D peak (red). **b,** Three-dimensional reconstruction of all elements and the 20-nm slice views of the principal elements (Co, Ni, V), background hydrogen (blue), and deuterium (red). **c,** Distribution of D–D pair distances for the 1st nearest D neighbor, compared with a randomized dataset, both peaking at approximately 0.4 nm, indicating a random D distribution. **d,** Same analysis for the 5th nearest D neighbor, with a most probable distance of approximately 0.7 nm, consistent with random, solid-solution-like interstitial deuterium.

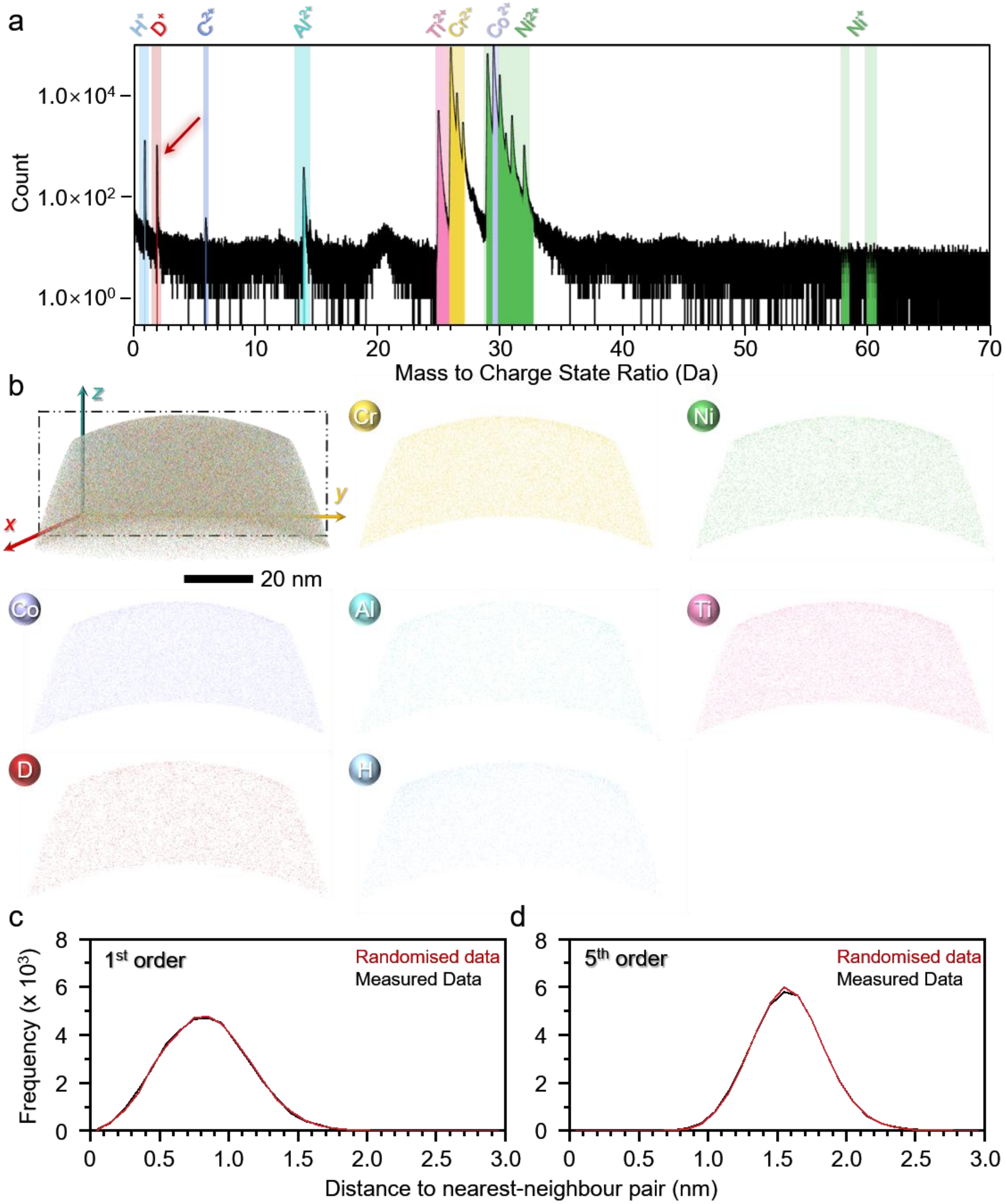

Extended Data Fig. 4 Voltage-pulsing APT data of a D-charged single-phase $(CoCrNi)_{94}Al_3Ti_3$ specimen. a, Mass spectrum with the presence of D peak (red). **b,** Three-dimensional reconstruction of all elements and the 20-nm slice views of the principal elements (Co, Cr, Ni, Al, Ti), background hydrogen (blue), and deuterium (red). **c,** Distribution of D–D pair distances for the 1st nearest D neighbor, compared with a randomized dataset, both peaking at approximately 0.8 nm, indicating a random D distribution. **d,** Same analysis for the 5th nearest D neighbor, with a most probable distance of approximately 1.5 nm, consistent with random, solid-solution-like interstitial deuterium.

2. Figure 3 shows that the $Ni_3(Al,Ti)$ $L1_2$ precipitates contain a higher concentration than the surrounding CoCrNi FCC matrix, is it possible that at least some hydrogen (or deuterium) is located in trapping sites?

We appreciate the reviewer's concern about the possible presence of defects that can contribute to trapping D atoms, contrasting to the D atoms in solution that are randomly located in given volume, and can lead to the observed concentration within the $L1_2$ precipitates. To address this, we extended our analyses in the revised manuscript, which now account for a) pre-existing defects in the material before charging and b) the defects that could be introduced during electrochemical D charging.

For a) pre-existing defects, our thermodynamic approximation, vacancy formation energy calculations, and positron annihilation spectroscopy measurements suggest that the concentration of vacancy-type defects in the uncharged material is too low to account for the measured high D concentration in the $L1_2$ precipitates. We also acknowledge that other defects, such as stacking faults or dislocations, could in principle contribute to hydrogen/deuterium trapping. However, the density of such defects required to explain the observed D enrichment would be unrealistically high, and no corresponding defect population was observed in our structural characterization. These results suggest that pre-existing defect trapping is unlikely to be the main cause of the observed D enrichment.

For b) the charging-induced defects, we conducted a set of quasi in-situ TEM experiments to examine whether the electrochemical D-charging process can introduce crystalline defects. The TEM observations were performed on the same $L1_2$ precipitates before and after D charging for 1.5 minutes, the parameter that is consistent with our APT D-charging process. As shown in Extended Data Fig. 10 attached below and in the revised manuscript, Figures a and b show the experimental setup and Figures c-h show the comparisons between the original and after-charging microstructures on the left and right, respectively. Figures c1 and c2 are low-magnification images from the same location before and after deuterium charging. Figures d1 and d2 are the HAADF-STEM images corresponding to Figures c1 and c2. Figures e1 and e2 are the HAADF-STEM images that include the two selected $L1_2$ precipitates, the corresponding diffraction patterns are shown in Figures f1 and f2, respectively. Figures g1 and h1 are the particle images before deuterium charging, whereas Figures g2 and h2 are the counterpart images after deuterium charging. Overall, we cannot observe stacking faults, dislocations, nor other newly introduced crystalline defects after D charging.

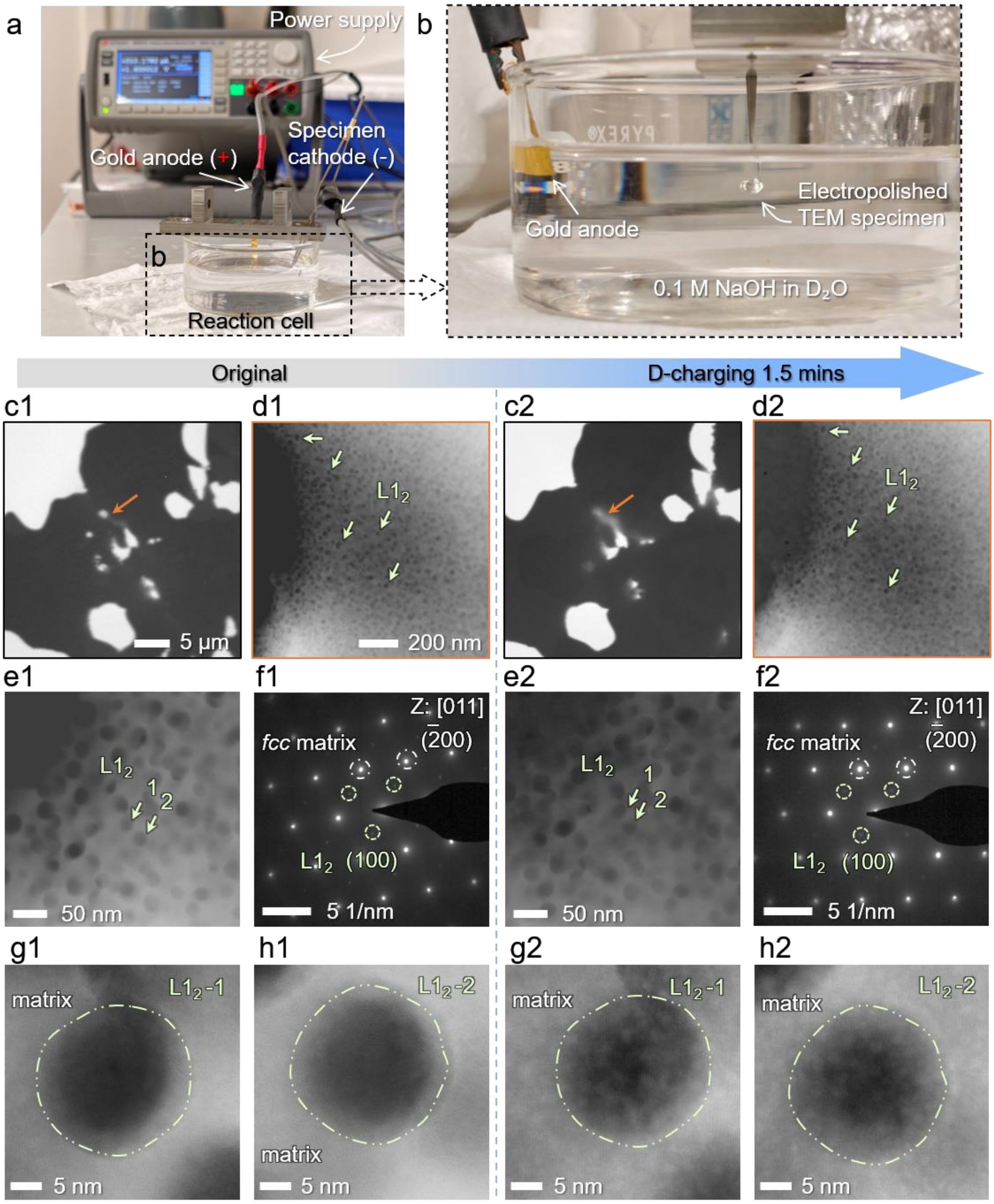

Extended Data Fig. 10 Quasi in-situ TEM observations of the same region in $(CoCrNi)_{94}Al_3Ti_3$ alloy during electrochemical D charging. a, b, Experimental setup used for quasi in-situ D charging of TEM specimens in 0.1 M NaOH prepared in D_2O. **c1, c2,** Low-magnification TEM images showing the same specimen region before charging and after D charging for 1.5 minutes. The arrow marks the same feature used for relocation. **d1, d2,** Corresponding higher-magnification HAADF-STEM images of the same area containing $L1_2$ precipitates. The same precipitates were tracked throughout the charging process. No obvious microstructural changes were observed after D charging. **e1, e2,** HAADF-STEM images showing the same $L1_2$ precipitates before charging and after D charging for 1.5 minutes. The precipitates labeled 1 and 2 were selected for repeated characterization. **f1, f2,** SAED patterns acquired along the [011] zone axis. Superlattice reflections associated with the ordered $L1_2$ structure remain visible throughout the charging process. **g1, g2** and **h1, h2,** HRTEM images of precipitates 1 and 2. No stacking faults dislocations or other newly introduced crystalline defects were detected after D charging, as confirmed by the corresponding fast Fourier transformation patterns (data not shown).

To further verify the low concentration of defect after hydrogen charging, we conducted additional positron annihilation spectroscopy experiments on a $(CoCrNi)_{94}Al_3Ti_3$ precipitate MPEA bulk specimen before and after hydrogen charging, as shown in Extended Data Figs. 11 and 12 attached below and in the revised manuscript. The positron results from uncharged/pristine state of the specimen are in black, and the counterparts from 1.5 minutes charged state of the same specimen are in blue. These two results show very little difference. We then irradiated the same specimen with Ni ions to intentionally create defects, so to verify the effectiveness of positron for the detection of defects, particularly vacancies, with such results shown in red. Overall, the positron results show no significant increase in positron recombination signal, which can be used to confirm the lack of vacancy in the pristine and hydrogen-charged specimen, contrasting to the irradiated specimen.

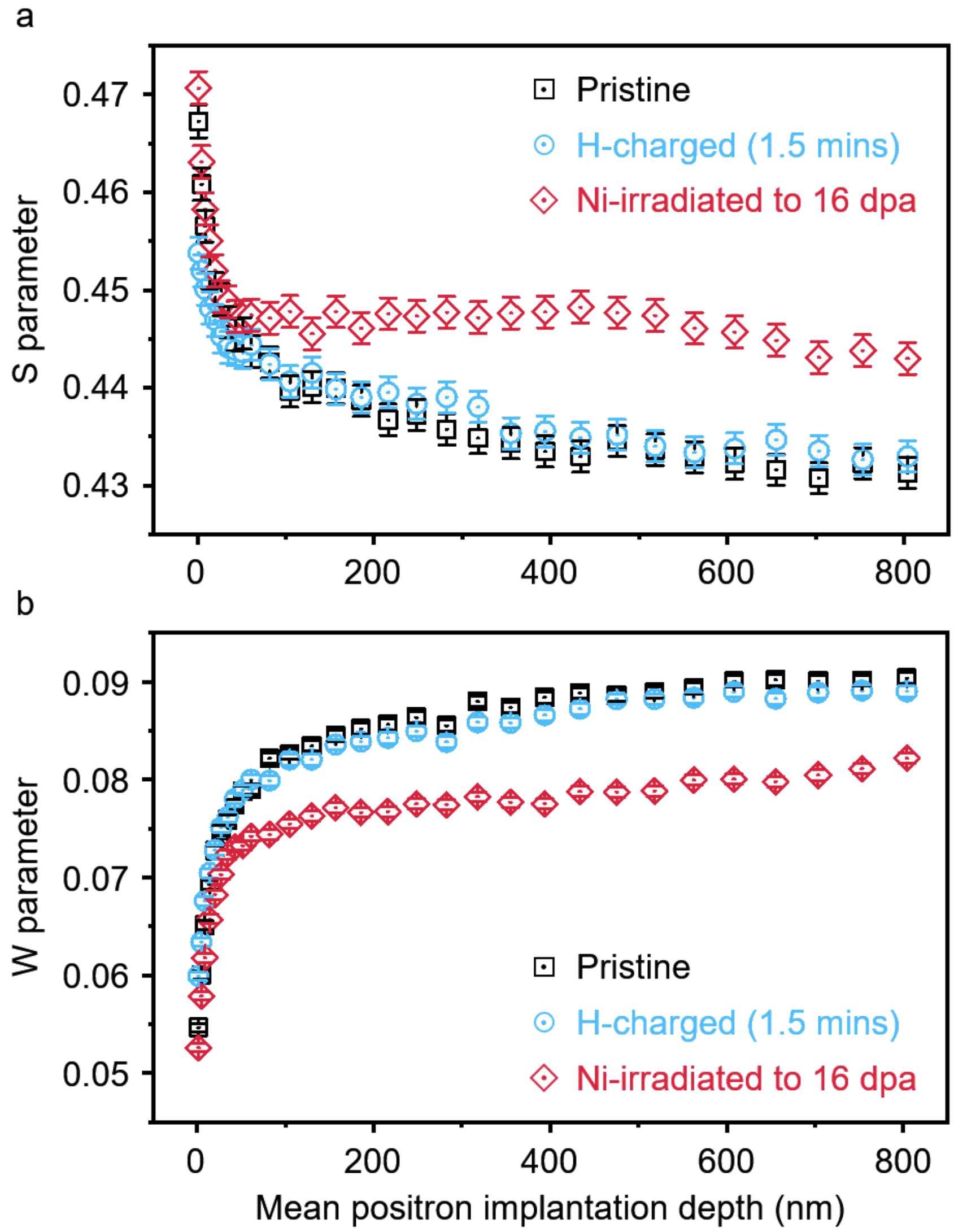

Extended Data Fig. 11 Depth-dependent Doppler-broadening positron annihilation spectroscopy results for the pristine, hydrogen-charged and Ni-ion-irradiated $(CoCrNi)_{94}Al_3Ti_3$ alloy with $L1_2$ precipitates. a, S parameter and **b,** W parameter as functions of the mean positron implantation depth. The H-charged specimen shows values close to those of the pristine specimen, indicating no detectable increase in vacancy-type open-volume defects after 1.5 minutes of H-charging process. In contrast, the specimen irradiated to 16 dpa exhibits a pronounced increase in S together with a decrease in W, indicating enhanced positron trapping at irradiation-induced vacancy-type defects.

To verify the effectiveness of irradiation for defect introduction, we conducted cross-sectional TEM by using FIB lift-out after irradiation to the specimen, as shown in Extended Data Fig. 12 attached below and in the revised manuscript. Figures a1 and a2 show the bright- and dark-field images, respectively, where the boundary between the pristine and radiation-damaged area can be seen. We analyzed the damaged region with STEM and EDS (Figure b), which contrast to the pristine region (Figure c) in defect density, i.e., the dark stripes in the bright-field image and the dark stripes in the dark-field image. We also analyzed the surface region (Figures d and e), confirming the success of defect introduction, which is consistent with the positron result in Extended Data Fig. 11.

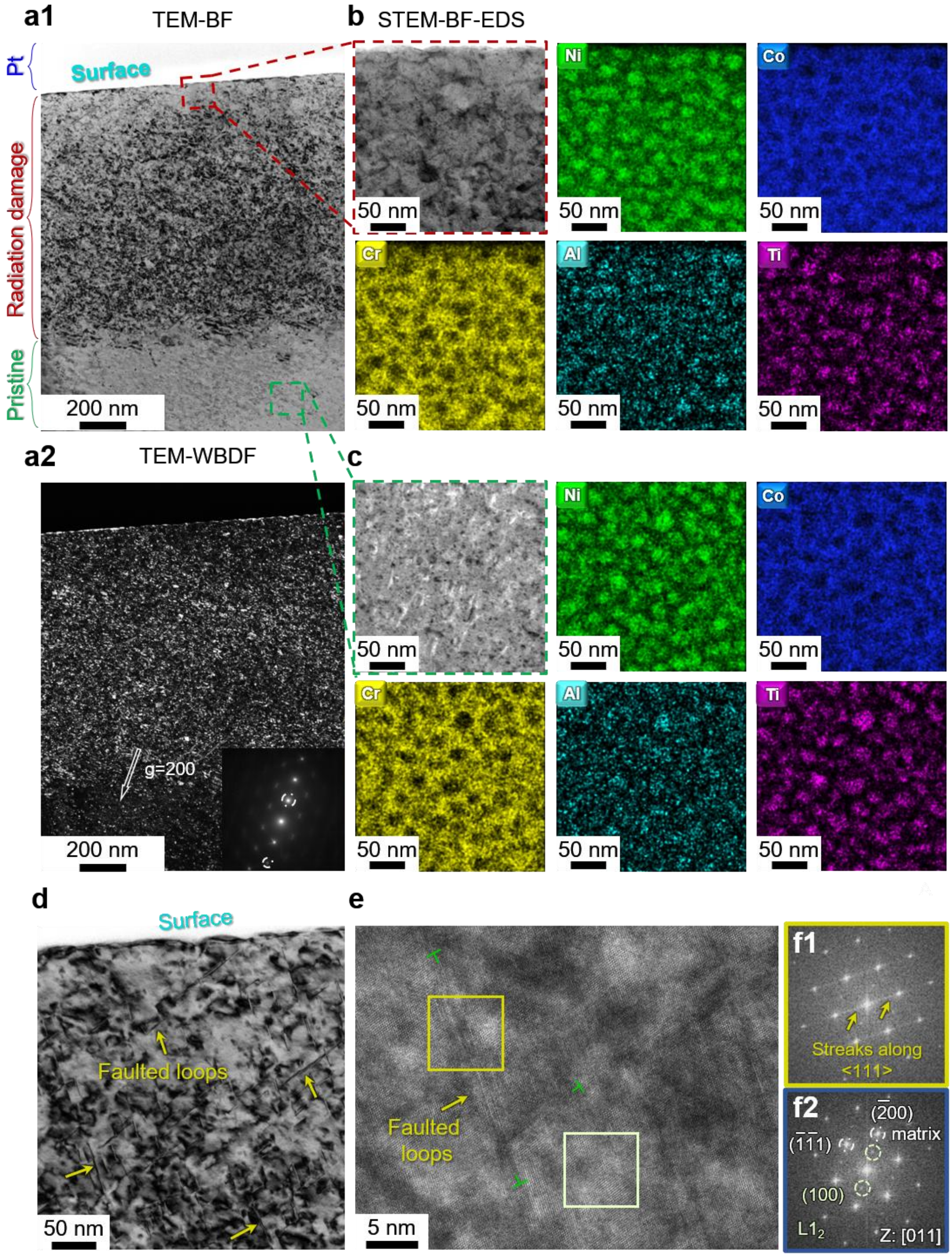

Extended Data Fig. 12 TEM characterization of irradiation-induced defects and precipitate stability after Ni-ion irradiation on a $(CoCrNi)_{94}Al_3Ti_3$ alloy with $L1_2$ precipitates. a_1, Cross-sectional two-beam

bright-field (BF) TEM image of the Ni-ion-irradiated specimen acquired under g=200, Z=[011] conditions, showing a high-density irradiation-damaged layer extending from the surface to a depth of approximately 1 μm. **a_2,** Corresponding weak-beam dark-field (WBDF) TEM image obtained under g/3g conditions, further revealing the dense distribution of irradiation-induced defects within the damaged region. **b and c,** STEM-BF images and corresponding EDS elemental maps collected from the irradiation-damaged region and the underlying pristine region, respectively. The comparable elemental partitioning between these two regions indicates that the precipitates retain their original morphology and spatial distribution after irradiation. **d,** Higher-magnification TEM-BF image of the near-surface damaged region, where numerous faulted loops are observed. **e,** High-resolution TEM image of a representative faulted loop. **f1 and f2,** FFT patterns taken from the dark yellow and pale yellow-green boxed regions and in panel (e), respectively. The streaking features along the <111> direction confirm that the dominant irradiation-induced defects are Frank-type faulted loops with Burgers vectors of b = a/3<111>. No TEM-visible vacancy clusters or voids are detected in the high-magnification images.

Taken together, these results indicate that neither pre-existing defects nor charging-induced defects are in sufficiently high densities to account for the observed D enrichment in the $L1_2$ precipitates. We therefore believe it is more appropriate to attribute the observed D enrichment to those in solution, most likely interstitially. We have summarized the new results in the revised manuscript and also softened the tone so to avoid overinterpretation. The content is now read as below.

Page 19 Line 11 to Page 20 Line 4:

We further examined whether the electrochemical D-charging process could introduce crystalline defects that potentially act as trapping sites for deuterium, as suggested in previous studies [38,41-43]. Quasi in-situ TEM observations were performed on the same $L1_2$ precipitates before and after D charging, and no stacking faults, dislocations, or other newly introduced crystalline defects were detected (Extended Data Fig. 10). In addition, as shown in Extended Data Figs. 11 and 12, positron annihilation spectroscopy, which is commonly used to characterize vacancy defects and hydrogen–vacancy complexes [44,45], showed no significant increase in vacancy concentration after D charging, contrasting to the irradiated specimen that have more defects (primarily vacancies) and thus resulted in higher positron annihilation signals.

Together, the thermodynamic analysis, DFT calculations, quasi in-situ TEM observations during D charging, and positron annihilation measurements support the interpretation that the detected D is predominantly associated with interstitial lattice sites in the $L1_2$ phase, although a minor contribution from defect-related trapping cannot be completely excluded [11,35,46-48].

Descriptions about the experiments were also added in the revised content, which read as below now.

Page 54 Line 16 to Page 55 Line 8:

Quasi in-situ TEM observation during electrochemical deuterium charging

Electropolished TEM foils of the aged $(CoCrNi)_{94}Al_3Ti_3$ alloy were used for quasi in-situ D-charging experiments. Prior to charging, specific regions containing $L1_2$ precipitates were identified and characterized

using bright-field TEM, high-angle annular dark-field scanning transmission electron microscopy (HAADF-STEM), selected-area electron diffraction (SAED), and high-resolution TEM (HRTEM). The specimen was then electrochemically charged in 0.1 M NaOH prepared in D_2O using a gold anode. Charging times of 1.5 minutes, and the charging conditions used are the same with cryogenic APT experiments.

After charging step, the specimen was removed from the electrolyte, dried, and re-examined in the TEM. The same regions and individual precipitates were relocated and compared with their former states. SAED and HRTEM analyses were performed after each charging condition to assess whether D charging introduced crystalline defects, including stacking faults, dislocations, or other lattice defects.

Page 55 Line 9 to Page 57 Line 8:

Ion irradiation and positron annihilation spectroscopy

Ion irradiation was performed using a NEC 2×1.7 MV tandem accelerator at Peking University. The $(CoCrNi)_{94}Al_3Ti_3$ specimen (10×10×0.9 mm) was irradiated with 3 MeV Ni^{2+} ions to a fluence of 1.5×10^{16} ions/cm^2 at 500 °C, corresponding to a peak damage level of approximately 16 dpa, as calculated by SRIM-2013 calculations using the Quick Kinchin–Pease model. The displacement threshold energies used in the SRIM calculations were 40 eV for Co, Cr, Ni and Ti, and 25 eV for Al. To minimize oxidation and other possible surface contamination during high-temperature irradiation, the target chamber was evacuated to a pressure better than 1×10^{-4} Pa before heating and irradiation were initiated. After irradiation, the specimen was kept under vacuum during cooling and was removed from the chamber only after the temperature had decreased below 50 °C.

Positron annihilation lifetime spectroscopy (PALS) was performed using a ^{22}Na positron source to complement the Doppler-broadening measurements and assess vacancy-type defects in the investigated alloys, corresponding to Extended Data Table 1. Vacancy-type defects introduced by hydrogen charging, performed under the same conditions as those used for the cryogenic APT experiments, and by ion irradiation were characterized by Doppler-broadening positron annihilation spectroscopy (DB-PAS) using a slow positron beam [61,62] at the Institute of High Energy Physics, Chinese Academy of Sciences. A ^{22}Na radioactive source was used to produce the positron beam, with a beam diameter of approximately 0.55 cm. To minimize specimen-to-specimen variability, DB-PAS measurements were performed sequentially on the same specimen in the pristine state, after electrochemical hydrogen charging, and after subsequent Ni-ion irradiation. The incident positron energy varied from 0.5 to 25.0 keV. The corresponding mean positron implantation depth ranged from approximately 1.5 to 804.0 nm (corresponding to the irradiated region spanning approximately 5.8–15.3 dpa), as estimated using the empirical relation:

$$Z = \frac{40}{\rho} E^{1.6} \quad \text{(Eq. 6)}$$

where Z is the mean positron implantation depth in nm, ρ is the mass density of the target material in g/cm^3, and E is the incident positron energy in keV. A density of 8.58 g/cm^3 was used for the $(CoCrNi)_{94}Al_3Ti_3$ alloy. Positron annihilation γ-ray spectra were recorded using a high-purity Ge detector. For each spectrum, more than 4.5×10^5 counts were accumulated within the 499.5–522.5 keV annihilation peak. The Doppler-broadening spectra were analyzed using the S and W parameters, which represent positron annihilation with low-momentum valence electrons and high-momentum core electrons, respectively. The annihilation peak was integrated over the energy range of 499.5–522.5 keV. The S parameter was defined as the ratio of the counts in the central energy window of 510.2–511.8 keV to the total counts in the 499.5–522.5 keV annihilation peak. The W parameter was defined as the ratio of the counts in the high-momentum wing regions of 505.1–508.4 keV and 513.6–516.9 keV to the total counts in the 499.5–522.5 keV annihilation peak. An increase in S accompanied by a decrease in W indicates enhanced positron trapping at vacancy-type open-volume defects.

3. The Conclusion section if too general and it really doesn't really capture the depth and the mechanistic aspects of these results. What are the broader implications of this work?

We appreciate the reviewer's reminder for highlighting the impact of this work. We have revised the Conclusion section with an emphasis on that the present results reveal how the atomic configuration of phases can govern the localization of interstitial hydrogen in solution. We also highlight that the new approach demonstrated here to study hydrogen in solid solution, which can provide new insight for studying hydrogen behaviors in various materials that have the potential to advance hydrogen energy. The revised content now reads as below.

Page 23 Line 20 to Page 24 Line 8:

In summary, we examined a set of FCC MPEAs, in both bulk-matrix and precipitate forms, and showed that interstitial hydrogen (represented by deuterium) can be directly probed by APT. The experimental observations agree with our DFT predictions of chemistry-dependent hydrogen solution energies. Our direct observations of interstitial hydrogen in the MPEAs further show how chemical affinity and crystal structure can influence the distribution of hydrogen in materials. These results demonstrate an experimental protocol to probe hydrogen in solid solution and develop an approach to connect atomistic predictions and experimental observation of hydrogen behavior in materials. The findings can open up new opportunities for understanding thermodynamics and, potentially, kinetics of local hydrogen to provide guidance for designing materials with compatibility and efficiency for hydrogen applications.

4. The nearest-neighbor analyses and vacancy energy calculations seem to indicate against random vacancy trapping, but what are the reasons behind this?

The nearest-neighbor (NN) analysis in APT is useful for distinguishing a dataset with significant solute atom clustering from that with only randomly distributed solute atoms. As shown in Fig. 8.19 below from Baptist Gault et al.'s book (*Atom Probe Microscopy, 2012*), for clustering solute atoms a shift towards shorter interatomic distances can be seen, such as the Sn in an aluminum alloy dataset. In contrast, for non-clustering or randomly distributed solute atoms, the distribution can result in an NN analysis result that does not fit exactly to the randomized data, as shown in the Cu NN analysis from the same aluminum alloy dataset. This is inevitable due to statistical variance rather than real solute atom clustering.

[REDACTED]

Another example of NN analysis in APT for differentiating clustering and non-clustering solute atoms by Ross Marceau et al. (*Acta Materialia, 61(19), 2013, 7285-7303.*) is shown in Fig. R1, using a AA6111 Al-Mg-Si-Cu alloy. As shown in Fig. R1a, there is a significant increase in yield strength after ageing a specimen at 180 °C for 6 hours, suggesting the presence of apparent solute clustering. The APT atom maps and NN analyses show that the specimens have either no clustering (Figs. R1b and R1c), nuanced clustering, (Fig. R1d), or apparent clustering (Fig. R1e). Note that the NN analysis results in Figs. R1b and R1c may seem like having clusters; however, neither APT reconstructions nor mechanical properties support the presence of clusters. In summary, one cannot conclude the presence of clustering with the NN analysis results shown in Figs. 1c and 1d, likewise for the additional APT data in Extended Data Figs. 3 and 4.

[REDACTED]

[redacted]

[redacted]

To improve clarity based on the reviewer's comment, we have added additional description about the data interpretation of nearest-neighbor analysis in the revised manuscript, which reads as below now.

Page 9 Line 9 to 11:

A significant shift towards shorter nearest-neighbor distances indicates deuterium clustering, whereas agreement with the random distribution is consistent with deuterium being distributed among a large population of equivalent interstitial site [34].

5. The work should also address the FIB preparation effects, which literature highlights as persistent challenges in APT hydrogen detection.

We are aware of artefacts reported by Saksena Aparna, et al. ("Optimizing site-specific specimen preparation for atom probe tomography by using hydrogen for visualizing radiation-induced damage." *International Journal of Hydrogen Energy* 50 (2024): 165-174). I.e., gallium (Ga) focused ion beam can induce vacancies and cause hydrogen/deuterium trapping as observed in their APT reconstructions. However, as indicated in the same paper by Saksena Aparna, et al. the APT specimens prepared by cryogenic Xe plasma FIB (PFIB) would not lead to such effect. All the datasets in our manuscript have adapted the best practice reported in literature, particularly FIB milling at approximately −190 °C, followed by a final 5 kV low-energy cleaning step, in order to minimize ion beam damage and thus create defect trapping sites. Details about PFIB preparation have been provided in the original manuscript, now at Page 50 Line 6 to 15 in the revised manuscript.

References for the response to Reviewer 1's comments:

1 Luo, Z., Gao, W. & Jiang, Q. Determinants of vacancy formation and migration in high-entropy alloys. Science Advances 11, eadr4697 (2025). https://doi.org/doi:10.1126/sciadv.adr4697

2 Zhao, S., Egami, T., Stocks, G. M. & Zhang, Y. Effect of d electrons on defect properties in equiatomic NiCoCr and NiCoFeCr concentrated solid solution alloys. Physical Review Materials 2, 013602 (2018). https://doi.org/10.1103/PhysRevMaterials.2.013602

3 Wang, C. et al. First-principles study of hydrogen-vacancy interactions in CoCrFeMnNi high-entropy alloy. Journal of Alloys and Compounds 922, 166259 (2022). https://doi.org/https://doi.org/10.1016/j.jallcom.2022.166259

4 Nazarov, R., Hickel, T. & Neugebauer, J. First-principles study of the thermodynamics of hydrogen-vacancy interaction in fcc iron. Physical Review B 82, 224104 (2010). https://doi.org/10.1103/PhysRevB.82.224104

5 Guan, H. et al. Chemical environment and magnetic moment effects on point defect formations in CoCrNi-based concentrated solid-solution alloys. Acta Materialia 187, 122-134 (2020). https://doi.org/https://doi.org/10.1016/j.actamat.2020.01.044

Referee #2 (Remarks to the Author):

This manuscript addresses an important and challenging question: whether atom probe tomography can reliably distinguish interstitial hydrogen in solid solution from hydrogen trapped at defects or introduced by artefacts. The work is ambitious and technically strong, with clear strengths including the use of deuterium, cryo-transfer, and a combined APT–TDS-DFT approach applied to three FCC systems of increasing complexity. The results convincingly show a phase-dependent deuterium partitioning, consistent with differences in chemical affinity between phases. However, the manuscript over-interprets these findings. While the observed partitioning is robust, it does not unambiguously demonstrate that the signal corresponds exclusively to interstitial hydrogen, as contributions from vacancies, V-Hn complexes, defects, elastic fields, local chemical heterogeneities, or kinetic effects cannot be fully excluded. The main limitation is therefore not the data quality, but the level of certainty claimed.

The experimental approach relies on three materials (CoCrNiFeMn, (CoCrNi)94Al3Ti3 with L12 precipitates, and Inconel 718 with L12 and D022), showing increasing chemical contrast. Deuterium is introduced by electrochemical charging followed by cryo-transfer, and APT reveals random distribution in the single-phase alloy and preferential partitioning in L12 phases in multiphase systems, in qualitative agreement with DFT calculations. The authors attempt to exclude vacancy trapping through thermodynamic estimates and PAS measurements, but these arguments remain insufficient, especially under non-equilibrium charging conditions.

Overall, the work is promising and potentially suitable for a journal such as Nature, but requires major revision. The authors should moderate their claims and, if possible, include additional controls addressing vacancy-related trapping, kinetic effects, H/D isotope differences, and APT uncertainties.

We thank the reviewer's positive comments. We fully agree that it is challenging to distinguish interstitial hydrogen in solution from hydrogen associated with defects, particularly vacancies that are typically "invisible" in APT datasets. As mentioned in the response to Reviewer 1's Comment 2 (Page 5 to 11 in this document), we have conducted additional experiments, including quasi in-situ TEM before and after D charging (Extended Data Fig. 10 in Page 6 of this document), showing the amount of defects generated by the D charging is insignificant, as well as multiple positron annihilation spectroscopy experiments (Extended Data Fig. 11 in Page 7 of this document) on the same specimen that was either uncharged, under the charging condition same as APT specimen, or irradiated and thus contained defects (primarily vacancies) that interacted with positron and thus demonstrated the effectiveness of the vacancy detection by positron. Additionally, APT results on other MPEA systems were also provided, showing the generality of our finding. Most importantly, according to Reviewer 2's advice, we have moderated the claims with explicit

acknowledgement to possible contributions of defects for hydrogen trapping throughout the revised manuscript. Detailed responses to Reviewer 2's comments are provided below.

Major Comments:

1) The use of three materials is well motivated and provides a clear progression from a simple system to a complex industrial alloy, with Inconel 718 offering a particularly relevant internal comparison. However, the experimental design is not orthogonal, as multiple parameters are changed simultaneously (composition, chemical disorder, precipitates, coherency, elastic fields, thermal history, and even experimental timing). As a result, the observed deuterium distribution cannot be attributed solely to chemical effects. This limitation becomes critical when claiming intrinsic chemical dependence, since microstructural and kinetic factors are also known to strongly influence hydrogen behavior in complex FCC alloys.

We appreciate the reviewer's concern about other factors that can influence the observed hydrogen distribution beyond chemical effects. The purpose of making the simple-to-complex progression is to, very excitingly, showcase the generality of our approach for studying hydrogen in solution, which is beyond the analyses of defect trapping that have been demonstrated in literature. We consider this is a major leap in not only materials characterization but also hydrogen energy materials research that is timely for the world's transition to a low-carbon society. We acknowledge that there are many other parameters that we can investigate to better address scientific interest. However, we consider a research article meant to timely report the state of the art and the foundational findings so the field can make progress altogether. We are confident our results are sufficiently sound based on the best practice we have adopted, which will inspire many researchers to perform subsequent works that can clarify other important factors in detail.

Having said that, we understand the reviewer's concerns and thus deep dive into answering whether vacancies are present in our MPEA specimens and contribute to hydrogen concentration. This is arguably the most challenging task in many studies using APT. As described in the response to Reviewer 1's Comment 2 (Page 5 to 11 in this document), we combinedly used TEM and positron annihilation to prove that the defect densities are low in bother uncharged and D-charged states of a MPEA specimen. We will elaborate the details in the following responses to other comments from the reviewer. Despite making the effort, we appreciate the reviewer's concern about overclaiming and have thus softened the claims by acknowledging the possible contribution of defects to the observed D enrichment, which follow.

Page 19 Line 11 to Page 20 Line 4:

We further examined whether the electrochemical D-charging process could introduce crystalline defects that potentially act as trapping sites for deuterium, as suggested in previous studies [38,41-43]. Quasi in-situ TEM observations were performed on the same $L1_2$ precipitates before and after D charging, and no

stacking faults, dislocations, or other newly introduced crystalline defects were detected (Extended Data Fig. 10). In addition, as shown in Extended Data Figs. 11 and 12, positron annihilation spectroscopy, which is commonly used to characterize vacancy defects and hydrogen–vacancy complexes [44,45], showed no significant increase in vacancy concentration after D charging, contrasting to the irradiated specimen that have more defects (primarily vacancies) and thus resulted in higher positron annihilation signals.

Together, the thermodynamic analysis, DFT calculations, quasi in-situ TEM observations during D charging, and positron annihilation measurements support the interpretation that the detected D is predominantly associated with interstitial lattice sites in the $L1_2$ phase, although a minor contribution from defect-related trapping cannot be completely excluded [11,35,46-48].

2) The argument excluding vacancy trapping is not fully convincing. It relies on equilibrium thermodynamics, while the experiments are performed under non-equilibrium electrochemical charging conditions known to generate superabundant vacancies and V-H_n complexes. Therefore, converting deuterium content into an equilibrium vacancy formation energy is not sufficient to rule out vacancy-related contributions. In addition, the use of the precipitation temperature is not appropriate, as relevant defects are likely formed during room-temperature charging and subsequent handling. Finally, the PAS measurements are performed on non-charged states and do not probe the actual charged condition; moreover, PAS sensitivity depends on defect chemistry, so hydrogen-decorated vacancies cannot be excluded

As raised in Reviewer 1's Comment 2, Reviewer 2's concern about the possible presence of vacancy and the inadequate reasoning in the original manuscript are well appreciated. To address this concern, as readily discussed in the response to Reviewer 1's Comment 2 in Page 5 to 11, we have gone beyond theoretical speculation and conducted additional TEM (Extended Data Fig. 10 as shown in Page 6 in this document), which confirms the defect density in specimens is low after D charging, as well as a series of positron annihilation experiment on a MPEA specimen (Extended Data Fig. 11 as shown in Page 7 in this document), which confirms the vacancy concentration (extracted from positron lifetime) at the D-charged state is as low as the pristine state, contrasting to the intentionally irradiated thus defect-rich state of the same specimen. In the revised manuscript, we have substantially strengthened the description about characterizing vacancy in specimens. More details about the positron experiments are provided as follows.

We performed Doppler-broadening positron annihilation spectroscopy (DB-PAS) measurements sequentially on the same specimen in three states: (i) pristine, (ii) electrochemically H-charged, and (iii) subsequently irradiated with 3 MeV Ni ions at 500 °C. This experimental design is important because it eliminates specimen-to-specimen variability and allows direct comparison of the charging and irradiation responses within the same material volume.

The new results (Extended Data Fig. 11 as shown in Page 7 in this document) show that the S-parameter profiles of the pristine and H-charged states remain very similar over the entire measured positron implantation depth range (approximately 1.5-804 nm), while the corresponding W-parameter profiles are likewise nearly unchanged. In contrast, the irradiated state exhibits a pronounced increase in S together with a clear decrease in W. This opposite variation of S and W is the characteristic signature of positron trapping at vacancy-type open-volume defects. The irradiated specimen therefore serves as an internal positive control, demonstrating that the present DB-PAS measurements are sufficiently sensitive to detect vacancy-type defects in this alloy system when they are present. Importantly, the depth range probed by DB-PAS substantially overlaps the irradiation-damaged region predicted by SRIM calculations. Following irradiation, the elevated S parameter and reduced W parameter are observed throughout the damaged layer, confirming that the technique readily detects irradiation-induced vacancy-related defects. If electrochemical charging had generated a substantial population of superabundant vacancies or $V–H_n/V–D_n$ complexes sufficient to account for the deuterium concentrations observed by APT, a comparable deviation in the S/W response would be expected when comparing the pristine and charged states under identical measurement conditions. Such a response is not observed.

We agree with the reviewer that hydrogen-decorated vacancies do not necessarily exhibit the same positron signatures as bare vacancies, and therefore DB-PAS cannot strictly prove the complete absence of all H-vacancy complexes. However, the critical point here is quantitative rather than absolute. The positron measurements show that hydrogen charging does not introduce a detectable population of vacancy-type open-volume defects comparable to that generated by irradiation. Therefore, while a small number of hydrogen-decorated vacancies below the detection limit cannot be completely excluded, the PAS data rules out a substantial charging-induced vacancy population and therefore argue strongly against vacancy trapping being the dominant origin of the deuterium signal detected by APT.

To further support this interpretation, we performed TEM characterization of the irradiated specimen, as shown in Extended Data Fig. 12 in Page 8 in this document. The irradiated layer contains a high density of irradiation-induced faulted loops, confirming the introduction of irradiation damage. Notably, no TEM-visible voids or vacancy clusters were observed. Furthermore, STEM-EDS maps acquired from both the irradiated region and the underlying unirradiated region reveal little change in Ni, Co, Cr, Al and Ti partitioning, indicating the morphology and chemistry of the $L1_2$ precipitates remain stable after irradiation. Consequently, the PAS response observed after irradiation cannot be attributed to precipitate coarsening, dissolution, or compositional redistribution, but instead reflects irradiation-induced vacancy-type defects.

Taken together, the revised evidence forms a consistent non-equilibrium experimental framework. First, H charging produces no measurable increase in the S parameter and no measurable decrease in the W

parameter relative to the pristine state. Second, irradiation of the same specimen produces the expected strong PAS response, validating the sensitivity of the technique to vacancy-type defects in this alloy. Third, TEM confirms irradiation-induced defect formation while excluding the presence of TEM-visible vacancy clusters or significant precipitate evolution. Finally, the phase-selective deuterium enrichment observed by APT and the independently predicted hydrogen affinity of the $L1_2$ phase remain fully consistent with interstitial hydrogen partitioning. We therefore believe that the detected deuterium is predominantly associated with interstitial lattice sites rather than charging-induced vacancy trapping, although a minor contribution from defect-related traps below the detection limit of the present techniques cannot be completely excluded. Note that the method description is available in Page 55 in the revised manuscript.

Regarding hydrogen-decorated vacancies cannot be completely excluded, we agree with the reviewer's view. Therefore, we have moderated the corresponding discussion and now conclude that the detected deuterium is predominantly associated with interstitial lattice sites, while a minor contribution from defect-related trapping cannot be entirely ruled out. The revised content reads as follows.

Page 19 Line 11 to Page 20 Line 4:

We further examined whether the electrochemical D-charging process could introduce crystalline defects that potentially act as trapping sites for deuterium, as suggested in previous studies [38,41-43]. Quasi in-situ TEM observations were performed on the same $L1_2$ precipitates before and after D charging, and no stacking faults, dislocations, or other newly introduced crystalline defects were detected (Extended Data Fig. 10). In addition, positron annihilation spectroscopy, which is commonly used to characterize vacancy defects and hydrogen–vacancy complexes [44,45], showed no detectable increase in vacancy concentration after D charging (Extended Data Fig. 11), whereas irradiated specimens used as positive controls exhibited clear vacancy-related signals (Extended Data Figs. 11 and 12).

Together, the thermodynamic analysis, DFT calculations, quasi in-situ TEM observations during D charging, and positron annihilation measurements support the interpretation that the detected D is predominantly associated with interstitial lattice sites in the $L1_2$ phase, although a minor contribution from defect-related trapping cannot be completely excluded [11,35,46-48].

3) The protocol introduces a significant kinetic bias. The delay before quenching allows diffusion and preferential desorption, meaning the measured distribution is not a direct snapshot after charging. As shown in recent cryo-APT studies, such short thermal pauses can affect hydrogen redistribution, so the results likely reflect a combination of thermodynamic and kinetic effects.

We thank the reviewer for pointing out the exact highlight of our experimental design. Given the deuterium distributions measured by APT must be a combination of thermodynamic and kinetic effects, how to let D

atoms redistribute into a relatively steady state without losing them completely is the very challenge in this study. Note that before this work no one can really observe hydrogen in solution. As readily described in the original manuscript, now highlighted in Page 6, Line 14 in the revised manuscript, the results in Fig. 1 (and Extended Data Figs. 3 and 4 in Pages 3 and 4 in this document) are from our best practice of cryogenic transfer, allowing only approximately 3 seconds between D charging and quenching to preserve as much D content as possible. We consider this approach as "immediately" quenching after D charging given it is physically impossible to spend no time for spraying off the residual heavy water from the electrochemical charging before quenching. The results in Fig. 1 and Extended Data Figs. 3 and 4 thus serve as a showcase of the best extent of D atom preservation in the MPEA matrices.

Moving on, the results in Fig. 3 reflect the D distribution after 10 seconds at room temperature. Namely they reflect the state of being partially desorbed and more stabilized relative to that of Fig. 1. Fig. 4 used the same protocol for Fig 3, as readily described in the original manuscript. Our results indeed reflect the kinetics of hydrogen redistribution to a certain extent, which is very difficult to achieve in other laboratories which have less experienced in conducting cryo-APT experiments than us. In summary, the combination of Fig. 1 (and Extended Data Figs. 1, 3 and 4) with Fig. 3 (and Extended Data Fig. 8) and Fig. 4 (and Extended Data Fig. 15) beautifully present the kinetics of hydrogen redistribution, and we are very proud to present this kinetic "bias" as the reviewer points out.

4) The manuscript largely neglects the role of SFE, although it is central in FCC alloys. Hydrogen is known to modify SFE and interact strongly with dislocations and planar defects. In HEA/MEA systems, local chemical order produces a wide distribution of fault energies and defect structures. Therefore, part of the observed deuterium distribution may be associated with defects rather than purely interstitial solution.

The change of stacking fault energy (SFE) due to hydrogen charging is indeed reported for literature about bulk FCC MPEA systems. However, to our knowledge, there has been very little literature reporting the similar effect to the $L1_2$ phase. Namely, we are not sure whether the presence of hydrogen can lead to an increase in defect density within the $L1_2$ phase. As such, we performed an additional quasi in-situ TEM observation on the same $L1_2$ precipitates before and after D charging for 1.5 minutes, as shown in Extended Data Fig. 10 in Page 6 in this document. Our selected area diffraction and high-resolution TEM analyses did not reveal the formation of stacking faults, planar defects or other newly introduced crystalline defects after D charging. These results suggest that, under the charging conditions employed in this work, defect structures associated with SFE modification are not sufficiently abundant to account for the observed deuterium enrichment. More methodological details are provided in the revised manuscript from Page 54 Line 16 to Page 55 Line 8.

Having said that, we agree that local variations in SFE and defect energetics may contribute to hydrogen behavior in complex FCC alloys. We have thus moderated the corresponding discussion and avoid attributing the observed deuterium distributions exclusively to a single controlling factor. Revised discussion about defect-related contributions in manuscript is provided in our response to Reviewer 1's Comment 2 and in the revised manuscript from Page 19 Line 11 to Page 20 Line 4.

5) The enrichment of deuterium in L12 precipitates is interpreted as a chemical effect. However, coherent precipitates generate elastic fields due to lattice misfit. Hydrogen solubility is known to depend on hydrostatic stress. Therefore, the observed partitioning is likely a combined chemical-elastic effect, not purely chemical.

We agree that coherent precipitates generate elastic fields and that hydrogen solubility can be influenced by hydrostatic stress. However, we do not consider elastic fields to be the dominant factor in the present observations. This is because our observation (Fig. 3) shows the highest deuterium concentrations at the interior of the $L1_2$ precipitates rather than at the coherent precipitate–matrix interfaces where elastic effects are stronger, suggesting that elastic effects alone are unlikely to account for the measured deuterium distributions. Nevertheless, we agree that elastic effects cannot be completely excluded and have revised the manuscript accordingly to avoid attributing the observed behavior exclusively to chemical effects. We have thus moderated the discussion throughout the manuscript and now state that the detected D is predominantly associated with interstitial lattice sites, while a minor contribution from defect-related trapping cannot be completely excluded. Revised discussion about defect-related contributions in manuscript is provided in our response to Reviewer 1's Comment 2 and in the revised manuscript from Page 19 Line 11 to Page 20 Line 4.

6) The DFT approach captures average trends but does not fully represent the broad distribution of interstitial site energies expected in chemically complex alloys. In addition, the calculations are limited to ideal interstitial configurations and do not account for competing states such as vacancy–hydrogen complexes or defect-associated trapping. As a result, the interpretation in terms of a single solution energy per phase is likely oversimplified, and the DFT results should be viewed as indicative trends rather than a complete description of hydrogen behavior.

We appreciate the valuable comments and have done a more thorough set of DFT calculations to develop the heterogeneity of hydrogen location to address reviewer's concern. In particular, we extended the results presented in the Extended Data Fig. 2, as shown in the next page, with a range of hydrogen solution energies at either octahedral or tetrahedral interstitial sites (noted as Oct and Tet in the figure and hereafter). Note that the black stars provide the averages of the solution energies for different interstitial sites. In Extended Data

Fig. 2, we found the overall comparative tendency is similar to the results presented in the original manuscript. Owing to the local chemical disorder, the solution energy values for hydrogen in the MPEAs are indeed distributed widely with respect to that in pure Ni and D0$_{22}$ Ni_3Nb. For CoCrNiFeMn (Extended Data Fig. 2a), CoCrNi (Extended Data Fig. 2b), L1$_2$ Ni_3(Al,Ti) (Extended Data Fig. 2b), pure Ni (Extended Data Fig. 2c), and Ni_2FeCr (Extended Data Fig. 2d), the average solution energies of hydrogen at Oct sites are generally lower than the hydrogen at Tet sites, which means the Oct interstitial site is indeed the preferential occupancy sites for these alloys. The only exception is when at the interface structure (Extended Data Fig. 2b) the Tet sites are more easily occupied interstitial. These findings are in good agreement with other FCC MPEAs literature [1,2].

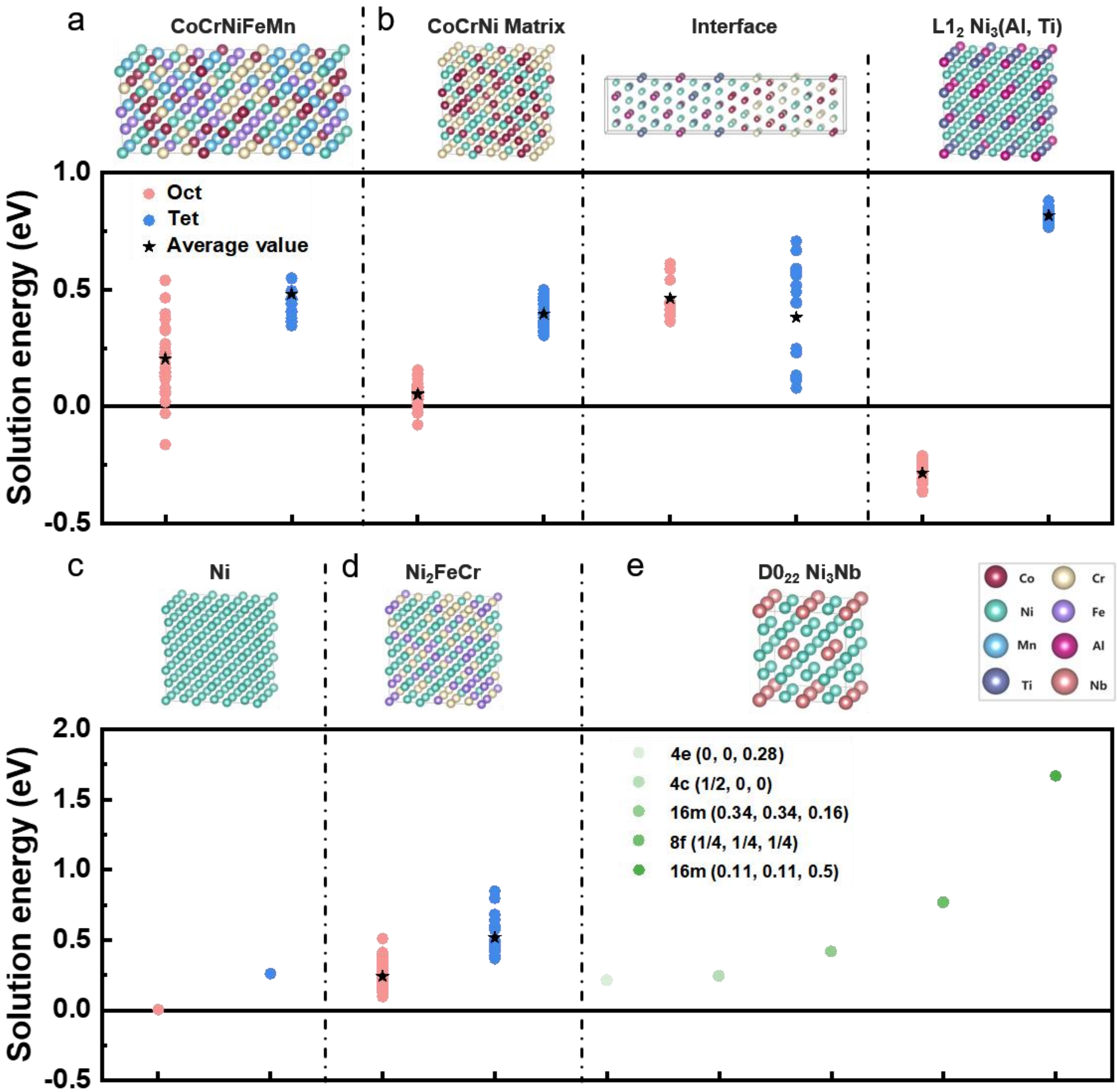

Extended Data Fig. 2 Density Function Theory (DFT) calculations on hydrogen solution energies at

various interstitial sites. a, Atomistic structure used in calculation and hydrogen solution energies at the octahedral interstitial sites and tetrahedral interstitial sites in the bulk of CoCrNiFeMn FCC matrix. **b,** Atomistic structure and hydrogen solution energies at octahedral and tetrahedral in the CoCrNi FCC matrix, CoCrNi $(1\bar{1}\bar{1})$/L1$_2$ Ni_3(Al, Ti) $(1\bar{1}\bar{1})$ interface, and L1$_2$ Ni_3(Al, Ti) structure. A subset of octahedral sites that are not energetically favorable for hydrogen solution is excluded. **c,** Atomistic structure and hydrogen solution energies at octahedral and tetrahedral in the Ni FCC matrix. **d,** Atomistic structure and hydrogen solution energies at octahedral and tetrahedral in the Ni_2FeCr FCC matrix. **e,** Atomistic structure and hydrogen solution energies at octahedral and tetrahedral in the D0$_{22}$ Ni_3Nb structure.

Regarding D0$_{22}$ Ni_3Nb (Extended Data Fig. 2e), the lowest hydrogen solution energy is at the 4e interstitial site. Overall, the L1$_2$ Ni_3(Al, Ti) remains having the lowest hydrogen solution energy, which is consistent with our APT observations. As the reviewer points out, in this work we only consider DFT results as indicative trends and would like to highlight the APT results. We have thus revised the relevant content in the revised manuscript, which is now read as follows.

Page 14 Line 8 to 13:

Owing to the chemical disorder, the solution energy values for hydrogen in the MPEAs are distributed widely with respect to that in pure metals. We find that hydrogen exhibits the lowest solution energy (approximately −0.28 eV) in the specific octahedral sites of the L1$_2$ Ni_3(Al, Ti) phase. These complementary experimental and computational observations motivated a microscopic APT study to further investigate hydrogen distribution at the nanoscale.

Regarding the reviewer's concern about defects, particularly vacancy, we performed additional calculations regarding the vacancy. We selected three representative phases (CoCrNi, L1$_2$ Ni_3(Al, Ti), and D0$_{22}$ Ni_3Nb) to conduct the relevant investigations, which can reflect the general energetic characteristics. Vacancies were introduced by removing atoms from the perfect supercell, the vacancy formation energy E_V^f was computed as follows:

$$E_V^f = E_V - E_{bulk} + \mu_i \qquad \text{(Eq. 1)}$$

where E_V and E_{bulk} are the energies of defective and defect-free supercells, μ_i is the chemical potential of the removed atom from the supercell. The chemical potential of a specific atom is defined as the energy change of the total free energy per atom, which is highly dependent on the metallic bonding around the species. In pure metals or conventional dilute alloys, the chemical potential is usually considered as the energy of each atom in the bulk. However, in multicomponent MPEAs, the chemical potential is different from that in pure reference states. The classical Widom-type substitution technique has been applied to calculate the elemental chemical potentials in MPEAs [3].

For CoCrNi system consisting of three components, the difference in chemical potential between the Co

element (μ_{Co}^{CoCrNi}) and the Cr element (μ_{Cr}^{CoCrNi}) in CoCrNi can be obtained by Eq. 2.

$$\mu_{Co}^{CoCrNi} - \mu_{Cr}^{CoCrNi} = \frac{1}{2}(\Delta\mu_{Cr\to Co} - \Delta\mu_{Co\to Cr}) \quad \text{(Eq. 2)}$$

where $\Delta\mu_{Cr\to Co}$ and $\Delta\mu_{Co\to Cr}$ are the average substitution energies of the Cr atom replaced by the Co atom and the Co atom replaced by the Cr atom, respectively.

Similarly, we can obtain the difference in chemical potential between Co and Ni.

$$\mu_{Co}^{CoCrNi} - \mu_{Ni}^{CoCrNi} = \frac{1}{2}(\Delta\mu_{Ni\to Co} - \Delta\mu_{Co\to Ni}) \quad \text{(Eq. 3)}$$

The total energy of the system is:

$$E_{bulk} = \sum_i N_i\, \mu_i^{CoCrNi} \quad \text{(Eq. 4)}$$

where N_i and μ_i^{CoCrNi} are the number and chemical potential of element *i* in a perfect supercell. We calculated 40 substituted structures to determine the μ_i of each element *i*. The calculated chemical potentials of different elements in CoCrNi and pure metals are shown in Table R1. It shows that the chemical potential for Co and Ni are close to that in the corresponding pure metal, and the chemical potential for Cr in CoCrNi is a little higher than that in the pure Cr. These chemical potentials were used in Eq. (1) to calculate the formation energies of vacancies in CoCrNi. For $L1_2$ $Ni_3(Al, Ti)$ and $D0_{22}$ Ni_3Nb, μ_i denotes the total energy of X-atom at its ground state.

Table R1 Chemical potentials of constituent elements in CoCrNi and pure metals (eV/atom).

	CoCrNi		Pure metal	
	**current work**	**Ref.** [4]	**current work**	**Refs.**
μ_{Co}	−6.982	−6.97 ± 0.03	−7.035	−6.83 [5]
μ_{Cr}	−9.297	−9.27 ± 0.06	−9.530	−9.49 [5]
μ_{Ni}	−5.498	−5.50 ± 0.02	−5.470	−5.42 [5]
μ_{Al}	/		−3.745	−3.74 [3]
μ_{Ti}	/		−7.802	−8.10 [3]

Vacancies can serve as trapping centers for dissolved hydrogen [6]. Extended Data Fig. 9 in the next page as well as Page 33 in the revised manuscript shows the calculated vacancy formation energies in CoCrNi and $L1_2$ $Ni_3(Al, Ti)$. More than 100 values of vacancy formation energy were computed for each alloy.

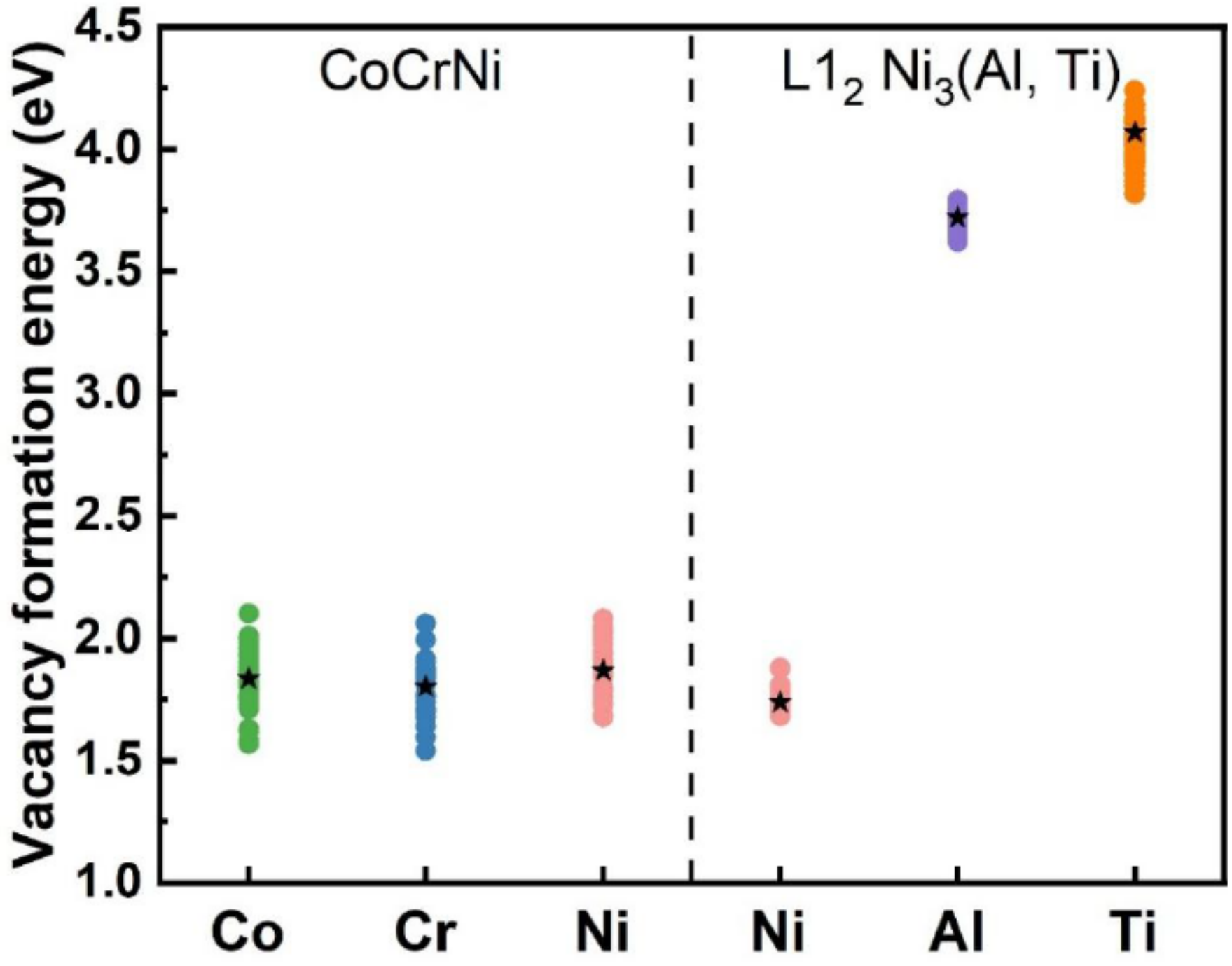

Extended Data Fig. 9 Vacancy formation energies in CoCrNi and L1$_2$ Ni_3(Al, Ti).

Table R2 presents the average formation energies of different elements in CoCrNi, L1$_2$ Ni_3(Al, Ti), and D0$_{22}$ Ni_3Nb. The results indicate that the vacancy formation energies in L1$_2$ Ni_3(Al, Ti) are generally higher than those in CoCrNi. In CoCrNi, the vacancy formation energy of Cr is relatively lower than those of Co and Ni. In L1$_2$ Ni_3(Al, Ti), Ni exhibits the lowest vacancy formation energy (1.739 eV), which is still considerably high. In D0$_{22}$ Ni_3Nb, the vacancy formation energy of Ni is lower than that of Nb. Notably, the vacancy formation energies in L1$_2$ Ni_3(Al, Ti) are much higher than those in CoCrNi, especially for Al and Ti, suggesting that it is difficult to form vacancies in L1$_2$ Ni_3(Al, Ti). The relevant contents in the revised manuscript are now read as follows.

Page 18 Line 13 to 15:

Moreover, our DFT calculations show that the vacancy formation energies in CoCrNi and L1$_2$ Ni_3(Al, Ti) are greater than 1.5 eV (Extended Data Fig. 9).

Table R2 The vacancy formation energies in CoCrNi, L1$_2$ Ni_3(Al, Ti), and D0$_{22}$ Ni_3Nb (eV).

	**CoCrNi**		**L1$_2$ Ni_3(Al, Ti)**	**D0$_{22}$ Ni_3Nb**
	**Current work**	**Ref. [7]**		
Co	1.846	1.837	-	-
Cr	1.804	1.776	-	-
Ni	1.879	1.845	1.739	1.526
Al	-		3.721	-
Ti	-		4.007	-
Nb	-		-	3.401

Combining the above DFT calculations with thermodynamic analysis, quasi in-situ TEM observations

during D charging, and positron annihilation measurements above, it is verified that appreciable vacancy concentrations are difficult to form in the CoCrNi and $L1_2$ $Ni_3(Al, Ti)$ systems. Instead, the detected D is predominantly associated with interstitial lattice sites in the $L1_2$ $Ni_3(Al, Ti)$ phase. The relevant contents in the revised manuscript are now read as follows.

Page 19 Line 21 to Page 20 Line 4:

Together, the thermodynamic analysis, DFT calculations, quasi in-situ TEM observations during D charging, and positron annihilation measurements support the interpretation that the detected D is predominantly associated with interstitial lattice sites in the $L1_2$ phase, although a minor contribution from defect-related trapping cannot be completely excluded [11,35,46-48].

7) DFT calculations are performed at 0K, without ZPE, entropy, or defect contributions. They describe ideal interstitial solutions, not the full competition between possible hydrogen states.

We appreciate the reviewer's view and thus have performed ZPE and entropy corrections on the solution energy for three selected phases, CoCrNi, $L1_2$ $Ni_3(Al, Ti)$ and $D0_{22}$ Ni_3Nb. The results are illustrated in Extended Data Fig. 19 as shown in the next page and Page 44 in the revised manuscript. The Gibbs free energy of hydrogen dissolution, ΔG_s is defined as follows:

$$\Delta G_s = E_s + \Delta E_{ZPE} - T\Delta S \qquad \text{(Eq. 5)}$$

where E_s is the calculated solution energy, ΔE_{ZPE} is the difference in zero-point energy of hydrogen in the dissolved state and the gas phase, and ΔS is the entropy difference between the dissolved state and the gas phase of hydrogen at standard conditions (atmospheric pressure, 298K). The ZPE of H atoms can be calculated by summing up the vibrational energies of the normal modes by the expression:

$$ZPE = \frac{1}{2}\sum_i hv_i \qquad \text{(Eq. 6)}$$

where h and v_i are Plank's constant and the normal vibration frequencies. Atomic vibrational frequency is proportional to the inverse square-root of atomic mass. The ZPE of H atoms in all alloys was calculated using Einstein approximation, where the vibrational energies of metal atoms are assumed to be much smaller than that of H atoms.

Therefore, metal atoms were fixed and the ZPE is calculated only from normal vibrational modes of H atoms. The calculated ZPE of a diatomic H_2 gas is 0.269 eV, which is in well agreement with others [8-10].

Extended Data Fig. 19 illustrates the comparison of energy results with and without ZPE and entropy corrections. The energy slightly increases after ZPE correction is applied, and the magnitude of the increase of tet sites is marginally larger than the oct sites for CoCrNi and $Ni_3(Al, Ti)$. The energy rises further with additional entropy corrections for all the three alloys. It can be seen that the ZPE and entropy corrections exert no significant influence on the energy comparison.

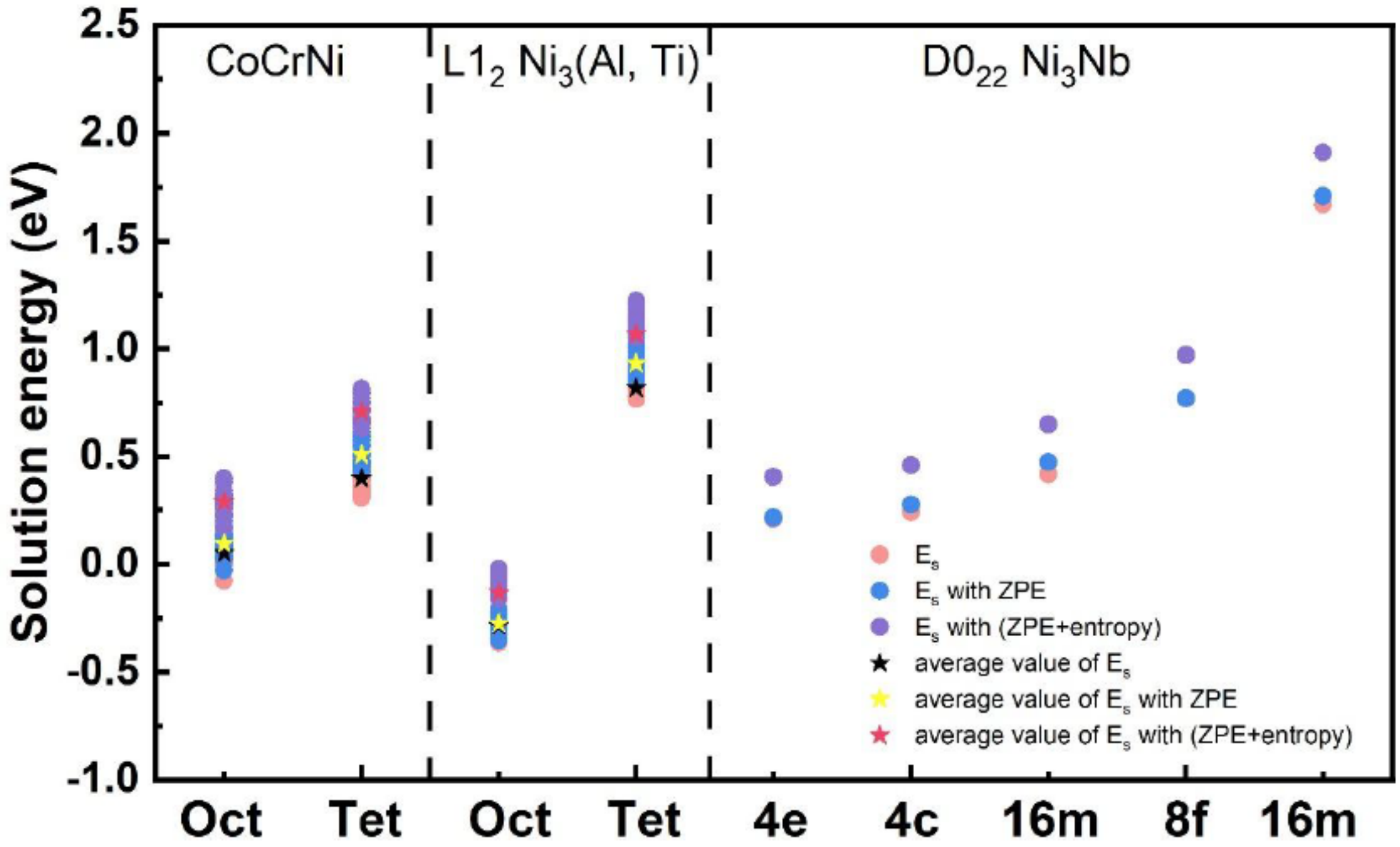

Extended Data Fig. 19 Solution energy results with and without ZPE and entropy corrections.

To address the reviewer's view, the relevant contents in the revised manuscript are now read as follows. Page 53 Line 11 to Page 54 Line 6:

According to quantum mechanics, particles maintain a residual nonzero energy even at 0 K, which is defined as zero-point energy (ZPE) [58]. Here, we employed the ZPE and entropy corrections on H solution energies for three of the investigated alloys (CoCrNi, L1$_2$ Ni_3(Al, Ti) and D0$_{22}$ Ni_3Nb). The Gibbs free energy of hydrogen dissolution, ΔG_s is defined as follows:

$$\Delta G_s = E_s + \Delta E_{ZPE} - T\Delta S \qquad \text{(Eq. 5)}$$

E_s is the calculated solution energy, ΔE_{ZPE} is the difference in zero-point energy of hydrogen in the dissolved state and the gas phase, and ΔS is the entropy difference between the dissolved state and the gas phase of hydrogen at standard conditions (atmospheric pressure, 298K). The calculated ZPE of a diatomic H_2 gas is 0.269 eV, which is in good agreement with others [58-60]. Extended Data Fig. 19 illustrates the comparison of energy results with and without ZPE and entropy corrections. The energy slightly increases after ZPE correction is applied, and the magnitude of the increase of tet sites is marginally larger than the oct sites for CoCrNi and Ni_3(Al, Ti). The energy rises further with additional entropy corrections for all the three alloys. It can be seen that the ZPE and entropy corrections exert no significant influence on the energy comparison.

8) The use of deuterium is appropriate, but isotopic effects (ZPE, diffusivity, binding energies) are not considered in calculations. This limits strict quantitative comparison

We thank the reviewer pointing this out and have supplemented the calculations of D solution energies in

three alloys (CoCrNi, L1$_2$ Ni$_3$(Al, Ti), and D0$_{22}$ Ni$_3$Nb), and ZPE corrections were also incorporated. The calculated results were shown in Extended Data Fig. 20. As can be observed in the figure, replacing H with D exerts negligible influence on the solution energies for all the three phases. The energy slightly increases with the ZPE correction, which is similar to the case of H. The general trend of energy comparison is insensitive to isotopic effects.

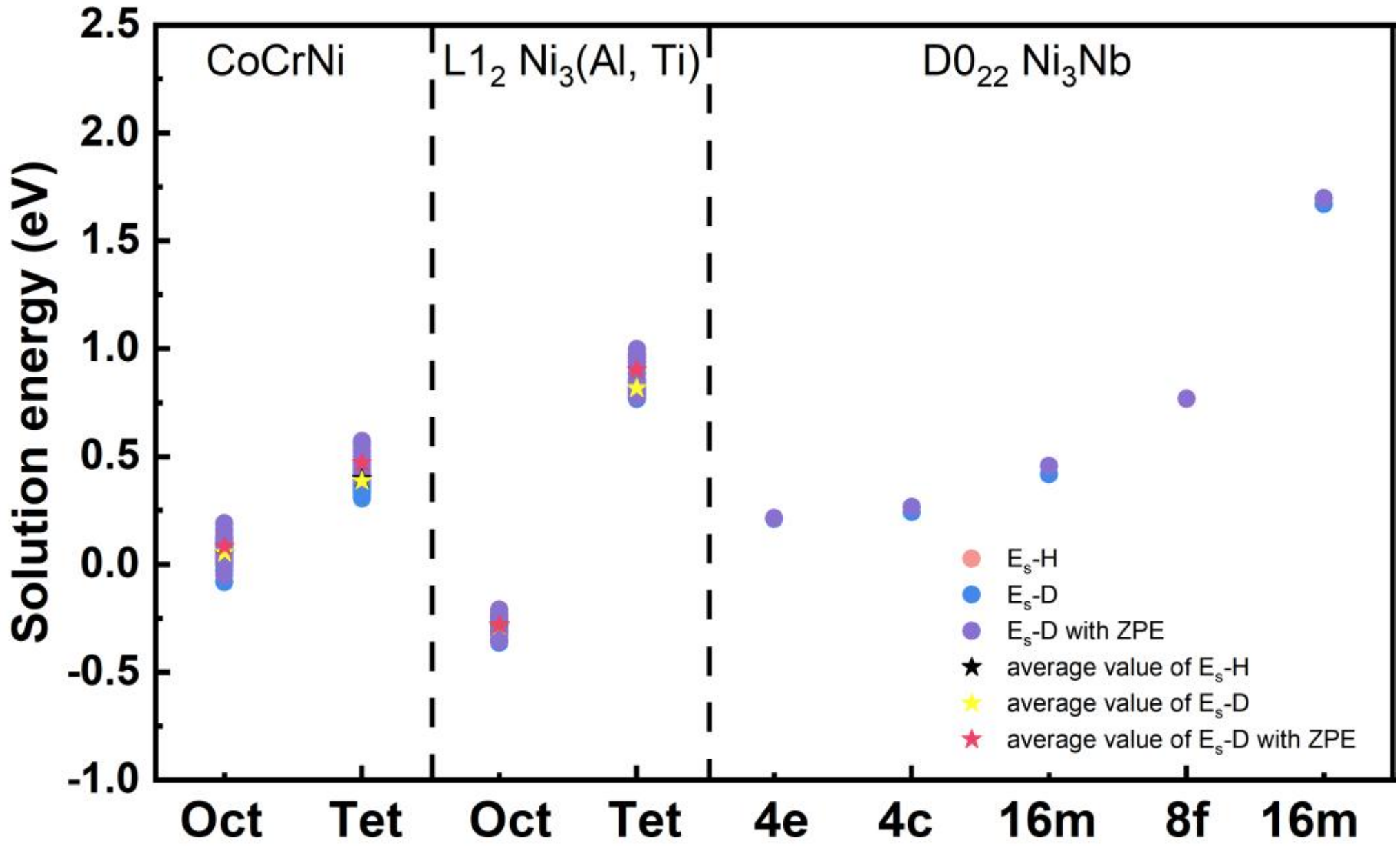

Extended Data Fig. 20 Energy results of isotopic effects in CoCrNi, L1$_2$ Ni$_3$(Al, Ti), and D0$_{22}$ Ni$_3$Nb.

The diffusion of H/D atoms within the three phases (CoCrNi, L1$_2$ Ni$_3$(Al, Ti), and D0$_{22}$ Ni$_3$Nb) was investigated employing the climbing-image nudged elastic band method. As shown in Extended Data Fig. 21, the diffusion barriers of H from oct to oct in CoCrNi and L1$_2$ Ni$_3$(Al, Ti) are 0.48 eV and 0.88, respectively. The diffusion barrier of H from 4e to 16m in D0$_{22}$ Ni$_3$Nb is 0.42 eV. The diffusion barriers of H and D in three phases are similar to each other.

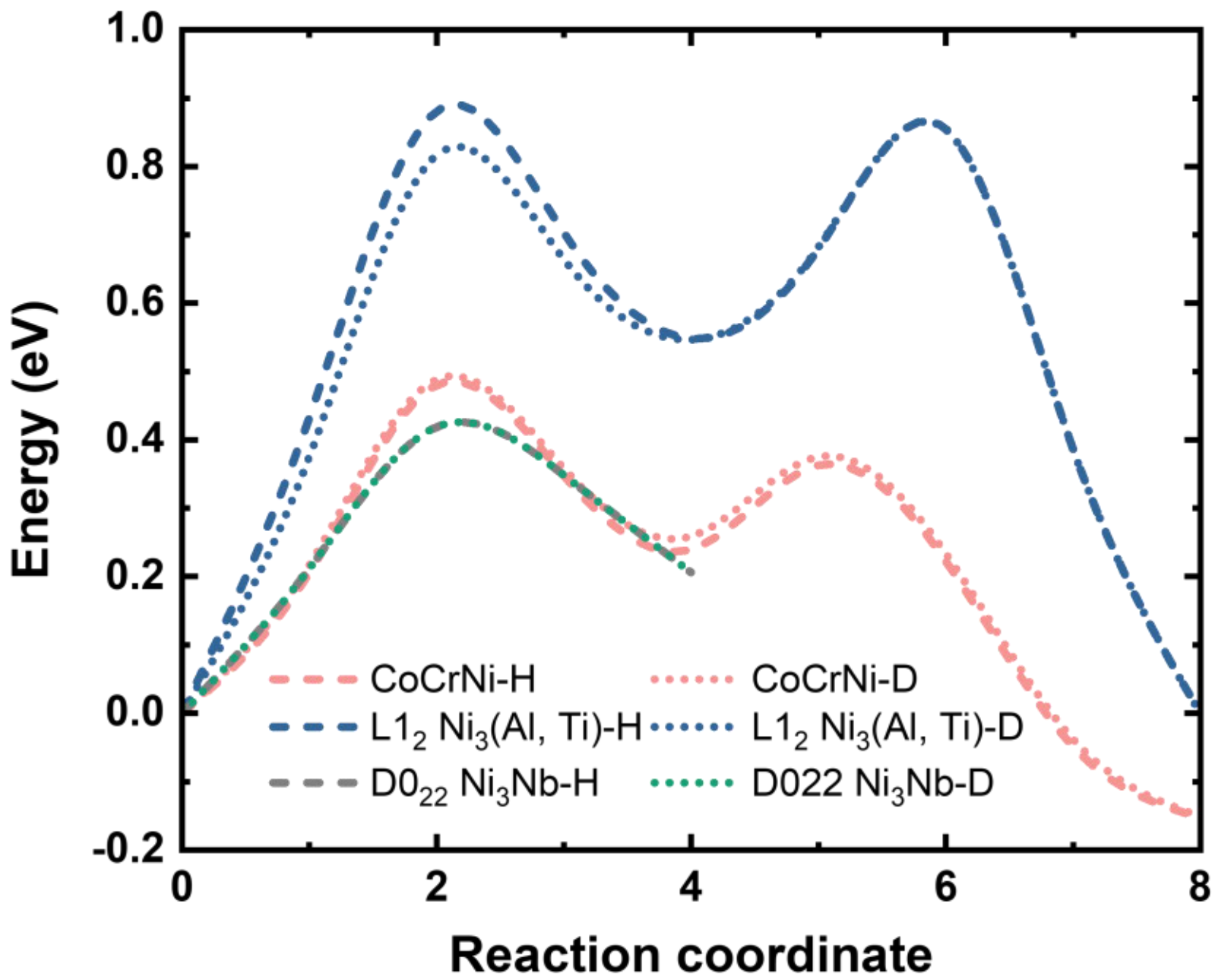

Extended Data Fig. 21 Hydrogen/Deuterium diffusion pathways in in CoCrNi, L1$_2$ Ni$_3$(Al, Ti), and D0$_{22}$ Ni$_3$Nb.

The relevant part in the revised manuscript is now as below.

Page 54 Line 7 to 15:

Isotopic effect was also considered for these alloys (CoCrNi, $L1_2$ $Ni_3(Al, Ti)$ and $D0_{22}$ Ni_3Nb). H atom was substituted by D atom, the calculated results were shown in Extended Data Fig. 20. Replacing H with D exerts negligible influence on the solution energies for all three phases. The energy slightly increases with the ZPE correction, which is similar to the case of H. However, the general trend of energy comparison is insensitive to isotopic effects. The diffusion of H/D atoms within the three phases (CoCrNi, $L1_2$ $Ni_3(Al, Ti)$, and $D0_{22}$ Ni_3Nb) was investigated employing the climbing-image nudged elastic band method. As shown in Extended Data Fig. 21, the diffusion barriers of H and D in three phases are similar to each other.

9) APT quantification needs stronger support. Although best practices are followed, key sources of uncertainty (mass overlaps, background signal, and reconstruction sensitivity) are not fully documented, which limits the strength of the conclusions.

We appreciate the reviewer's advice on incorporating description on APT-related uncertainties and have added additional contents in the revised manuscript, mainly in the Extended Data and Methods sections.

We first assessed the possible mass-overlap effects and the reliability of peak ranging and performed bulk composition analyses for representative datasets from the CoCrFeMnNi alloy, the $(CoCrNi)_{94}Al_3Ti_3$ alloy and the Inconel 718 nickel-based superalloy in main text. The quantified major-element compositions are consistent with the designed alloy compositions as shown in Extended Data Tables 2, 3 and 4 as shown below and in Page 41 in the revised manuscript. The data supports the reliability of the peak assignments used in this work.

Extended Data Table 2 Bulk composition of CoCrFeMnNi APT dataset

Ion Type	Ion Count	Ionic Fraction (%)	Ionic Fraction (sigma) (%)
**Ni**	7981600	22.54	0.0088
**Mn**	6997940	19.76	0.0082
**Fe**	6914580	19.53	0.0081
**Cr**	6534180	18.45	0.0079
**Co**	6228280	17.59	0.0076
**D**	510975	1.44	0.0020
**Si**	99307	0.28	0.00089
**H**	97449	0.28	0.00088
**C**	21901	0.062	0.00042
**P**	10030	0.028	0.00028
**Noise**	7969	0.023	0.00025

Extended Data Table 3 Bulk compositions of the $(CoCrNi)_{94}Al_3Ti_3$ APT dataset

Ion Type	Ion Count	Ionic Fraction (%)	Ionic Fraction (sigma) (%)
**Ni**	302294	41.84	0.091
**Co**	174150	24.10	0.064
**Cr**	148163	20.50	0.059
**Ti**	52535	7.27	0.033
**Al**	38663	5.35	0.028
**O**	1839	0.25	0.0059
**S**	1719	0.24	0.0057
**C**	1658	0.23	0.0056
**H**	1132	0.16	0.0047
**D**	362	0.050	0.0026
**Noise**	68	0.0094	0.0011

Extended Data Table 4 Bulk compositions of Inconel 718 superalloy APT dataset

Ion Type	Ion Count	Ionic Fraction (%)	Ionic Fraction (sigma) (%)
**Ni**	1152070	50.10	0.057
**Cr**	464239	20.19	0.032
**Fe**	454568	19.77	0.032
**Nb**	121210	5.27	0.016
**Mo**	43455	1.89	0.0092
**Al**	29287	1.27	0.0075
**Ti**	25513	1.11	0.0070
**Si**	5950	0.26	0.0034
**H**	1592	0.069	0.0017
**O**	538	0.023	0.0010
**P**	530	0.023	0.0010
**D**	365	0.016	0.00083
**Noise**	87	0.0038	0.00041

Second, to evaluate the influence of background signals, we selected a peak-free mass-to-charge-state-ratio window around 55 Da (here selected) and used it to estimate the noise/background contribution, as shown in Extended Data Fig. 17. The corresponding bulk composition analyses in Extended Data Tables 2-4 show that this background contribution is negligible in all representative datasets, indicating that the reported deuterium enrichment is not dominated by background signals.

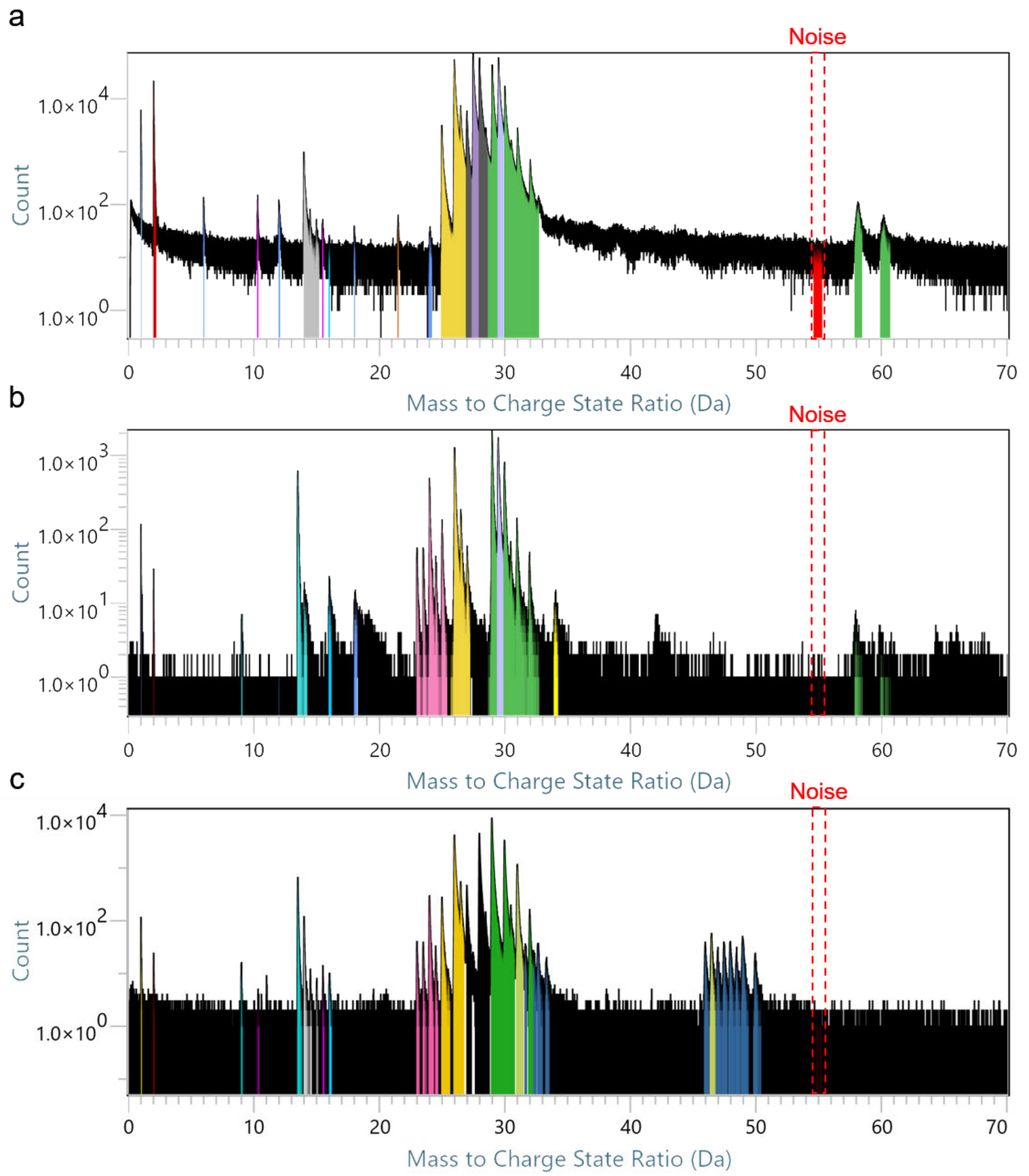

Extended Data Fig. 17 Assessment of peak ranging and background signal in representative APT datasets. Mass spectra of representative datasets from **a** CoCrFeMnNi, **b** $(CoCrNi)_{94}Al_3Ti_3$ and **c** Inconel 718 in main text. The red dashed region marks a peak-free mass-to-charge-state-ratio window around 55 Da, which was used to estimate the background/noise contribution.

Third, we provided the reconstruction parameters used for all datasets in the Methods. The revised Methods section now states as below.

Page 49 Line 3-15:

The image compression factor (ICF) and *k* factor used for reconstruction were 1.3 and 3.3 for the CoCrFeMnNi alloy and the Inconel 718 nickel-based superalloy, and 1.5 and 3.3 for the $(CoCrNi)_{94}Al_3Ti_3$ alloy. These reconstruction parameters were selected following standard APT reconstruction procedures and, for the precipitate-containing datasets, refined by comparison with precipitate morphologies independently observed by TEM. Peak ranging was performed by considering the full isotopic distributions of the major

alloying elements and possible molecular ions, the bulk composition analysis hence this point in Extended Data Tables 2-4. A peak-free around 55 Da was used to estimate the background contribution as shown in Extended Data Fig. 17. APT reconstruction sensitivity was further evaluated by reconstructing the dataset shown in Fig. 3 using an alternative ICF value of 1.3, which did not alter the observed preferential deuterium enrichment within the $L1_2$ precipitate (Extended Data Fig. 18).

Page 43:

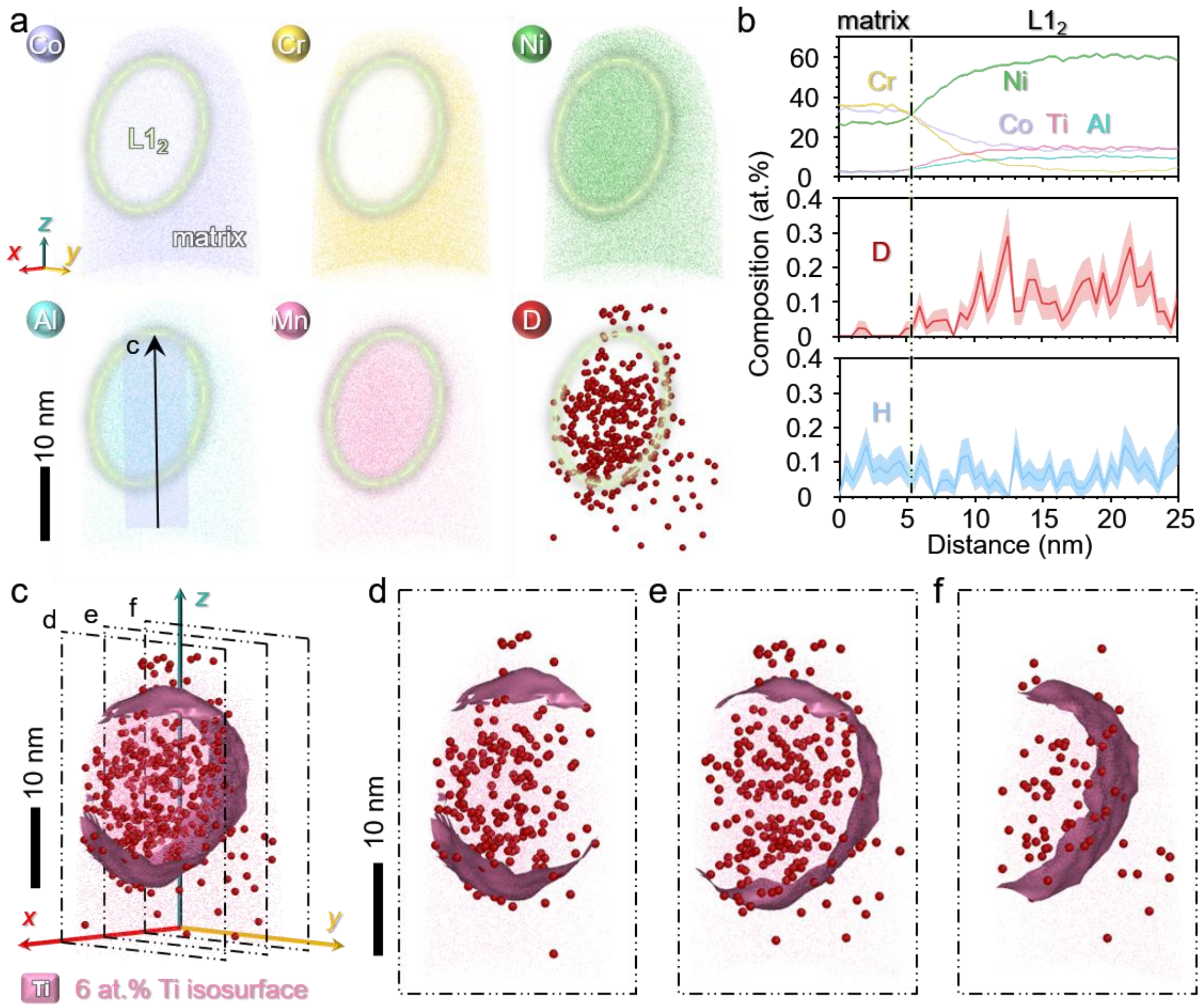

Extended Data Fig. 18 Reconstruction-parameter sensitivity of deuterium partitioning in the $(CoCrNi)_{94}Al_3Ti_3$ alloy. The same APT dataset was reconstructed using a different image compression factor (ICF = 1.3). Although the reconstructed specimen geometry changes slightly, deuterium remains preferentially enriched within the $L1_2$ precipitate, with a similar concentration to that obtained from the reconstruction used in the Fig. 3 of main text (ICF = 1.5). This indicates that reasonable variations in reconstruction parameters do not alter the conclusion that deuterium is preferentially enriched within the $L1_2$ precipitate.

Together, these additional analyses show that the observed phase-dependent deuterium partitioning is not an artefact of peak ranging, background signal or specific reconstruction parameter set. The presented APT analyses are in good rigor.

Minor Comments;

1) Language should be moderated (avoid "unambiguous", "direct evidence").

We have revised the language throughout the manuscript to moderate the level of certainty and removed or replaced terms such as "unambiguous" and "direct evidence" where appropriate.

2) TDS comparison is indirect due to different protocols.

We acknowledge that when charging TDS specimens we must use different parameters to the one for APT specimens, such as longer charging duration. However, the generic purpose of the charging approaches is to introduce sufficient amount of hydrogen and to capture the hydrogen desorption behaviors. We are sure we have followed the common and best practices to our best knowledge. We have thus added additional content to clarify the reviewer's comment as below:

Page 14 Line 2 to 6:

Although the experimental approaches are different, we found these TDS results are consistent with the trends predicted by our first-principles calculations using density functional theory (DFT) for evaluating the solution energies of hydrogen in all relevant interstitial sites (i.e., both octahedral and tetrahedral), as shown in Extended Data Fig. 2b. and

Page 14 Line 11 to 13:

These complementary experimental and computational observations motivated a microscopic APT study to further investigate hydrogen distribution at the nanoscale.

3) More details on statistics and reproducibility are needed.

We thank the reviewer for this suggestion. To further demonstrate reproducibility and generality, we have added additional APT datasets and repeat measurements for all alloy systems investigated. Consistent random deuterium distributions were observed in CoCrNiFeMn, additional solution-treated CoNiV and $(CoCrNi)_{94}Al_3Ti_3$ alloys (Extended Data Figs. 1, 3 and 4), while reproducible deuterium partitioning was observed in the precipitate-containing $(CoCrNi)_{94}Al_3Ti_3$ and Inconel 718 alloys (Extended Data Figs. 8 and 15). The corresponding results and discussion have been added to the revised manuscript.

To conclude, the manuscript provides a significant contribution by demonstrating phase-dependent deuterium partitioning in FCC alloys. However, it does not yet prove unambiguously that the signal corresponds exclusively to interstitial hydrogen. The issue is not the absence of results, but the excess of certainty in their interpretation.

Here is a list of references that may help the authors address these points:

1.Fukai, Y. The Metal–Hydrogen System: Basic Bulk Properties, Springer Series in Materials Science, Springer, Berlin/Heidelberg, 1993, re edition 2005.

2.Fukai, Y. "Formation of superabundant vacancies in M–H alloys and some of its consequences: a review." Journal of Alloys and Compounds 356-357 (2003) 263–269.

3.Fukai, Y. and Okuma, N. "Evidence of copious vacancy formation in Ni and Pd under a high hydrogen pressure." Japanese Journal of Applied Physics 32 (1993), L1256 - L1258

4.Kirchheim, R. "Reducing grain boundary, dislocation line and vacancy formation energies by solute segregation. I. Theoretical background." Acta Mater 55 (2007), 5129–5138.

5.Nagumo, M. "Hydrogen related failure of steels - a new aspect." Mater Sci and Technology 20 (2004), 940-950.

6. Jena, P. and Sinha, A. K. "Positron annihilation in metal-vacancy–hydrogen complexes." Phys Rev B 24 (1981) 2884-2894.

7. An, X. et al. "Investigation of the interaction between hydrogen and vacancies by positron annihilation spectroscopy" Int J of Hydrogen Energy 46 (2021).

8. Gault, B. et al. "Towards Establishing Best Practice in the Analysis of Hydrogen and Deuterium by Atom Probe Tomography." Microscopy and Microanalysis 30 (2024)

9. London, A. J. "Quantifying Uncertainty from Mass-Peak Overlaps in Atom Probe Microscopy." Microscopy and Microanalysis 25 (2019) 378–388.

10. Mouton, I. et al. "Quantification Challenges for Atom Probe Tomography of Hydrogen and Deuterium in Zircaloy-4." Microscopy and Microanalysis 25 (2019) 481-488.

11. Saksena, A. et al. "Optimizing site-specific specimen preparation for atom probe tomography by using hydrogen for visualizing radiation-induced damage." Int. J of Hydrogen Energy 50 (2024), 165-174.

12.Wang, S. et al. "Systematic quantification of hydrogen in pipeline steel by atom probe tomography after ambient charging and transfer." Materials Degradation 8(2024)

13. Vukkum, V. B. et al. "Insights from quasi-in situ cryogenic-transfer atom probe tomography for analyzing hydrogen diffusion in metallic alloys " npj Materials Degradation 9 (2025).

14. Hermida, J. D. and Roviglione, A. "Stacking fault energy decrease in austenitic stainless steels induced by hydrogen pairs formation." Scripta Mater 39 (1998) 1145-1149.

15. He, Y. et al. "Effect of hydrogen atom and hydrogen-filled vacancies on stacking fault energy in γ-Fe by first-principles calculations." Int J of Hydrogen Energy 44 (2019), 17136-17145.

16. Xie, Z. et al. "Sluggish hydrogen diffusion and hydrogen decreasing stacking fault energy in a high-entropy alloy." Materials Today Communications 26 (2021)

17. Ren, X. L. et al. "Hydrogen solution in high-entropy alloys." Phys Chemistry Chemical Physics 23

(2021).

18. Wang, Y. Z. et al. "Disentangling diffusion heterogeneity in high-entropy alloys." Acta mater 222 (2022).

19. Li, Q.-J. et al. "Strengthening in multi-principal element alloys with local-chemical-order roughened dislocation pathways." Nature Communications 10 (2019) 3563.

20. Palumbo, O. et al. "Hydrogen and Deuterium Solubility, Diffusivity and Permeability from Sorption Measurements in the Ni33Ti39Nb28 Alloy." Molecules 28 (2023) 1082.

21. Bourgeois, N. et al. "Vibration analysis of hydrogen, deuterium and tritium in metals: consequences on the isotope effect." Journal of Physics: Condensed Matter 30 (2018)

22. Setyawan, W. et al. "Influence of hydrogen isotopes on vacancy formation and antisite defect diffusion in Pd and V." Computational Materials Science 197 (2021).

23. Nazarov, R., Hickel, T. and Neugebauer, J. "First-principles study of the thermodynamics of hydrogen-vacancy interaction in fcc iron." phys rev B 82 (2010) 224104.

24. Wang, Y. et al. "Hydrogen influence on diffusion in nickel from first-principles calculations." Phys Rev B 91 (2015) 094106.

25. Song, J. and Curtin, W. A. "Atomic mechanism and prediction of hydrogen embrittlement in iron." Nature Materials 12 (2013), 145-151.

26. Zhou, X. et al. "Mechanism and Prediction of Hydrogen Embrittlement in fcc Metals." Phys Rev Letters 127 (2021), 175501.

27. Cheng, H. et al. "The influence of $L1_2$ ordered precipitates on hydrogen embrittlement behavior in CoCrNi-based medium entropy alloys." Acta Mater 260 (2023), 119328.

28. Yoo, J. et al. "Effects of lattice misfit of γ/γ' phases on hydrogen embrittlement behavior in Ni-based single crystal superalloy " J of Materials Research and Technology (2024).

29. Shi, H. et al. "In situ investigation of the interaction between hydrogen and stacking faults in a bulk austenitic steel" Acta Mater(2024)

We thank the reviewer for the advice on excessive certainty and have incorporated necessary changes as summarized in previous responses. We are also grateful for the suggested literature and have reviewed all of them and incorporated them at wherever relevant throughout the revised manuscript. In particular, these references are now cited in the discussions of vacancy and hydrogen–vacancy complexes, APT hydrogen quantification and best practices, stacking-fault energy and defect effects in FCC alloys, isotope effects and the possible role of coherent precipitates and elastic fields. These references indeed helped us improve the quality of manuscript and broaden the discussion of the present results.

References for the response to Reviewer 2's comments:

1 Ren, X. L. et al. Hydrogen solution in high-entropy alloys. Physical Chemistry Chemical Physics 23, 27185-27194 (2021). https://doi.org/10.1039/d1cp04151g

2 Ren, X. L. et al. Swamps of hydrogen in equiatomic FeCuCrMnMo alloys: First-principles calculations. Acta Materialia 180, 189-198 (2019). https://doi.org/https://doi.org/10.1016/j.actamat.2019.09.014

3 Luo, Z., Gao, W. & Jiang, Q. Determinants of vacancy formation and migration in high-entropy alloys. Science Advances 11, eadr4697 (2025). https://doi.org/doi:10.1126/sciadv.adr4697

4 Zhao, S., Egami, T., Stocks, G. M. & Zhang, Y. Effect of d electrons on defect properties in equiatomic NiCoCr and NiCoFeCr concentrated solid solution alloys. Physical Review Materials 2, 013602 (2018). https://doi.org/10.1103/PhysRevMaterials.2.013602

5 Wang, C. et al. First-principles study of hydrogen-vacancy interactions in CoCrFeMnNi high-entropy alloy. Journal of Alloys and Compounds 922, 166259 (2022). https://doi.org/https://doi.org/10.1016/j.jallcom.2022.166259

6 Nazarov, R., Hickel, T. & Neugebauer, J. First-principles study of the thermodynamics of hydrogen-vacancy interaction in fcc iron. Physical Review B 82, 224104 (2010). https://doi.org/10.1103/PhysRevB.82.224104

7 Guan, H. et al. Chemical environment and magnetic moment effects on point defect formations in CoCrNi-based concentrated solid-solution alloys. Acta Materialia 187, 122-134 (2020). https://doi.org/https://doi.org/10.1016/j.actamat.2020.01.044

8 Setyawan, W. & Senor, D. J. Influence of hydrogen isotopes on vacancy formation and antisite defect diffusion in palladium and vanadium metals. Computational Materials Science 197, 110641 (2021). https://doi.org/https://doi.org/10.1016/j.commatsci.2021.110641

9 Counts, W. A., Wolverton, C. & Gibala, R. First-principles energetics of hydrogen traps in α-Fe: Point defects. Acta Materialia 58, 4730-4741 (2010). https://doi.org/https://doi.org/10.1016/j.actamat.2010.05.010

10 Jiang, D. E. & Carter, E. A. Diffusion of interstitial hydrogen into and through bcc Fe from first principles. Physical Review B 70, 064102 (2004). https://doi.org/10.1103/PhysRevB.70.064102

Response letter

Editor/Reviewers' comments in ***italic blue***, authors' responses in **black**, and revised content as in the revised manuscript in **highlighted black**

Referee #2 (Remarks to the Author):

The authors have made a substantial effort to answer the reviewers' comments. I acknowledge the important additional work, including new APT datasets, quasi in situ TEM, positron annihilation measurements and additional DFT calculations. These new results reinforce the evidence for reproducible phase dependent deuterium partitioning. However, they do not demonstrate without ambiguity that the detected deuterium is located predominantly at defect-free interstitial sites. The main issue therefore remains the level of certainty used in the manuscript.

As shown by the yellow-highlighted parts in the revised manuscript, we have moderated our tone and acknowledge the potential contributions of elastic fields and vacancies.

Major Comments:

1) The title, abstract, introduction and conclusion still use strong expressions such as "observation of interstitial hydrogen," "direct experimental evidence" and "directly probe hydrogen in solid solution." More cautious wording is needed, as the results show deuterium partitioning consistent with interstitial hydrogen in solid solution, without directly identifying its atomic state.

To address the reviewer's concern, we have carefully revised text in the according sections. The changes are summarized below.

Page 1 Line 1:

Toward observing interstitial hydrogen in solution in face-centered cubic metal lattices

Page 2 Line 12:

These findings demonstrate that APT can move beyond identifying hydrides and solute-decorated defects to probe hydrogen in solid solution, providing new insight into hydrogen partitioning in structural alloys and informing materials design for hydrogen-related applications.

Page 18 Line 14:

Second, to probe vacancy populations, we performed positron annihilation measurements…

Page 18 Line 20:

This indicates that the vacancy concentrations in these alloys are low.

Page 19 Line 17:

Together, the thermodynamic analysis, DFT calculations, quasi in-situ TEM observations during D charging,

and positron annihilation measurements support the interpretation that the detected D is predominantly associated with interstitial lattice sites in the $L1_2$ phase, although a minor contribution from defect-related trapping (e.g., isolated vacancies, vacancy-H_n/D_n complexes, or elastic fields) and intrinsic limitations associated with APT measurements cannot be completely excluded [11,35,46-48].

Page 20 Line 12:

This co-precipitation strategy enabled both phases of interest to appear within the small APT field of view, allowing a comparison under identical experimental conditions.

Page 23 Line 1 to Line 11:

In summary, we examined a set of FCC MPEAs, in both bulk-matrix and precipitate forms, and showed that signatures of interstitial hydrogen (represented by deuterium) in solid solutions can be probed by APT. The experimental observations agree with our DFT predictions of chemistry-dependent hydrogen solution energies. Our observations of deuterium partitioning are consistent with interstitial hydrogen in solid solution in the MPEAs, while minor contributions from isolated vacancies, vacancy-H_n/D_n complexes, or elastic fields cannot be fully excluded, further show how chemical affinity and crystal structure can influence the distribution of hydrogen in materials. These results demonstrate an experimental protocol to probe hydrogen in solid solutions and develop an approach to connect atomistic predictions and experimental observation of hydrogen behavior in materials.

2) The additional TEM and positron measurements make dominant defect trapping unlikely, but do not fully exclude isolated vacancies or V-H_n/V-D_n complexes. The equilibrium vacancy calculation at 800 ℃ is not directly applicable to defects generated during non-equilibrium room-temperature charging. Moreover, the small differences between the H-charged and pristine PAS curves should be quantified, together with the detection limit. The manuscript should therefore conclude that vacancy trapping is unlikely to be dominant, rather than excluded.

Regarding the comment on the equilibrium vacancy calculation at 800 °C, we agree with the reviewer and have removed this discussion from the revised manuscript.

With respect to the TEM results, the primary challenge is the lack of mature experimental techniques capable of directly observing isolated vacancies or V-H_n/V-D_n complexes under the present conditions. To the best of our knowledge, no existing experimental method can unambiguously quantify atomic-scale vacancies and/or V-H_n/V-D_n complexes in materials. We believe that we have followed current best practice in this regard. Nevertheless, in response to Question 1, we have toned down our claims regarding direct experimental proof in the revised manuscript.

Regarding the PAS quantification, as shown in Extended Data Fig. 11(a), we cannot identify any significant difference between the S-parameter profiles of the H-charged specimen and the pristine specimen. The only exception occurs in the near-surface region, between 0 and approximately 40 nm (corresponding to an incident positron energy of 0.5-4 keV), where the measurements carry substantial uncertainty. Interestingly, within this region, the positron signal from the H-charged specimen is even lower than that from the pristine specimen. This observation suggests that the vacancy concentration in the H-charged specimen is not higher than that in the pristine specimen.

Nevertheless, we attempted to present the data in a more quantitative manner, as shown in Fig. R1. Specifically, we normalised the S-parameter intensity of the H-charged and Ni-irradiated specimens using the corresponding signal from the pristine specimen. For the H-charged specimen, we calculated the relative difference, $\Delta S/S_{pristine}$, where ΔS represents the difference between the S-parameters of the H-charged and pristine specimens. The resulting data are shown in blue in Fig. R1. An identical analysis was applied to the Ni-irradiated specimen, yielding the red data in Fig. R1. The results demonstrate that the signal from the H-charged specimen remains largely indistinguishable from that of the pristine specimen, except in the near-surface region where the uncertainty is substantial. This is in stark contrast to the Ni-irradiated specimen, which exhibits a significantly higher positron signal than the pristine specimen. Because this data processing does not alter the main conclusion of the study, we prefer to present the original data in the manuscript, as it is more straightforward for readers to interpret. We appreciate the reviewer's understanding of our intention to maximise clarity and accessibility for the broader readership.

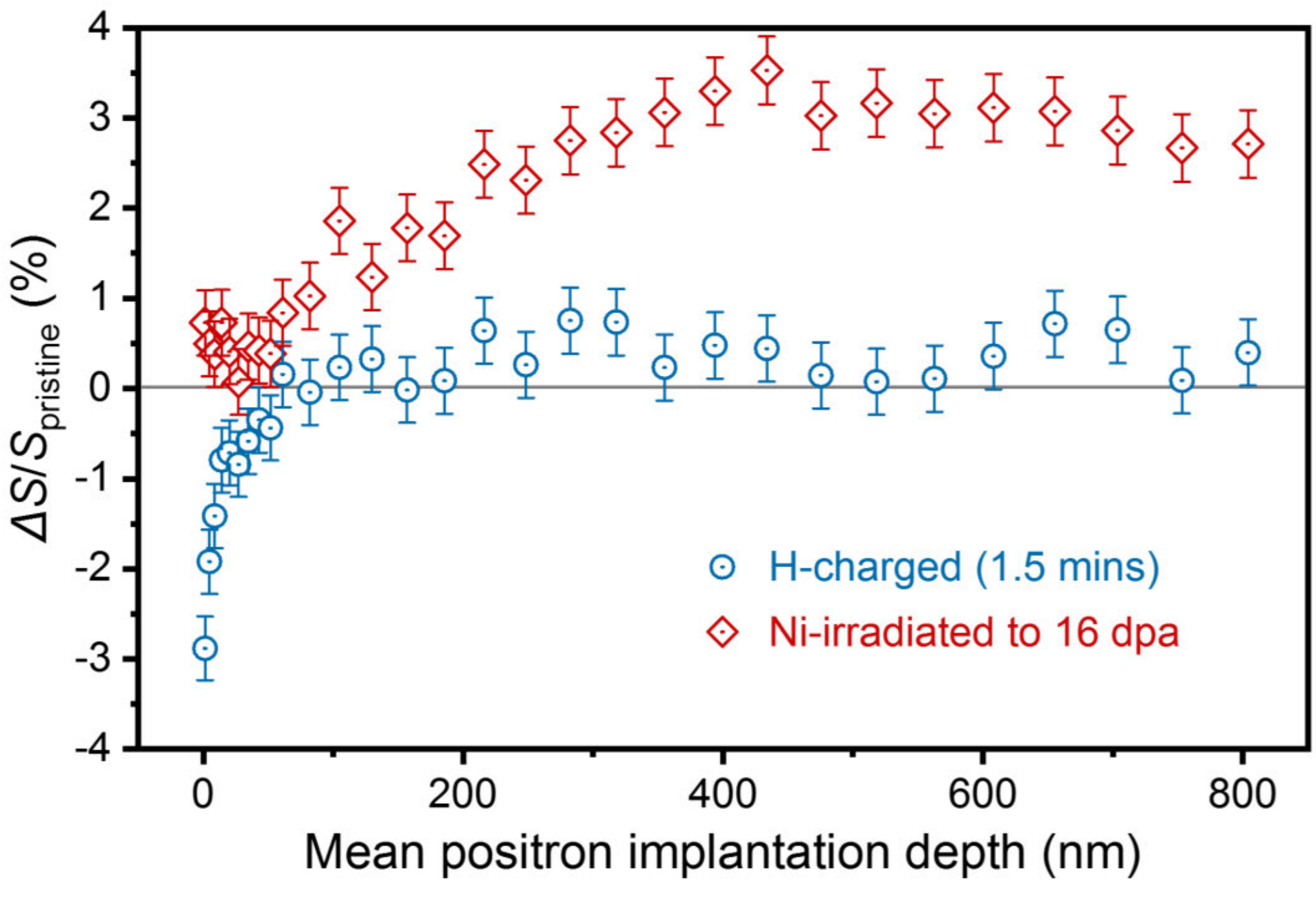

Fig. R1 Depth-dependent Doppler-broadening positron annihilation spectroscopy (PAS) results for the pristine, hydrogen-charged and Ni-ion-irradiated $(CoCrNi)_{94}Al_3Ti_3$ alloy with $L1_2$ precipitates.

3) The 10s hold at room temperature promotes preferential deuterium desorption from the matrix. The measured distribution therefore reflects both thermodynamic and kinetic effects, rather than the state immediately after charging. The possible influence of elastic fields around the coherent precipitates should also be discussed.

We thank the reviewer's reminder about the data reflecting both kinetic and thermodynamic effects. As below, we have revised the manuscript to explicitly state that the measured distribution reflects a combination of both thermodynamic and kinetic effects, rather than the transient state immediately following electrochemical charging.

Page 14 Line 18 to Page 15 Line 5:

This extended room-temperature holding was intended to promote preferential deuterium desorption from the matrix, thereby enhancing the deuterium concentration contrast between phases with low (matrix) and high (precipitate) hydrogen-binding energies in the APT dataset. After saturating the specimens with deuterium, this controlled holding allows the charged deuterium atoms to remain mobile briefly before quenching, leading to more pronounced deuterium loss from the low–binding-energy phase than from the high–binding-energy phase. Consequently, the measured deuterium distribution reflects both thermodynamic and kinetic effects during this brief redistribution.

Regarding the potential influence of elastic strain fields around coherent precipitates, we have acknowledged its possibility in the revised manuscript as detailed below.

Page 19 Line 17:

Together, the thermodynamic analysis, DFT calculations, quasi in-situ TEM observations during D charging, and positron annihilation measurements support the interpretation that the detected D is predominantly associated with interstitial lattice sites in the $L1_2$ phase, although a minor contribution from defect-related trapping (e.g., isolated vacancies, vacancy-H_n/D_n complexes, or elastic fields) and intrinsic limitations associated with APT measurements cannot be completely excluded [11,35,46-48].

Page 23 Line 1 to Line 11:

In summary, we examined a set of FCC MPEAs, in both bulk-matrix and precipitate forms, and showed that signatures of interstitial hydrogen (represented by deuterium) in solid solutions can be probed by APT. The experimental observations agree with our DFT predictions of chemistry-dependent hydrogen solution energies. Our observations of deuterium partitioning are consistent with interstitial hydrogen in solid solution in the MPEAs, while minor contributions from isolated vacancies, vacancy-H_n/D_n complexes, or elastic fields cannot be fully excluded, further show how chemical affinity and crystal structure can influence the distribution of hydrogen in materials. These results demonstrate an experimental protocol to

probe hydrogen in solid solutions and develop an approach to connect atomistic predictions and experimental observation of hydrogen behavior in materials.

4) The treatment of isotope effects needs clarification. Static DFT energies and classical NEB barriers are normally independent of isotope mass. H/D differences require vibrational and ZPE corrections, including at the transition state. The authors should explain how the different H and D curves were obtained or restrict their conclusion to general energetic trends.

Indeed, the results we presented in the Extended Data Fig. 20 (unchanged) at Page 44 in the revised manuscript show the static DFT energy difference between hydrogen isotopes is negligible. Regarding the H/D difference in diffusion considering vibrational and ZPE corrections, we have revised the Extended Data Fig. 21 at Page 45 in the revised manuscript per the reviewer's comment, which is also shown below.

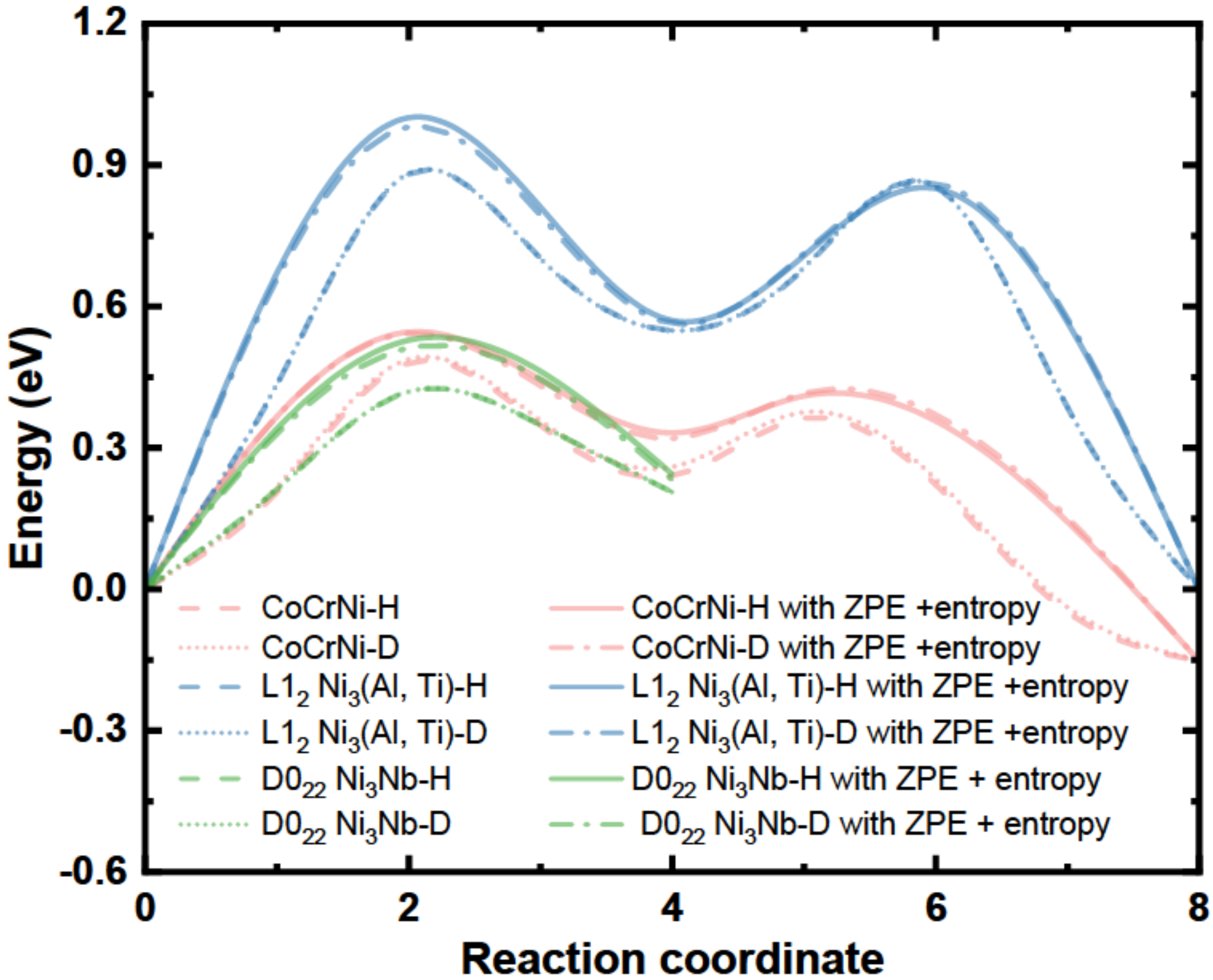

Extended Data Fig. 21 Hydrogen/Deuterium diffusion pathways in CoCrNi (pink), L1$_2$ Ni$_3$(Al, Ti) (blue), and D0$_{22}$ Ni$_3$Nb (green) with and without ZPE corrections.

We noticed the correction-incorporated diffusion curves of H and D from octahedral to octahedral (oct–oct) in L1$_2$ Ni$_3$(Al, Ti), as shown by the blue solid and broken lines, respectively, exhibit only a slight difference; while the curves of the other two phases (CoCrNi and D0$_{22}$ Ni$_3$Nb) nearly coincided, as shown by the pink and green data, respectively. We found that the energy difference arises from the slight differences in the initial and final configurations between H and D. When we used identical initial and final structures, H and D present consistent diffusion curves in L1$_2$ Ni$_3$(Al, Ti). We have added corresponding description at Page 55 in the revised manuscript as detailed below.

Page 54 Line 21 to Page 55 Line 4:

After applying the corrections, the saddle points of the diffusion curves of all the three phases (CoCrNi, $L1_2$ $Ni_3(Al, Ti)$, and $D0_{22}$ Ni_3Nb) shift upward slightly, with minor differences observed between H and D. The general energetic trend remains the same after the corrections.

5) The APT uncertainty discussion should focus more directly on the low-mass region. A background noise measured around 55 Da does not quantify possible overlap between D^+ and H_2^+ at 2 Da. The authors should provide the low-mass spectra, the number of independent tips, the D concentration for each replicate and a distinction between counting and systematic uncertainties.

We thank the reviewer pointing out this. In Extended Data Fig. 17 at Page 42 in the revised manuscript as well as below, we now use the low-mass region to estimate the noise that can obscure D^+/H_2^+ signal near 2 Da, specifically at a peak-free region around 2.5 Da, close to the 2 Da peak.

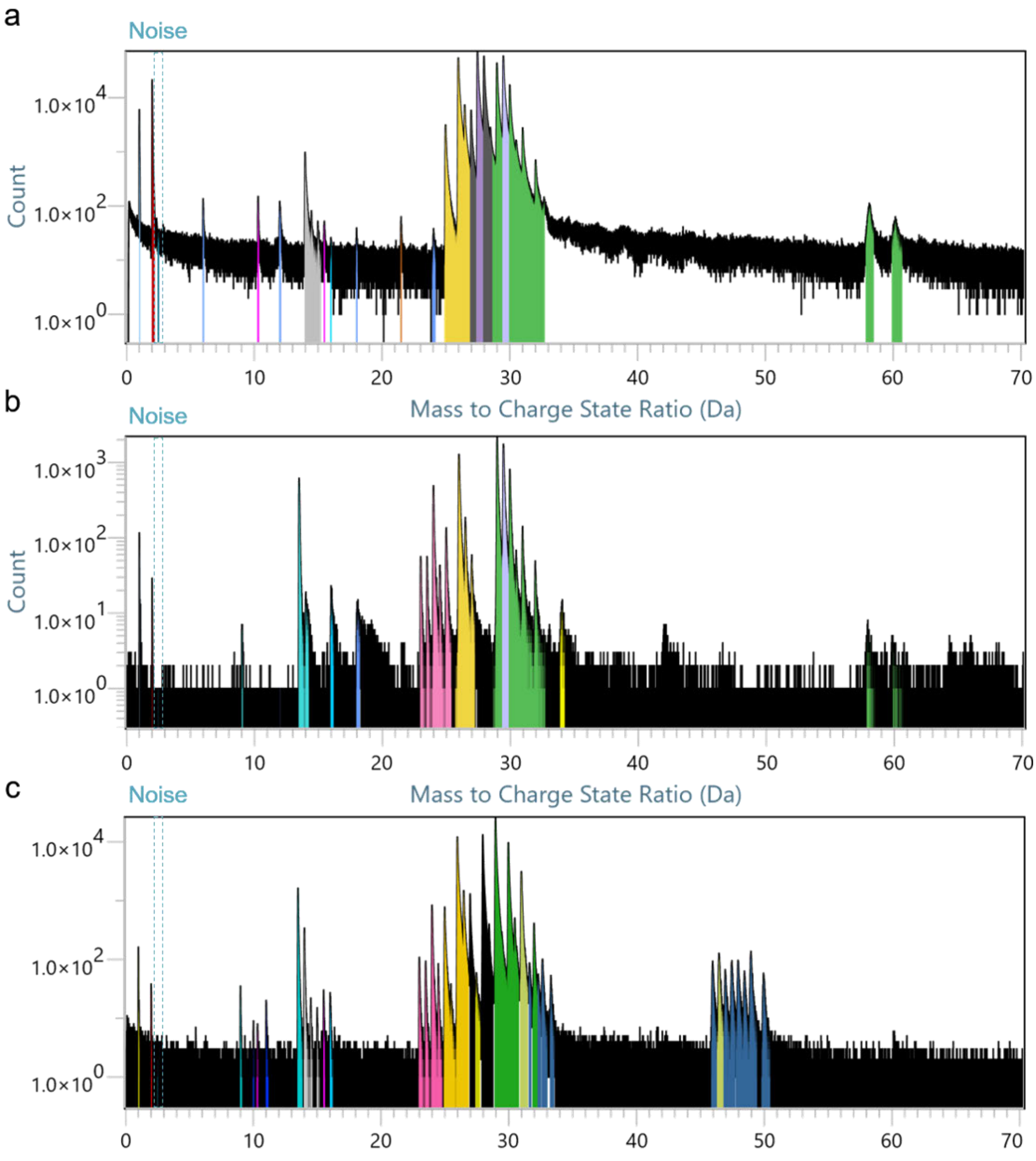

Extended Data Fig. 17 Assessment of peak ranging and background signal in representative APT datasets. Mass spectra of representative datasets from **a** CoCrFeMnNi, **b** $(CoCrNi)_{94}Al_3Ti_3$ and **c** Inconel 718 in main text. The red dashed region marks a peak-free mass-to-charge-state-ratio window around 55 Da, which was used to estimate the background/noise contribution.

Based on this revised treatment, the corresponding noise estimation is presented in Extended Data Tables 2–4 at Pages 40 and 41 in the revised manuscript. The results show that the background remains significantly lower than the 2 Da signals, at least by one order of magnitude. Therefore, the contribution of local counting/background noise to the measured D signal is generally minor and is unlikely to affect the observed D partitioning.

Extended Data Table 2 Bulk composition of D-charged CoCrFeMnNi APT dataset

Ion Type	Ion Count	Ionic Fraction (%)	Ionic Fraction (sigma) (%)
**Ni**	7981596	22.54	0.0088
**Mn**	6997942	19.77	0.0082
**Fe**	6914582	19.53	0.0081
**Cr**	6534182	18.46	0.0079
**Co**	6228282	17.59	0.0076
**D**	510975	1.44	0.0020
**Si**	99307	0.28	0.00089
**H**	97449	0.28	0.00088
**C**	21901	0.062	0.00042
**P**	10030	0.028	0.00028
**Noise**	3437	0.0097	0.00017
**PC2**	3060	0.0086	0.00016
**O**	1567	0.0044	0.00011

Extended Data Table 3 Bulk compositions of the D-charged $(CoCrNi)_{94}Al_3Ti_3$ APT dataset

Ion Type	Ion Count	Ionic Fraction (%)	Ionic Fraction (sigma) (%)
**Ni**	302294	41.84	0.091
**Co**	174150	24.10	0.064
**Cr**	148163	20.50	0.059
**Ti**	52535	7.27	0.033
**Al**	38663	5.35	0.028
**O**	1839	0.25	0.0059
**S**	1719	0.24	0.0057
**C**	1658	0.23	0.0056
**H**	1132	0.16	0.0047
**D**	362	0.050	0.0026
**Noise**	10	0.0014	0.00044

Extended Data Table 4 Bulk compositions of D-charged Inconel 718 superalloy APT dataset

Ion Type	Ion Count	Ionic Fraction (%)	Ionic Fraction (sigma) (%)
**Ni**	1152070	50.10	0.057
**Cr**	464239	20.19	0.032
**Fe**	454568	19.77	0.032
**Nb**	121210	5.27	0.016
**Mo**	43455	1.89	0.0092
**Al**	29287	1.27	0.0075
**Ti**	25513	1.11	0.0070
**Si**	5950	0.26	0.0034
**H**	1592	0.069	0.0017

**O**	538	0.023	0.0010
**P**	530	0.023	0.0010
**D**	365	0.016	0.00083
**Noise**	43	0.0019	0.00029

Regarding D concentration in each replicate, Extended Data Table 5 at Page 41 in the revised manuscript as well as below presents the primary results from the 8 successful cryo-APT datasets out of our 68 trials reported in this manuscript by using the same data analysis protocol. For each dataset, we present the total ion count, 2 Da ion count, measured 2 Da concentration and its statistical uncertainty, together with the corresponding local background count (noise), background fraction and uncertainty (sigma). Related descriptions as detailed below are also added. We hope this clarifies the reviewer's concern.

Extended Data Table 5 Summary of the 2 Da signal and local low-mass noise analysis for the 8 successful cryo-APT datasets out of 68 trials. The table reports the corresponding dataset, total ion count, 2 Da ion count, measured 2 Da ionic fraction and statistical uncertainty, together with the ion count, ionic fraction and statistical uncertainty measured from the peak-free noise region around 2.5 Da.

No.	Corresponding Dataset	Original Dataset	Total Ion Count	2 Da Ion Count	2 Da Ionic Fraction (%)	2 Da Ionic Fraction (sigma) (%)	Noise Ion Count	Noise Ionic Fraction (%)	Noise Ionic Fraction (sigma) (%)
1	Fig. 1	R18_60357	35404310	510975	1.44	0.0020	3437	0.0097	0.00017
2	Fig. 3	R18_63173	722525	362	0.050	0.0026	10	0.0014	0.00044
3	Fig. 4	R18_64108	2299358	365	0.016	0.00083	43	0.0019	0.00029
4	Extended Data Fig. 1	R18_60355	13208238	190823	1.45	0.0033	786	0.0060	0.00021
5	Extended Data Fig. 3	R18_64543	9925962	288199	2.90	0.0055	266	0.0027	0.00016
6	Extended Data Fig. 4	R18_64538	32513297	38913	0.12	0.00061	617	0.0019	0.000076
7	Extended Data Fig. 8	R18_63174	4991869	390	0.0078	0.00040	85	0.0017	0.00019
8	Extended Data Fig. 15	R18_64128	6107879	610	0.010	0.00040	78	0.0013	0.00015

Page 49 Line 11 to Line 18:

A peak-free region around 2.5 Da was defined as Noise and used to estimate the local low-mass background adjacent to the 2 Da signal. Three representative mass spectra are shown in Extended Data Fig. 17, and the results for all 8 successful cryo-APT datasets out of 68 trials are summarized in Extended Data Table 5. Because D^+ and H_2^+ overlap at 2 Da and cannot be resolved directly, this overlap was treated separately as a systematic uncertainty and assessed using uncharged reference specimens analyzed under identical voltage-pulsing conditions.

Page 19 Line 13 to Line 19:

Together, the thermodynamic analysis, DFT calculations, quasi in-situ TEM observations during D charging, and positron annihilation measurements support the interpretation that the detected D is predominantly associated with interstitial lattice sites in the $L1_2$ phase, although a minor contribution from defect-related trapping (e.g., isolated vacancies, vacancy-H_n/D_n complexes, or elastic fields) and intrinsic limitations associated with APT measurements cannot be completely excluded [11,35,46-48].

Minor Comments;

1) Regarding page 20, I am not familiar with $Ni_{94}(Al,Ti)L12$ precipitates; perhaps they are $Ni_3(Al,Ti)L1_2$?

We thank the reviewer for pointing this out and have corrected this as "Ni_3(Al, Ti) $L1_2$" in the revised manuscript, at Page 19 Line 21.

2) On page 49, I see "steel samples" those aren't steels!

We thank the reviewer for pointing this out and have corrected as "the samples" in the revised manuscript, Page 50 Line 3.

3) The manuscript still contains several grammatical errors and should be carefully checked.

We have checked the entire manuscript again and fixed the grammatical errors and punctuation typos to our best knowledge as detailed below.

Page 14 Line 10:

…at the specific octahedral sites of the $L1_2$ Ni_3(Al, Ti) phase.

Page 23 Line 9:

These results demonstrate an experimental protocol to probe hydrogen in solid solutions…

Page 26 Line 8:

…for hydrogen solutions are…

Page 38 Line 6:

…10 nm-thick slices displaying the Al and Nb isosurfaces…

Page 43 Line 7:

…reconstruction used in Fig. 3 of main text…

Page 49 Line 4:

The image compression factors (ICF) and *k* factors used…
